# Overview of Tensor-Based Cooperative MIMO Communication Systems—Part 2: Semi-Blind Receivers

**DOI:** 10.3390/e26110937

**Published:** 2024-10-31

**Authors:** Gérard Favier, Danilo Sousa Rocha

**Affiliations:** 1 I3S Laboratory, Côte d’Azur University, 06903 Sophia Antipolis, France; 2 Federal Institute of Education, Science, and Technology of Ceará, Fortaleza 60040-531, Brazil; danilo.rocha@ifce.edu.br

**Keywords:** cooperative communication systems, MIMO systems, nested tensor models, relaying systems, semi-blind receivers, tensor codings, tensor decompositions, closed-form algorithms

## Abstract

Cooperative MIMO communication systems play an important role in the development of future sixth-generation (6G) wireless systems incorporating new technologies such as massive MIMO relay systems, dual-polarized antenna arrays, millimeter-wave communications, and, more recently, communications assisted using intelligent reflecting surfaces (IRSs), and unmanned aerial vehicles (UAVs). In a companion paper, we provided an overview of cooperative communication systems from a tensor modeling perspective. The objective of the present paper is to provide a comprehensive tutorial on semi-blind receivers for MIMO one-way two-hop relay systems, allowing the joint estimation of transmitted symbols and individual communication channels with only a few pilot symbols. After a reminder of some tensor prerequisites, we present an overview of tensor models, with a detailed, unified, and original description of two classes of tensor decomposition frequently used in the design of relay systems, namely nested CPD/PARAFAC and nested Tucker decomposition (TD). Some new variants of nested models are introduced. Uniqueness and identifiability conditions, depending on the algorithm used to estimate the parameters of these models, are established. Two families of algorithms are presented: iterative algorithms based on alternating least squares (ALS) and closed-form solutions using Khatri–Rao and Kronecker factorization methods, which consist of SVD-based rank-one matrix or tensor approximations. In a second part of the paper, the overview of cooperative communication systems is completed before presenting several two-hop relay systems using different codings and configurations in terms of relaying protocol (AF/DF) and channel modeling. The aim of this presentation is firstly to show how these choices lead to different nested tensor models for the signals received at destination. Then, by capitalizing on these models and their correspondence with the generic models studied in the first part, we derive semi-blind receivers to jointly estimate the transmitted symbols and the individual communication channels for each relay system considered. In a third part, extensive Monte Carlo simulation results are presented to compare the performance of relay systems and associated semi-blind receivers in terms of the symbol error rate (SER) and channel estimate normalized mean-square error (NMSE). Their computation time is also compared. Finally, some perspectives are drawn for future research work.

## 1. Introduction

During the last decade, new technologies have emerged for future sixth-generation (6G) wireless communications and networks. Among these promising technologies, we can mention massive multiple-input multiple-output (MIMO) antenna arrays, three-dimensional (3D) polarized antennas, high-frequency millimeter-wave (mmWave), i.e., terahertz (THz), communications, large intelligent surfaces (LISs), and holographic beamforming (HBF) antennas.

To improve signal coverage, mobile connectivity, latency, reliability, and energy consumption, as well as the quality of service (QoS) of future wireless networks, cooperative communication systems are the subject of tremendous research interests, with the aim of designing network architectures integrating ground, space, air, and underwater networks. These cooperative systems can be classified into the following three basic categories, namely relay-, IRS- (intelligent reflecting surface, also known as reconfigurable intelligent surface (RIS)) and UAV- (unmanned aerial vehicle, i.e., drones) aided systems. For a comparison of relay- and IRS-assisted wireless systems, the reader can refer to [1,2,3,4]. These types of assistance can be combined as, for instance, in [5,6], where IRS passive reflection is combined with active DF (decode and forward) relaying to improve the coverage and rate performance of conventional IRS-assisted systems.

Wireless propagation channel impairments such as multipath fading, delay, and Doppler spreads, which strongly depend on the environment (urban, rural, and indoor), degrade the quality of reception to recover the transmitted information symbols. Enhanced performance of wireless communication systems can be achieved by combining diverse techniques in multiple domains (space, time, frequency, chip, polarization, etc.) in order to exploit, at the receiver, several versions of transmitted symbols. Such symbol repetition can be achieved via the use of multi-antennas at the transmitter and receiver (space diversity), a repetition of the transmission of the same symbols over several time slots (time diversity), and the use of different subcarriers (frequency diversity). Diversity can also be introduced by means of specific codings, such as space-time (ST), space-frequency (SF), and space-time-frequency (STF) codings.

High-order tensors, also known as multiway arrays, are well suited to taking into account multiple types of diversity via the coding of information symbols to be transmitted, representing multidimensional received signals, designing semi-blind receivers, which is to say without a priori knowledge of the channels, and processing blocks of received signals in order to jointly estimate communication channels and transmitted symbols. Note that semi-blind means that only a few pilot symbols are needed to eliminate the scaling ambiguities inherent to the tensor model of the communication system. Besides the obvious potential to represent, compress, analyze, merge, and classify multidimensional, multimodal, and often incomplete data, tensor decompositions have the property of essential uniqueness (up to trivial indeterminacies in terms of permutation and scaling factor ambiguities in the columns of matrix factors) under milder conditions than matrix decompositions, which offers greater flexibility for the choice of design parameters.

Since the pioneering works of [7,8,9,10,11,12] for psychometrics, phonetics, and chemometrics applications, blind source separation (BSS), and wireless communication systems, tensors have been extensively used in various fields of applications, like computer vision [13], ECG, and EEG applications [14,15,16], hyperspectral image classification and anomaly detection [17,18], traffic data completion [19,20,21], recommendation systems [22,23], nonlinear system modeling and identification [24], data mining and data fusion [25,26,27,28], and tensor networks to represent and classify very large arrays of data with applications in machine learning [29,30], among many other applications. For a more complete description of tensor-based signal processing applications, the reader is referred to [31,32,33].

In the context of wireless systems, many tensor-based approaches have been proposed to design both point-to-point systems and cooperative systems, with associated semi-blind receivers. Most tensor-based systems have been designed using the popular tensor decomposition known as PARAFAC (parallel factors analysis) [8] or CPD (canonical polyadic decomposition) [34]. However, over the last two decades, various new tensor models have emerged when designing communication systems, such as the CONFAC (constrained PARAFAC) [35,36], PARATUCK-$\left(N_{1},N\right)$ [37], generalized PARATUCK [38], NCPD (nested CPD) [39,40], nested Tucker decomposition (NTD) [41], coupled NTD [42], and doubly coupled NCPD [43] models. These models mainly depend on the choice of coding, whether or not resource allocation is taken into account, assumptions about communication channels, and the presence or absence of relays, as will be shown in Section 5.

It is fundamental to note that, unlike most tensor-based applications, in the context of wireless communication systems, through construction, all the matrix factors of tensor models are physically interpretable in terms of symbol, coding, or channel matrices, with physical parameters such as fading coefficients, angles of departure and arrival (AoD/AoA), or time delays. In addition, certain matrix or tensor factors of these models are possibly structured, such as, for example, the Vandermonde structure of steering matrices and the orthonormal (Fourier) structure of coding matrices or matrix unfoldings of tensor codings.

The objective of this paper is fourfold:

- To provide a self-contained overview of tensor models used to design wireless communication systems. After a reminder of tensor prerequisites, standard tensor decompositions are first recalled, more particularly the CPD/PARAFAC and Tucker decompositions, as well as some variants. Then, two important classes of tensor decompositions, namely the NCPD and NTD, are presented in an unified and original way, using a new representation by means of graphs and highlighting their link with the tensor train decomposition (TTD) [44]. This link is exploited to demonstrate the uniqueness property of NTD models. Some of the models to be used when designing new relay systems are introduced for the first time.
- To present two families of algorithms that estimate the parameters of NCPD and NTD models: iterative algorithms based on alternating least squares (ALS) and closed-form solutions using Khatri–Rao and Kronecker factorization methods, denoted as KRF and KronF, which consist of SVD-based rank-one matrix or tensor approximations. These closed-form algorithms result from the fact that unfoldings of CPD and TD are expressed in terms of Khatri–Rao and Kronecker products of matrix factors, respectively.
- To provide an overview of tensor-based cooperative MIMO communication systems from a semi-blind receiver perspective, with a focus on two-hop relay systems, as complement of our companion paper [45]. The goal of this presentation is first to show how the choices of coding, relay protocol (AF (amplify-and-forward) or DF), and assumptions made about communication channels (flat fading or frequency-selective fading channels) impact the modeling of relay systems. Then, assuming knowledge of coding tensors and matrices at the destination and exploiting the multilinear structure of the system model, we devise semi-blind receivers to jointly estimate the transmitted symbols and the individual channels for each considered relay system. Uniqueness conditions for the tensor model of each system and necessary conditions for parameter identifiability using the associated receivers are established.
- To compare the performance of main two-hop relay systems and associated semi-blind receivers in terms of the symbol error rate (SER), channel estimate normalized mean-square error (NMSE), and computation time, by means of extensive Monte Carlo simulations, with the aim of showing how the best performance–complexity–identifiability condition tradeoff can be achieved.

The rest of the paper is organized in seven sections. In Section 2, some tensor prerequisites are recalled, including an introduction of index convention and the definition of basic tensor operations in order to make the content as self-contained as possible. Section 3 provides an overview of the main tensor models, with a focus on nested ones, namely the NCPD and NTD, highlighting their link with the TTD and proposing a new representation by means of graphs. New variants of nested models to be used for designing two-hop relay systems are introduced. Uniqueness conditions are established for the nested models considered. In Section 4, several parameter estimation algorithms are described for these models, with particular attention given to closed-form algorithms. The identifiability conditions are established for both ALS and closed-form algorithms. Section 5 is devoted to a comprehensive survey of one-way two-hop relay systems.

After an overview of cooperative systems from a semi-blind receiver point of view, relay systems are classified according to the choice of coding scheme and the resulting tensor model for the signals received at destination. Three classes of coding are considered:

- Tensor-based codings, including TSTF, STSTF, TST, and STST codings (TSTF = tensor space-time frequency; STSTF = simplified TSTF; TST = tensor space-time; STST = simplified TST);
- Matrix-based codings, including DKRSTF and SKRST codings (DKRSTF = doubly Khatri–Rao space-time frequency; SKRST = simplified Khatri–Rao space-time);
- STST and SKRST codings combined with MSMKron and MSMKR codings (MSMKron = multiple symbol matrices’ Kronecker product; MSMKR = multiple symbol matrices’ Khatri–Rao product), respectively.

In Section 6, by capitalizing on the tensor models of the systems, we design semi-blind receivers to jointly estimate transmitted symbols and individual communication channels. In Section 7, extensive Monte Carlo simulation results are presented to compare the performance of the relay systems considered in this overview and their associated semi-blind receivers in terms of SER, channel-estimate NMSE, and computation time. Finally, Section 8 presents some perspectives for future research work.

**Figure 1:**
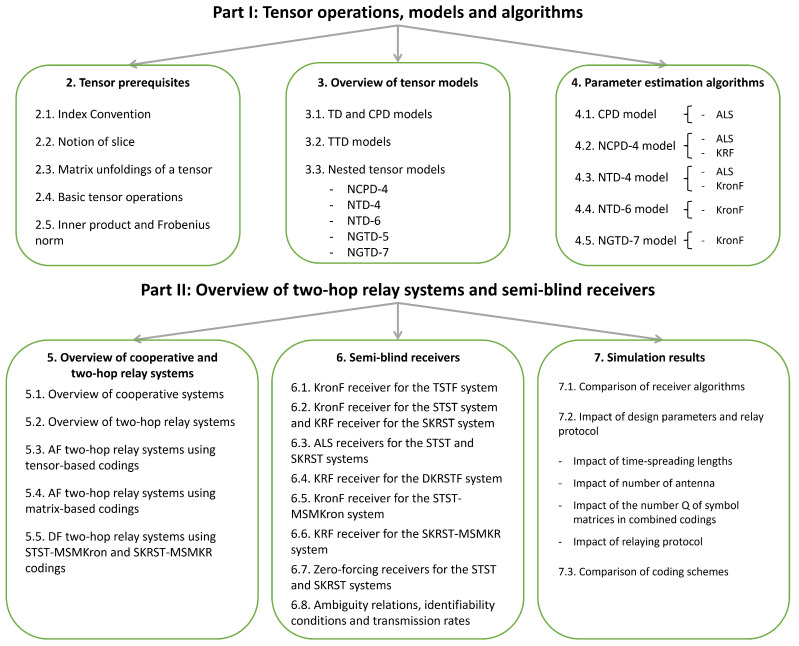
Organization of the paper.

The paper is divided into two main parts, entitled “Tensor operations, models, and algorithms” and “Overview of two-hop relay systems and semi-blind receivers”, composed of three sections each, as detailed in the flow chart of Figure 1.

**Notation and acronyms**: Table 1 summarizes the notations used throughout the paper, and a comprehensive glossary of acronyms is provided in Appendix A.

## 2. Tensor Prerequisites

An *N*th-order tensor, $X\in K^{{\underline{I}}_{N}}$, of size ${\underline{I}}_{N}≜I_{1}\times \cdots \times I_{N}$, will be denoted as $\left[x_{{\underline{i}}_{N}}\right]=\left[x_{i_{1},\cdots ,i_{N}}\right]$, where ${\underline{i}}_{N}≜\left\{i_{1},\cdots ,i_{N}\right\}$, with K=R or C, depending on whether the tensor is real- or complex-valued. Each index, $i_{n}\in \langle I_{n}\rangle ≜\left\{1,\cdots ,I_{n}\right\}$, for n∈〈N〉≜{1,⋯,N}, is associated with the *n*th-mode whose dimension is $I_{n}$. In the context of wireless communications, each mode of the tensor of encoded or received signals is associated with a particular diversity (space, frequency, chip, time slot, or symbol period).

The identity tensor of order *N* and dimensions *R*, denoted as $I_{N,R}=\left[\delta_{r_{1},\cdots ,r_{N}}\right]$, with $r_{n}\in \langle R\rangle$ for n∈〈N〉, is a diagonal tensor whose diagonal elements are equal to 1 and other elements to 0. The generalized Kronecker delta is defined as follows:

$$\delta_{r_{1},\cdots ,r_{N}}=\left\{\begin{array}{c} 1\;\mathrm{if}\;r_{1}=\cdots =r_{N}\\ 0\;\mathrm{otherwise} \end{array}\right..$$

Table 2 summarizes the notation used for sets of indices and dimensions [33].

### 2.1. Index Convention

We now introduce the index convention, which allows for eliminating the summation symbols in formulae involving multi-index variables. For example, $\sum_{i=1}^{I}a_{i}b_{i}$ is simply written as $a_{i}b_{i}$. Note that there are two differences relative to Einstein’s summation convention:

- Each index can be repeated more than twice in an expression;
- Ordered index sets are allowed.

For example, the index convention allows multiple sums to be abbreviated as follows:

(1) $$\begin{array}{cc} \sum_{i_{1}=1}^{I_{1}}\cdots \sum_{i_{P}=1}^{I_{P}}x_{i_{1},\cdots ,i_{P}}y_{i_{1},\cdots ,i_{P}}= & \sum_{{\underline{i}}_{P}=\underline{1}}^{{\underline{I}}_{P}}x_{{\underline{i}}_{P}}y_{{\underline{i}}_{P}}=x_{{\underline{i}}_{P}}y_{{\underline{i}}_{P}} \end{array}$$

(2) $$\begin{array}{cc} \sum_{i_{1}=1}^{I_{1}}\cdots \sum_{i_{P}=1}^{I_{P}}x_{i_{1},\cdots ,i_{P}}\prod_{p=1}^{P}a_{i_{p}}^{\left(p\right)}= & \sum_{{\underline{i}}_{P}=\underline{1}}^{{\underline{I}}_{P}}x_{{\underline{i}}_{P}}\prod_{p=1}^{P}a_{i_{p}}^{\left(p\right)}=\prod_{p=1}^{P}a_{i_{p}}^{\left(p\right)}x_{{\underline{i}}_{P}}, \end{array}$$

where $\underline{1}$ denotes a set of ones whose number is fixed by the index *P* of the set ${\underline{I}}_{P}$. The notation ${\underline{i}}_{P}$ and ${\underline{I}}_{P}$ allows us to simplify the expression of the multiple sums into a single sum over an index set, which is further simplified using the index convention.

The index convention can be interpreted in terms of two types of summation, one associated with row indices (subscripts), and one associated with column indices (superscripts), with the following rules [33,46]:

- The order of the column indices is independent of the order of the row indices;
- Consecutive row and column indices (or index sets) can be permuted.

In Table 3, we present examples of vector and matrix Kronecker products and a matrix product using index convention, where $e_{ij}≜e_{i}^{\left(I\right)}⊗e_{j}^{\left(J\right)}$, and $e_{i}^{j}≜e_{i}^{\left(I\right)}⊗{\left(e_{j}^{\left(J\right)}\right)}^{T}$.

### 2.2. Notion of Slice

If we fix N−1 indices of an *N*th-order tensor, $X\in K^{{\underline{I}}_{N}}$, we obtain a vector slice, called a fiber, and for N−2 fixed indices, we have a matrix slice. In Table 4, we define three types of vector and matrix slices for a third-order tensor, $X\in K^{I\times J\times K}$.

Similarly, we can define vector, matrix, and third-order tensor slices for a fourth-order tensor, $X\in K^{I\times J\times K\times L}$, such as $x_{•,j,k,l}\in K^{I}\;\;,\;\;X_{•,•,k,l}\in K^{I\times J}\;\;,\;\;X_{•,•,•,l}\in K^{I\times J\times K}$.

### 2.3. Matrix Unfoldings of a Tensor

A general matrix unfolding formula for an *N*th-order tensor, $X\in K^{{\underline{I}}_{N}}$, is given by [38]:

(3) $$X_{S_{1};\;S_{2}}=\sum_{i_{1}=1}^{I_{1}}\cdots \sum_{i_{N}=1}^{I_{N}}x_{i_{1},\cdots ,i_{N}}\left(\underset{n\in S_{1}}{⊗}e_{i_{n}}^{\left(I_{n}\right)}\right){\left(\underset{n\in S_{2}}{⊗}e_{i_{n}}^{\left(I_{n}\right)}\right)}^{T}\in K^{J_{1}\times J_{2}},$$

where $S_{1}$ and $S_{2}$ are two disjoint ordered subsets of the set of modes S=〈N〉, composed of *p* and N−p modes, respectively, with p∈〈N−1〉, and $J_{n_{1}}=\underset{n\in S_{n_{1}}}{\prod I_{n}}$, for $n_{1}=1\;\mathrm{and}\;\;2$. The subsets $S_{1}$ and $S_{2}$ contain the modes associated with the rows and the columns of the matrix unfolding $X_{S_{1}\;;\;S_{2}}$ of X, respectively.

Such a matrix unfolding results from two mode combinations associated with the sets $S_{1}$ and $S_{2}$, using the convention that the order of the dimensions in a product, $\prod_{p=1}^{P}I_{p}≜I_{1}\cdots I_{P}$, corresponding to a combination of *P* modes, follows the order of variation of the indices, with $i_{1}$ varying more slowly than $i_{2}$, which, in turn, varies more slowly than $i_{3}$, etc.

For example, in the flat mode-1 unfolding $X_{I\times KJ}$ of the third-order tensor $X\in K^{I\times J\times K}$, index *k* varies more slowly than *j*, which implies $x_{ijk}={\left[X_{I\times KJ}\right]}_{i,(k-1)J+j}$. Similarly, we have $x_{ijk}={\left[X_{J\times IK}\right]}_{j,(i-1)K+k}={\left[X_{K\times JI}\right]}_{k,(j-1)I+i}$.

Transposing $X_{I\times KJ}$ achieves the tall mode-1 unfolding $X_{KJ\times I}={\left[X_{I\times KJ}\right]}^{T}$. For a third-order tensor, in addition to $X_{KJ\times I}$, there are five other tall matrix unfoldings, denoted as $X_{JK\times I},X_{KI\times J},X_{IK\times J},X_{IJ\times K},X_{JI\times K}$.

### 2.4. Basic Tensor Operations

In Table 5, we recall the definitions of some basic operations: the outer product of vectors, the mode-*p* (multiple-mode-*p*) product of a tensor with a matrix (*P* matrices), and the modes-(p,n) product of two tensors, respectively denoted as ∘, $\times_{p}$, $\overset{P}{\underset{p=1}{\times }}$ and $\times_{p}^{n}$.

Note that the outer product of *P* non-zero vectors $u^{\left(p\right)}\in K^{I_{p}},p\in \langle P\rangle$, gives a rank-one *P*th-order tensor of size ${\underline{I}}_{P}$.

The mode-*p* product of a tensor $X\in K^{{\underline{I}}_{P}}$ with matrices $A\in K^{J_{p}\times I_{p}},B\in K^{K_{p}\times J_{p}}$ and $A^{\left(p\right)}\in K^{J_{p}\times I_{p}}$ for p∈〈P〉 satisfies the following properties:

(4) $$\begin{array}{cc} X\times_{p}A\times_{p}B= & \;X\times_{p}\left(BA\right) \end{array}$$

(5) $$\begin{array}{cc} X\overset{P}{\underset{p=1}{\times }}A^{\left(\pi (p)\right)}= & \;X\overset{P}{\underset{p=1}{\times }}A^{\left(p\right)}. \end{array}$$

The last equality means that, for any permutation, π(.), of the indices p∈〈P〉, the order of the mode-*p* products is irrelevant when the indices are all distinct.

The modes-(p,n) product of the tensors $X\in K^{{\underline{I}}_{P}}$ and $Y\in K^{{\underline{J}}_{N}}$, with $I_{p}=J_{n}=K$, corresponds to a contraction along the modes *p* of X and *n* of Y.

The contracted product $\times_{p}^{n}$ is associative; i.e., for any tensors $A\in K^{{\underline{I}}_{P}}$, $B\in K^{{\underline{J}}_{N}}$ and $C\in K^{{\underline{K}}_{Q}}$, such that $I_{p}=J_{n}$ and $J_{m}=K_{q}$, with m≠n, we have the following:

(6) $$\left(A\times_{p}^{n}B\right)\times_{m}^{q}C=A\times_{p}^{n}\left(B\times_{m}^{q}C\right)=A\times_{p}^{n}B\times_{m}^{q}C.$$

This doubly contracted product yields a tensor of order P+N+Q−4.

When the indices (m,n,p,q) represent numbers of modes in the tensors, the property (6) is no longer valid because the result of the double product $\times_{p}^{n}$ and $\times_{m}^{q}$ depends on the order in which these products are calculated.

For instance, for $A\in K^{I_{1}\times J_{1}\times R_{1}},B\in K^{R_{1}\times I_{2}\times J_{2}\times R_{2}}$ and $C\in K^{R_{2}\times I_{3}\times J_{3}}$, the double contracted product can be written in two different ways:

(7) $$\left(A\times_{3}^{1}B\right)\times_{5}^{1}C=A\times_{3}^{1}\left(B\times_{4}^{1}C\right)=A\times_{3}^{1}B\times_{4}^{1}C\in K^{I_{1}\times J_{1}\times I_{2}\times J_{2}\times I_{3}\times J_{3}}.$$

On the left-hand side of this double equality, the product $\times_{3}^{1}$ is performed first, and then, it is followed by the product $\times_{5}^{1}$. Meanwhile, in the second term of the equality, the product $\times_{4}^{1}$ is first calculated, followed by the product $\times_{3}^{1}$. The last term in the equality corresponds to two-by-two contractions of adjacent blocks. The first writing will be used to represent the TTD model by means of Equation (12) with contraction operations performed from left to right, while the third writing will be used in Equation (13).

### 2.5. Inner Product and Frobenius Norm

In the case of two complex-valued tensors, $A,B\in C^{{\underline{I}}_{N}}$, of order *N* and the same size, their Hermitian inner product is given by the following:

(8) $$\langle A,B\rangle =\sum_{i_{1}=1}^{I_{1}}\cdots \sum_{i_{N}=1}^{I_{N}}a_{i_{1},\cdots ,i_{N}}b_{i_{1},\cdots ,i_{N}}^{*}=\overset{{\underline{I}}_{N}}{\sum_{{\underline{i}}_{N}=\underline{1}}}a_{{\underline{i}}_{N}}b_{{\underline{i}}_{N}}^{*}.$$

We can also write it using the Hermitian inner product of vectorized forms of A and B:

(9) $$\langle A,B\rangle ={\mathrm{vec}}^{H}\left(B\right)\;\mathrm{vec}\left(A\right),$$

where vec(A) and vec(B) are vectorizations associated with the same mode combination of A and B.

The Frobenius norm of $A\in K^{{\underline{I}}_{N}}$ is the square root of the inner product of the tensor with itself; i.e.,

(10) $${∥A∥}_{F}={\langle A,A\rangle }^{1/2}=\sqrt{\sum_{i_{1}=1}^{I_{1}}\cdots \sum_{i_{N}=1}^{I_{N}}{\left|a_{i_{1},\cdots ,i_{N}}\right|}^{2}},$$

where |·| represents the absolute value or the modulus, depending on whether A is real (K=R) or complex (K=C).

Since the Frobenius norm is equal to the square root of the sum of the squares of the absolute value or modulus of all elements of the tensor, it is also given by the following:

(11) $${∥A∥}_{F}={∥\mathrm{vec}\left(A\right)∥}_{2}={∥A_{S_{1};S_{2}}∥}_{F},$$

that means the Euclidean (if K=R) or Hermitian (if K=C) norm of one of its vectorized forms, or as the Frobenius norm of one of its matrix unfoldings $A_{S_{1};S_{2}}$, defined in (3).

## 3. Overview of Tensor Models

**Figure 2:**
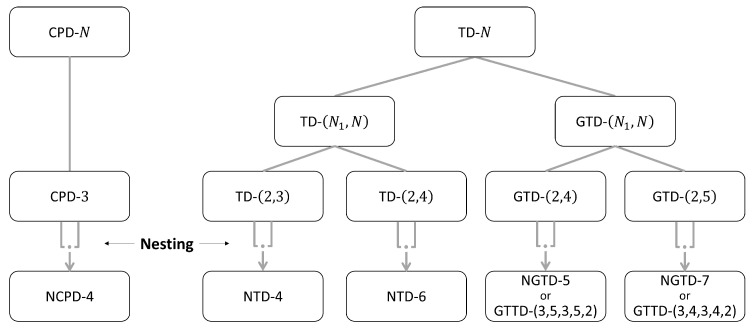
Nested tensor decompositions based on TD and CPD.

In this section, we provide an overview of the tensor models that will be used in Section 5 when designing two-hop relay systems. We first present the standard Tucker decomposition (TD), the canonical polyadic decomposition (CPD), also known as PARAFAC [8] or CANDECOMP [47], with two variants TD-$\left(N_{1},N\right)$ and GTD-$\left(N_{1},N\right)$, and the tensor train decomposition (TTD), with a new generalization that we will call generalized TTD (GTTD). Then, two basic nested decompositions, the so-called NCPD-4 and NTD-4 models, are described in detail. Three new nested models, called NTD-6, NGTD-5 and NGTD-7, are introduced for the first time. Figure 2 shows how the considered nested models are constructed by nesting two basic models: CPD-3 for NCPD-4; TD-(2,3) and TD-(2,4) for NTD-4 and NTD-6, respectively; and GTD-(2,4) and GTD-(2,5) for NGTD-5 and NGTD-7. For the NTD and GNTD models, an interpretation in terms of TTD and GTTD is highlighted.

### 3.1. TD and CPD Models

In Table 6 and Table 7, we first present various representations of the TD and CPD of a tensor of orders *N* and three: scalar writing, writings with mode-*n* products and outer products, and matrix unfoldings.

Comparing Table 7 with Table 6, we can conclude that a CPD can be viewed as a particular TD with an identity core tensor $I_{N,R}$, implying $R_{n}=R$ for n∈〈N〉 in the case of an *N*th-order tensor and P=Q=S=R for a third-order tensor. This results in the sum of *R* rank-one tensors. When *R* is minimal, it is called the tensor rank or the canonical rank.

The TD and CPD models will be abbreviated as $〚G;A^{\left(1\right)},\cdots ,A^{\left(N\right)};R_{1},\cdots ,R_{N}〛$ and $〚A^{\left(1\right)},\cdots ,A^{\left(N\right)};R〛$, respectively, for an *N*th-order tensor $X\in K^{{\underline{I}}_{N}}$ and 〚G;A,B,C;P,Q,S〛 and 〚A,B,C;R〛, respectively, for a third-order tensor $X\in K^{I\times J\times K}$.

When $R_{n},n\in \langle N\rangle$ and P,Q and *S* are minimal, the *N*-uplet $\left(R_{1},\cdots ,R_{N}\right)$ and the triplet (P,Q,S) are called the multilinear and trilinear ranks, respectively. There is a stable (non-iterative) algorithm, called higher-order singular value decomposition (HOSVD), for estimating multilinear rank and matrix factors in an orthogonal format, i.e., an orthogonal Tucker decomposition [48]. This algorithm is very often used to calculate a rank-one approximation of a tensor. A truncated version, called THOSVD, is also very useful for calculating a low multilinear rank approximation.

A variant of the TD model, called the Tucker-$\left(N_{1},N\right)$ decomposition and abbreviated as TD-$\left(N_{1},N\right)$ [36], is presented in Table 8, with special cases corresponding to the Tucker-(2,3) and Tucker-(1,3) models, also denoted as Tucker-2 and Tucker-1 in the literature. Note that, in this decomposition, $N-N_{1}$ factor matrices are identity matrices chosen such that $A^{\left(n\right)}=I_{I_{n}}$, which implies $R_{n}=I_{n}$, for $n=N_{1}+1,\cdots ,N$, and hence, $G\in K^{R_{1}\times \cdots \times R_{N_{1}}\times I_{N_{1}+1}\times \cdots \times I_{N}}$.

In Table 9, we illustrate the generalized Tucker-$\left(N_{1},N\right)$ decomposition, denoted as GTD-$\left(N_{1},N\right)$, corresponding to a Tucker-$\left(N_{1},N\right)$ one when some of the factors are tensors instead of matrices, first introduced in [38] to model a wireless communication system using tensor space-time-frequency (TSTF) coding. The case of a fourth-order tensor $X\in K^{{\underline{I}}_{4}}$ is considered, with $\left(N_{1},N\right)=\left(2,4\right)$, a fourth-order core tensor $G\in K^{R_{1}\times R_{2}\times I_{3}\times I_{4}}$, a third-order tensor factor $A\in K^{I_{1}\times R_{1}\times I_{3}}$, and a matrix factor $B\in K^{I_{2}\times R_{2}}$.

### 3.2. TTD Models

The tensor train (TT) format was proposed in [44] as a reduced parametric complexity tensor representation in order to alleviate the curse of dimensionality with standard tensor decompositions due to an exponential growth of their number of parameters when the order of the tensor increases. This format is very useful for representing high-order data tensors.

**Figure 3:**
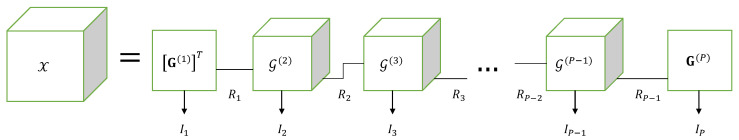
TTD of a *P*th-order tensor, $X\in K^{{\underline{I}}_{P}}$.

A TTD model of an *P*th-order tensor $X\in K^{{\underline{I}}_{P}}$ consists of decomposing it into a train of two matrices, $G^{\left(1\right)}$ and $G^{\left(P\right)}$, and (P−2) third-order tensors $G^{\left(p\right)},p\in \left\{2,\cdots ,P-1\right\}$, with the following dimensions:

$$G^{\left(1\right)}\in K^{I_{1}\times R_{1}}\;\;\;;\;\;\;G^{\left(P\right)}\in K^{R_{P-1}\times I_{P}}\;\;\;;\;\;\;G^{\left(p\right)}\in K^{R_{p-1}\times I_{p}\times R_{p}},$$

as illustrated in Figure 3.

The TTD can be written as a sequence, from left to right, of (P−1) contractions between the factors $G^{\left(p\right)}$, for p∈{2,⋯,P−1}, and $G^{\left(P\right)}$ with the tensor resulting from all contractions to the left of each factor, as follows:

(12) $$X=\left(\left(\cdots \left(\left(G^{\left(1\right)}\times_{2}^{1}G^{\left(2\right)}\right)\times_{3}^{1}G^{\left(3\right)}\right)\times_{4}^{1}\cdots \right)\times_{P-1}^{1}G^{\left(P-1\right)}\right)\times_{P}^{1}G^{\left(P\right)},$$

with the modes-(1,p) products calculated from p=2 to p=P.

Considering two-by-two contractions of adjacent blocks, the TTD can be written as follows:

(13) $$X=G^{\left(1\right)}\times_{2}^{1}G^{\left(2\right)}\times_{3}^{1}G^{\left(3\right)}\times_{3}^{1}G^{\left(4\right)}\times_{3}^{1}\cdots \times_{3}^{1}G^{\left(P-1\right)}\times_{3}^{1}G^{\left(P\right)},$$

where the products $\times_{3}^{1}$ correspond to separate contractions of two adjacent tensors, $G^{\left(p\right)}$ and $G^{\left(p+1\right)}$, along their common mode associated with the index $r_{p}$ for p∈{2,⋯P−1}.

The factors $G^{\left(p\right)},p\in \langle P\rangle$ are called TT cores, and the integer numbers $R_{p},p\in \langle P-1\rangle$ are the TT-ranks. For p=1 and p=P, the TT-cores are two matrices, which implies $R_{0}=R_{P}=1$. The TT-rank $R_{p}$ is given by the rank of the matrix unfolding $X_{I_{1}\cdots I_{p}\times I_{p+1}\cdots I_{P}}$.

The TTD can also be concisely written in a scalar way as follows:

(14) $$x_{{\underline{i}}_{P}}=\sum_{r_{1}=1}^{R_{1}}\cdots \sum_{r_{P-1}=1}^{R_{P-1}}g_{i_{1},r_{1}}^{\left(1\right)}g_{r_{1},i_{2},r_{2}}^{\left(2\right)}g_{r_{2},i_{3},r_{3}}^{\left(3\right)}\cdots g_{r_{P-2},i_{P-1},r_{P-1}}^{\left(P-1\right)}g_{r_{P-1},i_{P}}^{\left(P\right)},$$

or using row vector–matrix, matrix–matrix, and matrix–column vector products as follows:

(15) $$x_{{\underline{i}}_{P}}=g_{i_{1},•}^{\left(1\right)}\;G_{•,i_{2},•}^{\left(2\right)}G_{•,i_{3},•}^{\left(3\right)}\cdots G_{•,i_{P-1},•}^{\left(P-1\right)}\;g_{•,i_{P}}^{\left(P\right)},$$

where $g_{i_{1},•}^{\left(1\right)}\in K^{1\times R_{1}}$ and $g_{•,i_{P}}^{\left(P\right)}\in K^{R_{P-1}}$ are the $i_{1}$th row vector of $G^{\left(1\right)}$, and the $i_{P}$th column-vector of $G^{\left(P\right)}$, respectively, whereas $G_{•,i_{p},•}^{\left(p\right)}\in K^{R_{p-1}\times R_{p}}$ is the $i_{p}$th lateral slice of $G^{\left(p\right)}$, for p∈{2,⋯,P−1}, i.e.,

$$\begin{array}{cc} g_{i_{1},•}^{\left(1\right)}= & \;\left[g_{i_{1},1}^{\left(1\right)}\cdots g_{i_{1},R_{1}}^{\left(1\right)}\right]\;\;\;,\;\;\;g_{•,i_{P}}^{\left(P\right)}={\left[g_{1,i_{P}}^{\left(P\right)}\cdots g_{R_{P-1},i_{P}}^{\left(P\right)}\right]}^{T}\\ G_{•,i_{p},•}^{\left(p\right)}= & \left[\begin{array}{ccc} g_{1,i_{p},1}^{\left(p\right)} & \cdots & g_{1,i_{p},R_{p}}^{\left(p\right)}\\ \vdots & \; & \vdots \\ g_{R_{p-1},i_{p},1}^{\left(p\right)} & \cdots & g_{R_{p-1},i_{p},R_{p}}^{\left(p\right)} \end{array}\right]. \end{array}$$

From Equation (15), we can conclude that TTD is not unique since it is invariant for all non-singular transformation matrices, $T^{\left(p\right)}\in K^{R_{p}\times R_{p}}$, which transform the lateral slice $G_{•,i_{p},•}^{\left(p\right)}$ of the core $G^{\left(p\right)}$, for p∈{2,⋯,P−1}, in such a way that the following applies:

(16) $$F_{•,i_{p},•}^{\left(p\right)}={\left[T^{\left(p-1\right)}\right]}^{-1}G_{•,i_{p},•}^{\left(p\right)}T^{\left(p\right)},$$

and, for p=1 and p=P, $F^{\left(1\right)}=G^{\left(1\right)}T^{\left(1\right)}$ and $F^{\left(P\right)}={\left[T^{\left(P-1\right)}\right]}^{-1}G^{\left(P\right)}$. Indeed, it is easy to verify that:

(17) $$f_{i_{1},•}^{\left(1\right)}\;F_{•,i_{2},•}^{\left(2\right)}F_{•,i_{3},•}^{\left(3\right)}\cdots F_{•,i_{P-1},•}^{\left(P-1\right)}\;f_{•,i_{P}}^{\left(P\right)}=g_{i_{1},•}^{\left(1\right)}\;G_{•,i_{2},•}^{\left(2\right)}G_{•,i_{3},•}^{\left(3\right)}\cdots G_{•,i_{P-1},•}^{\left(P-1\right)}\;g_{•,i_{P}}^{\left(P\right)}=x_{{\underline{i}}_{P}}.$$

The TTD can also be written using outer products of column vectors as:

(18) $$X=\sum_{r_{1}=1}^{R_{1}}\cdots \sum_{r_{P-1}=1}^{R_{P-1}}g_{•,r_{1}}^{\left(1\right)}\circ g_{r_{1},•,r_{2}}^{\left(2\right)}\cdots \circ g_{r_{P-2},•,r_{P-1}}^{\left(P-1\right)}\circ {\left[g_{r_{P-1},•}^{\left(P\right)}\right]}^{T},$$

with $g_{•,r_{1}}^{\left(1\right)}\in K^{I_{1}}$, $g_{r_{P-1},•}^{\left(P\right)}\in K^{1\times I_{P}}$, and $g_{r_{p-1},•,r_{p}}^{\left(p\right)}\in K^{I_{p}}$, for p∈{2,⋯,P−1}. In Table 10, we summarize the different writings of a TTD, with $r_{0}=r_{P}=1$.

Note that, for the TTD model, each carriage is characterized by only one inner index, $i_{p}$. The outer indices, $r_{p}$, are associated with modes common to two consecutive wagons. They represent the modes on which the tensor contractions operate.

**Figure 4:**
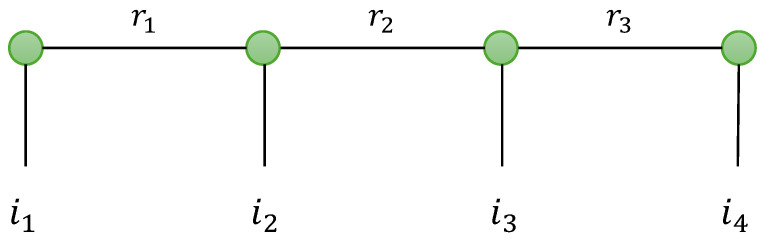
Graph of the TTD-4 model for a fourth-order tensor $X\in K^{{\underline{I}}_{4}}$.

The TTD model can be represented using a graph, as shown in Figure 4, for a fourth-order tensor, $X\in K^{{\underline{I}}_{4}}$. The nodes, denoted as (•), represent matrices or third-order tensors in the train, and the edges are labeled by an index $i_{p}$ of X, or an index $r_{p}$ relative to the TT rank $R_{p}$. The number of edges associated with a node is equal to two or three, depending on whether the node is a matrix or a third-order tensor, respectively. The number of inner indices $i_{p}$ is equal to the order of X.

TTD combines the advantages of CPD and TD in terms of the following: (i) parametric complexity, which is proportional to the tensor order *P* as for CPD, while it increases exponentially with the order *P* for TD, due to the dimensionality of the core tensor; and (ii) the existence of a stable parametric estimation algorithm, based on SVD calculations, as for TD.

In Table 11, we summarize the notations and the element $x_{{\underline{i}}_{N}}$ for the CPD, TD, and TTD models, and their parametric complexity is compared in terms of the size of matrix and tensor factors, assuming $I_{n}=I$ and $R_{n}=R$ for all n∈〈N〉.

**Figure 5:**
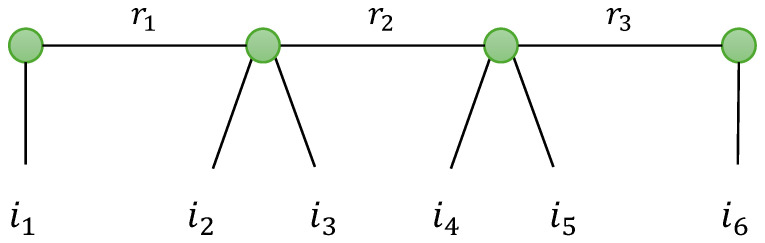
Graph of the GTTD-(2,4,4,2) model for a sixth-order tensor $X\in K^{{\underline{I}}_{6}}$.

A generalization of TTD is now introduced in defining a train of tensors of any order. For instance, a tensor $X\in K^{{\underline{I}}_{6}}$ can be decomposed into a train of two matrices and two fourth-order tensors, as illustrated with the graph in Figure 5. Such a generalized TTD will be denoted as GTTD-(2,4,4,2), where each number is associated with the order of the corresponding tensor in the train. This type of generalization will be encountered for modeling relay systems based on tensor codings. See Section 5.3.

### 3.3. Nested Tensor Models

We now present two families of nested tensor models, namely NCPD and NTD. In Section 3.3.1 and Section 3.3.2, the standard NCPD-4 and NTD-4 models are described for a fourth-order tensor $X\in K^{{\underline{I}}_{4}}$. Then, in Section 3.3.3, two generalizations corresponding to NTD-6 and NGTD-7 models are introduced for sixth- and seventh-order tensors, respectively.

The NCPD-4 and NTD-4 models, summarized in Table 12, will be abbreviated as follows:

$$〚A^{\left(1\right)},B^{\left(1\right)},G,A^{\left(2\right)},B^{\left(2\right)};R_{1},R_{2}〛\;\mathrm{and}\;〚A^{\left(1\right)},G^{\left(1\right)},U,G^{\left(2\right)},A^{\left(2\right)};R_{1},R_{2},R_{3},R_{4}〛,$$

where, by analogy with the TT ranks, the integers $\left(R_{1},R_{2}\right)$ and $\left(R_{1},R_{2},R_{3},R_{4}\right)$ will be called NCPD and NTD ranks, respectively. The TTD-4 model is also presented in Table 12.

The NCPD-4 and NTD-4 models were first highlighted in [39,41], respectively, in the context of a point-to-point communication system using a DKRSTF coding and a two-hop relay system using a TST coding.

#### 3.3.1. NCPD-4 Model

The NCPD-4 model for $X\in K^{{\underline{I}}_{4}}$ can be interpreted as the nesting of two CPD-3 models of the following third-order tensors $X^{\left(1\right)}\in K^{I_{1}\times I_{2}\times R_{2}}$ and $X^{\left(2\right)}\in K^{R_{1}\times I_{3}\times I_{4}}$:

(19) $$\begin{array}{cc} X^{\left(1\right)}= & \;I_{R_{1}}\times_{1}A^{\left(1\right)}\times_{2}B^{\left(1\right)}\times_{3}G^{T} \end{array}$$

(20) $$\begin{array}{cc} X^{\left(2\right)}= & \;I_{R_{2}}\times_{1}G\times_{2}A^{\left(2\right)}\times_{3}B^{\left(2\right)}, \end{array}$$

that share the common matrix factor G, as shown in the following equation:

(21) $$x_{{\underline{i}}_{4}}=x_{i_{1},\cdots ,i_{4}}=\sum_{r_{1}=1}^{R_{1}}\sum_{r_{2}=1}^{R_{2}}a_{i_{1},r_{1}}^{\left(1\right)}b_{i_{2},r_{1}}^{\left(1\right)}g_{r_{1},r_{2}}a_{i_{3},r_{2}}^{\left(2\right)}b_{i_{4},r_{2}}^{\left(2\right)},$$

where the red color is associated with the sum over index $r_{1}$ in the CPD model of the tensor $X^{\left(1\right)}$, while the blue color is used for the sum over index $r_{2}$ in the CPD model of the tensor $X^{\left(2\right)}$, and the common factor of these two CPDs is in green.

**Figure 6:**
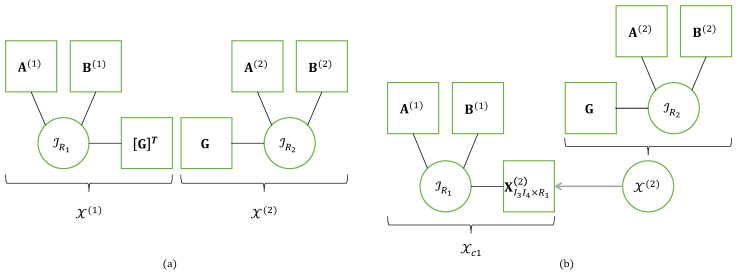
NCPD-4 model as (**a**) a nesting of two CPD-3 models and (**b**) a cascade of two CPD-3 models.

This nesting of the CPD-3 models (19) and (20), represented in Figure 6a, is characterized by five matrix factors $\left(A^{\left(1\right)},B^{\left(1\right)},G,A^{\left(2\right)},B^{\left(2\right)}\right)$.

According to the definition of the CPD-3 models (19) and (20), we have the following:

(22) $$\begin{array}{cc} x_{i_{1},i_{2},r_{2}}^{\left(1\right)}= & \;\sum_{r_{1}=1}^{R_{1}}a_{i_{1},r_{1}}^{\left(1\right)}b_{i_{2},r_{1}}^{\left(1\right)}g_{r_{1},r_{2}} \end{array}$$

(23) $$\begin{array}{cc} x_{r_{1},i_{3},i_{4}}^{\left(2\right)}= & \;\sum_{r_{2}=1}^{R_{2}}g_{r_{1},r_{2}}a_{i_{3},r_{2}}^{\left(2\right)}b_{i_{4},r_{2}}^{\left(2\right)}. \end{array}$$

Equation (21) can then be rewritten as follows:

(24) $$x_{{\underline{i}}_{4}}=\sum_{r_{1}=1}^{R_{1}}a_{i_{1},r_{1}}^{\left(1\right)}b_{i_{2},r_{1}}^{\left(1\right)}x_{r_{1},i_{3},i_{4}}^{\left(2\right)}=\sum_{r_{2}=1}^{R_{2}}x_{i_{1},i_{2},r_{2}}^{\left(1\right)}a_{i_{3},r_{2}}^{\left(2\right)}b_{i_{4},r_{2}}^{\left(2\right)}.$$

Let us define the matrix unfoldings $X_{I_{1}I_{2}\times R_{2}}^{\left(1\right)}$ and $X_{I_{3}I_{4}\times R_{1}}^{\left(2\right)}$, which result from a combination of the first two modes of $X^{\left(1\right)}$ and the last two modes of $X^{\left(2\right)}$, respectively. The writing (24) of $x_{{\underline{i}}_{4}}$ can be interpreted as two CPD-3 models associated with the following third-order contracted forms $X_{c_{1}}\in K^{I_{1}\times I_{2}\times I_{3}I_{4}}$ and $X_{c_{2}}\in K^{I_{1}I_{2}\times I_{3}\times I_{4}}$ of the fourth-order tensor X:

(25) $$\begin{array}{cc} X_{c_{1}}= & \;I_{R_{1}}\times_{1}A^{\left(1\right)}\times_{2}B^{\left(1\right)}\times_{3}X_{I_{3}I_{4}\times R_{1}}^{\left(2\right)} \end{array}$$

(26) $$\begin{array}{cc} X_{c_{2}}= & \;I_{R_{2}}\times_{1}X_{I_{1}I_{2}\times R_{2}}^{\left(1\right)}\times_{2}A^{\left(2\right)}\times_{3}B^{\left(2\right)}. \end{array}$$

Figure 6b illustrates another interpretation of the NCPD-4 model, deduced from the contracted form (25), as a cascade of two CPD-3 models. A similar interpretation in terms of cascade can be deduced from the contracted form (26).

Matrix unfoldings associated with the CPD models of the third-order tensors $X^{\left(1\right)}$, $X^{\left(2\right)}$, $X_{c_{1}}$ and $X_{c_{2}}$, defined in Equations (19), (20), (25) and (26), are deduced from the general formulae recalled in Table 7, as summarized in Table 13. Combining these matrix unfoldings leads to matrix unfoldings for the NCPD-4 model, as given in Table 14.

Applying the property $\mathrm{vec}\left(ABC^{T}\right)=\left(C⊗A\right)\mathrm{vec}\left(B\right)$ to the last equation in Table 14 gives a vectorized form of the tensor X:

(27) $$x_{I_{3}I_{4}I_{1}I_{2}}=\mathrm{vec}\left(X_{I_{1}I_{2}\times I_{3}I_{4}}\right)=\left(\left(A^{\left(2\right)}⋄B^{\left(2\right)}\right)⊗\left(A^{\left(1\right)}⋄B^{\left(1\right)}\right)\right)\mathrm{vec}\left(G\right).$$

Note that the first four matrix unfoldings in Table 14 and the vectorized form (27) contain one isolated factor (to the right of the equations) from $\left(A^{\left(1\right)},B^{\left(1\right)},G,A^{\left(2\right)},B^{\left(2\right)}\right)$. These unfoldings will be used separately and iteratively to estimate the matrix factors of the NCPD-4 model, with a five-step alternating least squares (ALS) algorithm, as presented in Section 4.2.

When some factors are known, the ALS algorithm can be simplified. That is the case in the context of relay system using the SKRST coding, where the factors $\left(B^{\left(1\right)},A^{\left(2\right)}\right)$ represent coding matrices assumed to be known at the receiver. Then, the other three factors $\left(A^{\left(1\right)},G,B^{\left(2\right)}\right)$ representing the symbol and channel matrices can be estimated using a three-step ALS algorithm. See Equations (67), (68) and (70).

From the cascade structure shown in Figure 6b, and, therefore, from Equation (25), we conclude that the tensor X can be represented by means of two coupled CPD-3 models associated with the contracted form $X_{c_{1}}$ and the tensor $X^{\left(2\right)}$, i.e., Equations (25) and (20). Similarly, the coupled CPD-3 models of the contracted form $X_{c_{2}}$ and the tensor $X^{\left(1\right)}$, i.e., Equations (26) and (19), can be used to represent the tensor X.

When $\left(B^{\left(1\right)},A^{\left(2\right)}\right)$ are assumed to be known, each CPD-3 model of $X^{\left(1\right)},X^{\left(2\right)}$, $X_{c_{1}}$ and $X_{c_{2}}$ contains two unknown matrix factors that can be estimated using a closed-form algorithm based on the Khatri–Rao factorization (KRF) method, as first proposed in [49].

Exploiting each set of coupled CPD-3 models allows for the estimatation of the three unknown factors $\left(A^{\left(1\right)},G,B^{\left(2\right)}\right)$ by means of a two-step procedure based on KRF. The resulting two KRF-based closed-form algorithms, which use the unfoldings in green in Table 13, will be described in Section 4.2. See Table 18.

**Remark 1.**  *The essential uniqueness of a CPD model, i.e., uniqueness up to column permutation and scaling ambiguities in the factor matrices, has been the subject of numerous articles in the literature. From a modeling point of view, uniqueness means that the model $〚A\Pi \Lambda^{\left(A\right)},B\Pi \Lambda^{\left(B\right)},C\Pi \Lambda^{\left(C\right)};R\;〛$ is equivalent to the model 〚A,B,C;R〛, where ***Π*** is a permutation matrix, and $\left(\Lambda^{\left(A\right)},\Lambda^{\left(B\right)},\Lambda^{\left(C\right)}\right)$ are R×R diagonal matrices, such that their product is the identity matrix: $\Lambda^{\left(A\right)}\Lambda^{\left(B\right)}\Lambda^{\left(C\right)}$$=I_{R}$.*

*The following sufficient uniqueness condition has been established by Kruskal [50] for a third-order CPD model 〚A,B,C;R〛:*

(28) $$k_{A}+k_{B}+k_{C}\geq 2R+2,$$

*where $k_{A}$ denotes the k-rank (also known as the Kruskal’s rank) of A, i.e., the largest integer $k_{A}$ such that every set of $k_{A}$ columns of A is linearly independent.*

*When a factor matrix (e.g., C) is known, and the Kruskal condition (28) is satisfied, essential uniqueness is guaranteed without any permutation ambiguity and with only two diagonal ambiguity matrices $\left(\Lambda^{\left(A\right)},\Lambda^{\left(B)\right)}\right)$, such that $\Lambda^{\left(A\right)}\Lambda^{\left(B\right)}=I_{R}$.*

Applying Kruskal’s condition to the CPD models (19) and (20) leads to the following uniqueness conditions for the NCPD-4 model:

(29) $$\begin{array}{cc} k_{A^{\left(1\right)}}+k_{B^{\left(1\right)}}+k_{G^{T}}\geq & \;2R_{1}+2 \end{array}$$

(30) $$\begin{array}{cc} k_{A^{\left(2\right)}}+k_{B^{\left(2\right)}}+k_{G}\geq & \;2R_{2}+2. \end{array}$$

As mentioned previously, in the context of relay systems, the factors $\left(B^{\left(1\right)},A^{\left(2\right)}\right)$ will be assumed to be known and full column rank, implying $k_{B^{\left(1\right)}}=R_{1}$ and $k_{A^{\left(2\right)}}=R_{2}$. The Kruskal’s conditions (29) and (30) then become

(31) $$k_{A^{\left(1\right)}}+k_{G^{T}}\geq \;R_{1}+2\;\;\;\mathrm{and}\;\;\;k_{B^{\left(2\right)}}+k_{G}\geq \;R_{2}+2,$$

and the ambiguity relations for the NCPD-4 model (19) and (20) can be simplified as follows:

(32) $$\Lambda^{\left(A^{\left(1\right)}\right)}\Lambda^{\left(G^{T}\right)}=I_{R_{1}}\;\;\;\mathrm{and}\;\;\;\Lambda^{\left(B^{\left(2\right)}\right)}\Lambda^{\left(G\right)}=I_{R_{2}}.$$

#### 3.3.2. NTD-4 Model

In this section, we present the NTD-4 model for a fourth-order tensor, $X\in K^{{\underline{I}}_{4}}$. This model, introduced in Table 12, corresponds to the nesting of two Tucker-(2,3) decompositions of the third-order tensors $X^{\left(1\right)}\in K^{I_{1}\times I_{2}\times R_{3}}$ and $X^{\left(2\right)}\in K^{R_{2}\times I_{3}\times I_{4}}$:

(33) $$\begin{array}{cc} X^{\left(1\right)}= & \;G^{\left(1\right)}\times_{1}A^{\left(1\right)}\times_{2}I_{I_{2}}\times_{3}U^{T} \end{array}$$

(34) $$\begin{array}{cc} X^{\left(2\right)}= & \;G^{\left(2\right)}\times_{1}U\times_{2}I_{I_{3}}\times_{3}A^{\left(2\right)}, \end{array}$$

that share the matrix factor U, as illustrated in the following equation:

(35) $$x_{{\underline{i}}_{4}}=\sum_{r_{1}=1}^{R_{1}}\sum_{r_{2}=1}^{R_{2}}\sum_{r_{3}=1}^{R_{3}}\sum_{r_{4}=1}^{R_{4}}a_{i_{1},r_{1}}^{\left(1\right)}g_{r_{1},i_{2},r_{2}}^{\left(1\right)}u_{r_{2},r_{3}}g_{r_{3},i_{3},r_{4}}^{\left(2\right)}a_{i_{4},r_{4}}^{\left(2\right)}.$$

The red color is associated with the sums over indices $r_{1}$ and $r_{2}$ in the TD-(2,3) model of the tensor $X^{\left(1\right)}$, while the blue color is used for the sums over indices $r_{3}$ and $r_{4}$ in the TD-(2,3) model of the tensor $X^{\left(2\right)}$, and the common factor of these two TDs is in green.

Equation (35) is to be compared with Equation (21) of the NCPD-4 model. From this comparison, we deduce that the nesting of two CPD-3 models in the case of NCPD-4 is replaced with the nesting of two TD-(2,3) models for NTD-4.

**Figure 7:**
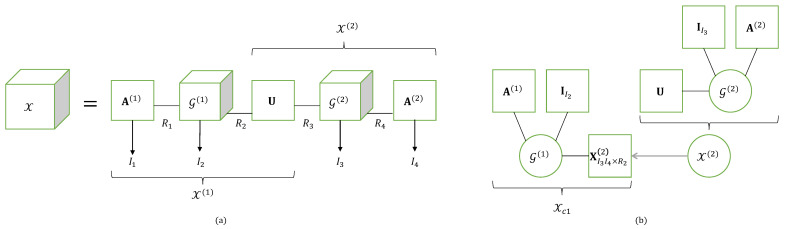
NTD-4 model as (**a**) a particular TTD and (**b**) a cascade of two TD-(2,3) models.

**Figure 8:**
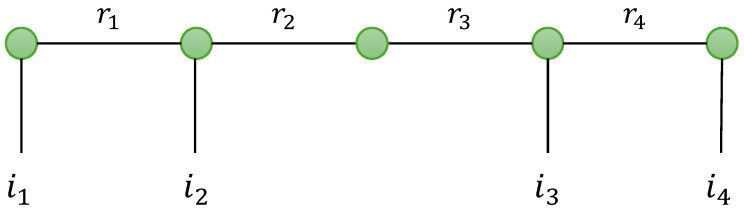
Graph of the NTD-4 model for a fourth-order tensor $X\in K^{{\underline{I}}_{4}}$.

The NTD-4 model can also be viewed as a particular TTD model, represented in Figure 7a and the graph in Figure 8. This TTD is composed of three matrix factors and two third-order core tensors: $\left(A^{\left(1\right)},G^{\left(1\right)},U,G^{\left(2\right)},A^{\left(2\right)}\right)$.

Following the same approach as for NCPD-4, the fourth-order tensor X can be written using the following two third-order contracted forms $X_{c_{1}}\in K^{I_{1}\times I_{2}\times I_{3}I_{4}}$ and $X_{c_{2}}\in K^{I_{1}I_{2}\times I_{3}\times I_{4}}$, which result from combinations of the last two modes of $X^{\left(2\right)}$ and the first two modes of $X^{\left(1\right)}$, respectively:

(36) $$\begin{array}{cc} X_{c_{1}}= & \;G^{\left(1\right)}\times_{1}A^{\left(1\right)}\times_{2}I_{I_{2}}\times_{3}X_{I_{3}I_{4}\times R_{2}}^{\left(2\right)} \end{array}$$

(37) $$\begin{array}{cc} X_{c_{2}}= & \;G^{\left(2\right)}\times_{1}X_{I_{1}I_{2}\times R_{3}}^{\left(1\right)}\times_{2}I_{I_{3}}\times_{3}A^{\left(2\right)}. \end{array}$$

These contracted decompositions are to be compared with Equations (25) and (26) for the NCPD-4 model where the core tensors $G^{\left(1\right)}$ and $G^{\left(2\right)}$ are replaced with the identity tensors $I_{R_{1}}$ and $I_{R_{2}}$, respectively, and the identity matrices $\left(I_{I_{2}},I_{I_{3}}\right)$ are replaced with the matrix factors $\left(B^{\left(1\right)},A^{\left(2\right)}\right)$. From Equation (36), we deduce that the NTD-4 model can be viewed as the cascade of two TD-(2,3) models, as illustrated in Figure 7b. A similar cascade structure can be deduced from Equation (37).

Matrix unfoldings associated with the TD models of the third-order tensors $X^{\left(1\right)},X^{\left(2\right)}$, $X_{c_{1}}$ and $X_{c_{2}}$, defined in Equations (33)–(37), are obtained using the general formulae recalled in Table 6, as summarized in Table 15.

Vectorizing $X_{I_{1}I_{2}\times I_{3}I_{4}}$ yields the following vectorized form of X:

(38) $$x_{I_{3}I_{4}I_{1}I_{2}}=\mathrm{vec}\left(X_{I_{1}I_{2}\times I_{3}I_{4}}\right)=\left[\left(I_{I_{3}}⊗A^{\left(2\right)}\right)G_{I_{3}R_{4}\times R_{3}}^{\left(2\right)}⊗\left(A^{\left(1\right)}⊗I_{I_{2}}\right)G_{R_{1}I_{2}\times R_{2}}^{\left(1\right)}\right]\mathrm{vec}\left(U\right).$$

By combining the unfoldings in Table 15, it is easy to obtain the unfoldings of the NTD-4 model summarized in Table 16, where the last equation is the vectorized form (38).

**Remark 2.**  *We can make the following remarks:*

- *The equations in Table 16 can be used to build a five-step ALS-based algorithm to separately and iteratively estimate the factors $\left(A^{\left(1\right)},G^{\left(1\right)},A^{\left(2\right)},G^{\left(2\right)},U\right)$ of the NTD-4 model.*
- *When the tensors $G^{\left(1\right)}$ and $G^{\left(2\right)}$ are known, the equations in Table 16 can be used to estimate the unknown matrix factors $\left(A^{\left(1\right)},A^{\left(2\right)},U\right)$ of the NTD-4 model using a three-step ALS algorithm. In Section 4.3, equations in Table 15 will be exploited to derive two closed-form algorithms based on the Kronecker factorization (KronF) method. These algorithms will be used to develop semi-blind receivers for relay systems where the tensors $G^{\left(1\right)}$ and $G^{\left(2\right)}$ are STST coding tensors assumed to be known at the destination.*
- *Using the writing (15) of a TTD, the NTD-4 model (35) can be rewritten as follows:*
(39) $$x_{{\underline{i}}_{4}}=a_{i_{1},•}^{\left(1\right)}\;G_{•,i_{2},•}^{\left(1\right)}\;U\;G_{•,i_{3},•}^{\left(2\right)}\;a_{•,i_{4}}^{\left(2\right)},$$
  *which allows for the conclusion that the NTD-4 model is unique up to non-singular matrices $\Lambda^{\left(n\right)}\in K^{R_{n}\times R_{n}}$, n∈〈4〉, such that the following applies:*
(40) $$\begin{array}{cc} {\tilde{A}}^{\left(1\right)}= & \;A^{\left(1\right)}\Lambda^{\left(1\right)}\;\;,\;\;\tilde{U}={\left[\Lambda^{\left(2\right)}\right]}^{-1}U\Lambda^{\left(3\right)}\;\;,\;\;{\tilde{A}}^{\left(2\right)}={\left[\Lambda^{\left(4\right)}\right]}^{-1}A^{\left(2\right)} \end{array}$$
(41) $$\begin{array}{cc} {\tilde{G}}_{•,i_{2},•}^{\left(1\right)}= & \;{\left[\Lambda^{\left(1\right)}\right]}^{-1}G_{•,i_{2},•}^{\left(1\right)}\Lambda^{\left(2\right)}\;\;,\;\;{\tilde{G}}_{•,i_{3},•}^{\left(2\right)}={\left[\Lambda^{\left(3\right)}\right]}^{-1}G_{•,i_{3},•}^{\left(2\right)}\Lambda^{\left(4\right)}. \end{array}$$
  *Indeed, it is easy to verify that ${\tilde{a}}_{i_{1},•}^{\left(1\right)}\;{\tilde{G}}_{•,i_{2},•}^{\left(1\right)}\;\tilde{U}\;{\tilde{G}}_{•,i_{3},•}^{\left(2\right)}\;{\tilde{a}}_{•,i_{4}}^{\left(2\right)}=a_{i_{1},•}^{\left(1\right)}\;G_{•,i_{2},•}^{\left(1\right)}\;U\;G_{•,i_{3},•}^{\left(2\right)}\;a_{•,i_{4}}^{\left(2\right)}=x_{{\underline{i}}_{4}}$.*
- *When the core tensors $G^{\left(1\right)}$ and $G^{\left(2\right)}$ are perfectly known, as will be the case with the coding tensors in the context of relay systems, the ambiguity matrices $\Lambda^{\left(n\right)}$ become identity matrices multiplied by a scalar:*
(42) $$\Lambda^{\left(n\right)}=\alpha_{n}I_{R_{n}}\;\mathrm{for}\;n\in \langle 4\rangle ,\;\mathrm{with}\;\prod_{n=1}^{4}\alpha_{n}=1\;;\;\alpha_{2}=\alpha_{1}\;,\;\alpha_{3}=\alpha_{4}\;\mathrm{and}\;\tilde{U}=\frac{\alpha_{4}}{\alpha_{1}}U.$$
  *The scaling factors $\alpha_{1}$ and $\alpha_{4}$ can be determined using the knowledge of one element in $A^{\left(1\right)}$ and $A^{\left(4\right)}$, respectively.*

#### 3.3.3. NTD-6 and NGTD-7 Models

In Table 17, we present two generalizations of the NTD-4 model: the NTD-6 and NGTD-7 models, for tensors of order 6 and 7, respectively. These models will be used to represent relay systems with TST and TSTF codings, respectively, in Section 5.

**Figure 9:**
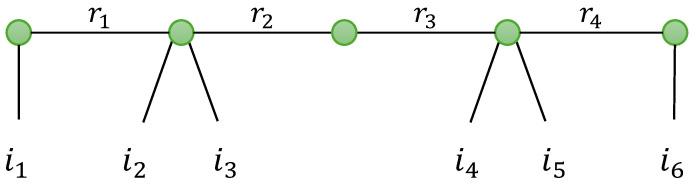
Graph of the NTD-6 model for a sixth-order tensor $X\in K^{{\underline{I}}_{6}}$.

The NTD-6 model corresponds to a GTTD-(2,4,2,4,2), which means a train of three matrix factors $\left(A^{\left(1\right)},U,A^{\left(2\right)}\right)$ and two fourth-order core tensors $\left(G^{\left(1\right)},G^{\left(2\right)}\right)$, illustrated in the graph in Figure 9, to be compared with Figure 8 for NTD-4. It can be viewed as the nesting of the following two TD-(2,4) models (see Table 8 with $\left(N_{1},N\right)=\left(2,4\right)$) of the fourth-order tensors $X^{\left(1\right)}\in K^{I_{1}\times I_{2}\times I_{3}\times R_{3}}$ and $X^{\left(2\right)}\in K^{R_{2}\times I_{4}\times I_{5}\times I_{6}}$, which share the matrix factor U:

(43) $$\begin{array}{cc} X^{\left(1\right)}= & \;G^{\left(1\right)}\times_{1}A^{\left(1\right)}\times_{4}U^{T} \end{array}$$

(44) $$\begin{array}{cc} X^{\left(2\right)}= & \;G^{\left(2\right)}\times_{1}U\times_{4}A^{\left(2\right)}. \end{array}$$

**Figure 10:**
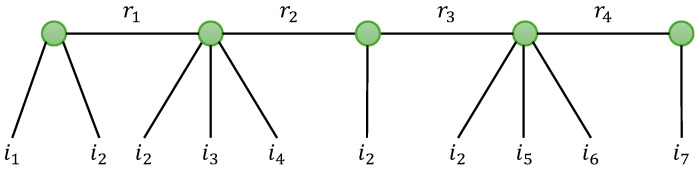
Graph of the NGTD-7 model for a seventh-order tensor $X\in K^{{\underline{I}}_{7}}$.

The NGTD-7 model is a GTTD-(3,5,3,5,2), represented in Figure 10. It corresponds to the nesting of the following two GTD-(2,5) models:

(45) $$\begin{array}{cc} X^{\left(1\right)}= & \;G^{\left(1\right)}\times_{1}^{3}A^{\left(1\right)}\times_{5}^{1}U\in K^{I_{1}\times I_{2}\times I_{3}\times I_{4}\times R_{3}} \end{array}$$

(46) $$\begin{array}{cc} X^{\left(2\right)}= & \;G^{\left(2\right)}\times_{1}^{3}U\times_{5}A^{\left(2\right)}\in K^{R_{2}\times I_{2}\times I_{5}\times I_{6}\times I_{7}}, \end{array}$$

which share the mode $i_{2}$ and the third-order tensor factor U. Equations (45) and (46) are to be compared with Equations (33) and (34) for the NTD-4 model. From this comparison, we deduce the correspondences $\left(I_{1},I_{2},I_{3},I_{4}\right)↔\left(I_{2}I_{1},I_{2}I_{3}I_{4},I_{2}I_{5}I_{6},I_{7}\right)$, and the contracted forms (36) and (37) become the following:

(47) $$\begin{array}{cc} X_{c_{1}}= & \;G^{\left(1\right)}\times_{1}^{3}A^{\left(1\right)}\times_{5}X_{I_{2}I_{5}I_{6}I_{7}\times R2}^{\left(2\right)}\in K^{I_{1}\times I_{2}\times I_{3}\times I_{4}\times I_{5}I_{6}I_{7}} \end{array}$$

(48) $$\begin{array}{cc} X_{c_{2}}= & \;G^{\left(2\right)}\times_{1}X_{I_{1}I_{2}I_{3}I_{4}\times R_{3}}^{\left(1\right)}\times_{5}A^{\left(2\right)}\in K^{I_{1}I_{2}I_{3}I_{4}\times I_{5}\times I_{6}\times I_{7}}. \end{array}$$

Sharing mode $i_{2}$ among the factors $\left(A^{\left(1\right)},G^{\left(1\right)},U,G^{\left(2\right)}\right)$ means that the Kronecker products in matrix unfoldings can be calculated separately for each value of $i_{2}\in \langle I_{2}\rangle$, using block-diagonal matrices composed of $I_{2}$ blocks, denoted as ${\mathrm{bdiag}}_{i_{2}}\left(.\right)$. From the GTD-(2,5) models (45) and (46) of $X^{\left(1\right)}$ and $X^{\left(2\right)}$, we deduce the following block-diagonal matrix unfoldings:

(49) $$\begin{array}{cc} {\mathrm{bdiag}}_{i_{2}}\left(X_{I_{1}R_{3}\times I_{3}I_{4}}^{\left(1\right)}\left(i_{2}\right)\right)= & \;{\mathrm{bdiag}}_{i_{2}}\left[A_{I_{1}\times R_{1}}^{\left(1\right)}\left(i_{2}\right)⊗U_{R_{3}\times R_{2}}\left(i_{2}\right)\right]{\mathrm{bdiag}}_{i_{2}}\left(G_{R_{1}R_{2}\times I_{3}I_{4}}^{\left(1\right)}\left(i_{2}\right)\right) \end{array}$$

(50) $$\begin{array}{cc} {\mathrm{bdiag}}_{i_{2}}\left(X_{I_{7}R_{2}\times I_{5}I_{6}}^{\left(2\right)}\left(i_{2}\right)\right)= & \;\left[A^{\left(2\right)}⊗{\mathrm{bdiag}}_{i_{2}}\left(U_{R_{2}\times R_{3}}\left(i_{2}\right)\right)\right]{\mathrm{bdiag}}_{i_{2}}(G_{R_{4}R_{3}\times I_{5}I_{6}}^{\left(2\right)}\left(i_{2}\right)), \end{array}$$

where the first term in the right side of (49) denotes a block-diagonal matrix for which each block on the diagonal is the Kronecker product of unfoldings of $A^{\left(1\right)}$ and U calculated for a given $i_{2}$.

Similarly, from the models (47) and (48) of $X_{c_{1}}$ and $X_{c_{2}}$, we have the following block-diagonal matrix unfoldings:

(51) $$\begin{array}{cc} {\mathrm{bdiag}}_{i_{2}}\left(X_{I_{1}I_{5}I_{6}I_{7}\times I_{3}I_{4}}\left(i_{2}\right)\right)= & \;{\mathrm{bdiag}}_{i_{2}}\left[A_{I_{1}\times R_{1}}^{\left(1\right)}\left(i_{2}\right)⊗X_{I_{5}I_{6}I_{7}\times R_{2}}^{\left(2\right)}\left(i_{2}\right)\right]{\mathrm{bdiag}}_{i_{2}}\left(G_{R_{1}R_{2}\times I_{3}I_{4}}^{\left(1\right)}\left(i_{2}\right)\right) \end{array}$$

(52) $$\begin{array}{cc} {\mathrm{bdiag}}_{i_{2}}\left(X_{I_{7}I_{1}I_{3}I_{4}\times I_{5}I_{6}}\left(i_{2}\right)\right)= & \;\left[A^{\left(2\right)}⊗{\mathrm{bdiag}}_{i_{2}}\left(X_{I_{1}I_{3}I_{4}\times R_{3}}^{\left(1\right)}\left(i_{2}\right)\right)\right]{\mathrm{bdiag}}_{i_{2}}(G_{R_{4}R_{3}\times I_{5}I_{6}}^{\left(2\right)}\left(i_{2}\right)). \end{array}$$

The matrix unfoldings (49)–(52) will be used in Section 4.5 to devise two closed-form parameter estimation algorithms.

In the cases of the NTD-6 and NGTD-7 models, Equation (39) becomes, respectively, the following:

(53) $$\begin{array}{cc} x_{{\underline{i}}_{6}}= & \;a_{i_{1},•}^{\left(1\right)}\;G_{•,i_{2},i_{3},•}^{\left(1\right)}\;U\;G_{•,i_{4},i_{5},•}^{\left(2\right)}\;a_{•,i_{6}}^{\left(2\right)} \end{array}$$

(54) $$\begin{array}{cc} x_{{\underline{i}}_{7}}= & \;a_{i_{1},i_{2},•}^{\left(1\right)}\;G_{•,i_{2},i_{3},i_{4},•}^{\left(1\right)}\;U_{•,i_{2},•}\;G_{•,i_{2},i_{5},i_{6},•}^{\left(2\right)}\;a_{•,i_{7}}^{\left(2\right)}. \end{array}$$

From these equations, we can draw the same conclusions as for the NTD-4 model, namely that the NTD-6 and NGTD-7 models are unique up to non-singular matrices $\Lambda^{\left(n\right)}\in K^{R_{n}\times R_{n}}$, n∈〈4〉, and that, when the tensors $G^{\left(1\right)}$ and $G^{\left(2\right)}$ are known, the ambiguity matrices $\Lambda^{\left(n\right)}$ become identity matrices multiplied by a scalar.

**Figure 11:**
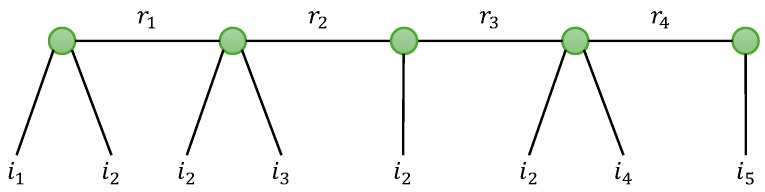
Graph of the NGTD-5 model for a fifth-order tensor $X\in K^{{\underline{I}}_{5}}$.

**Remark 3.**  *A simplified version of the NGTD-7 model, called NGTD-5 and represented in the graph in Figure 11, will be used in Section 5 to model the tensor of received signals in a relay system employing a simplified TSTF coding, denoted as STSTF. This model for a fifth-order tensor, $X\in K^{{\underline{I}}_{5}}$, corresponds to a generalized tensor train decomposition GTTD-(3,4,3,4,2), which results from the nesting of the following two GTD-(2,4) models:*

(55) $$\begin{array}{cc} X^{\left(1\right)}= & \;G^{\left(1\right)}\times_{1}^{3}A^{\left(1\right)}\times_{4}^{1}U\in K^{I_{1}\times I_{2}\times I_{3}\times R_{3}} \end{array}$$

(56) $$\begin{array}{cc} X^{\left(2\right)}= & \;G^{\left(2\right)}\times_{1}^{3}U\times_{5}A^{\left(2\right)}\in K^{R_{2}\times I_{2}\times I_{4}\times I_{5}}, \end{array}$$

*with*

(57) $$\begin{array}{cc} \; & A^{\left(1\right)}\in K^{I_{1}\times I_{2}\times R_{1}}\;\;\;;\;\;\;G^{\left(1\right)}\in K^{R_{1}\times I_{2}\times I_{3}\times R_{2}}\;\;\;;\;\;\;U\in K^{R_{2}\times I_{2}\times R_{3}} \end{array}$$

(58) $$\begin{array}{cc} \; & G^{\left(2\right)}\in K^{R_{3}\times I_{2}\times I_{4}\times R_{4}}\;\;;\;\;A^{\left(2\right)}\in K^{I_{5}\times R_{4}}. \end{array}$$

*Note that the tensor factors $\left(A^{\left(1\right)},G^{\left(1\right)},U,G^{\left(2\right)}\right)$ have the dimension $I_{2}$ in common, and the core tensors $G^{\left(1\right)}$ and $G^{\left(2\right)}$ are of fourth-order instead of fifth-order for NGTD-7.*

**Figure 12:**
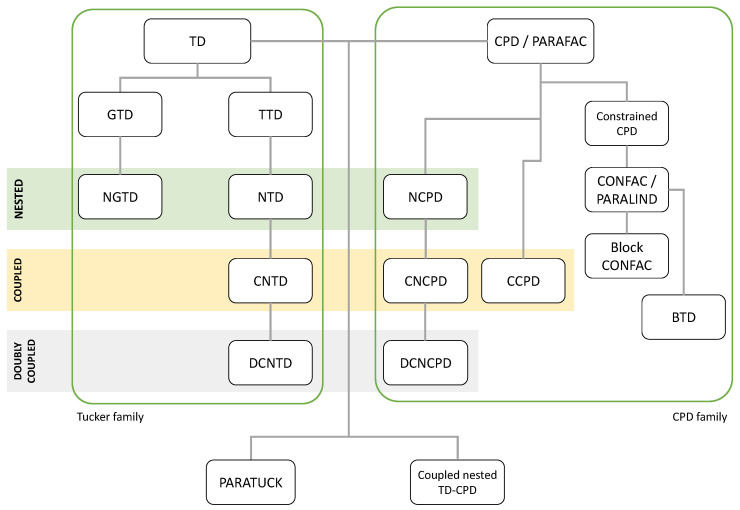
Two families of TD- and CPD-based decompositions.

In Figure 12, we summarize two families of tensor models using two trees, based on the basic CPD/PARAFAC and Tucker decompositions. For the definition of acronyms and associated references, see Appendix A.

Note that the NGTD and some variants of nested models are introduced, for the first time, in the present paper as generalizations of the NTD-4 model [41]. Nested tensor models will be used in Section 5 to represent two-hop relay systems using different tensor- and matrix-based codings. See Table 25.

**Remark 4.**  *The standard PARATUCK-2 model for a third-order tensor combines the properties of the PARAFAC and Tucker models. It was originally introduced in the context of psychometric applications [51]. In the context of wireless communications, a first application of the PARATUCK-2 model was proposed for blind joint identification and equalization of Wiener–Hammerstein nonlinear communication channels [52]. Then, it was used to model a two-hop relay system using simplified Khatri–Rao space-time (SKRST) coding [53]. An extension, denoted as PARATUCK-(2,4), was introduced in [37] to model the fourth-order tensor of signals received in a MIMO communication system with a tensor space-time (TST) coding.*

## 4. Parameter Estimation Algorithms

In this section, we present two families of algorithms to estimate the parameters of the tensor models introduced in the previous section: alternating least-squares (ALS)-based algorithms and closed-form algorithms. These latter use the Khatri–Rao and Kronecker factorization methods, respectively denoted as KRF and KronF, described in Appendix B and Appendix C. Closed-form algorithms can be applied when some factors of the models are known a priori, as will be the case with coding matrices and tensors, in the context of relay systems studied in Section 5. The standard ALS algorithm is first recalled for a third-order CPD/PARAFAC model.

### 4.1. CPD/PARAFAC Model

The ALS algorithm was first proposed to estimate the parameters of a CPD/PARAFAC model in [8,47]. Such a model for a third-order tensor X is trilinear in its parameters in the sense that it is linear with respect to each of its three matrix factors (A,B,C). When the relations (11) are taken into account, the idea behind the ALS algorithm is to replace the global minimization of the quadratic error between the data tensor and its CPD/PARAFAC model

(59) $$\min_{A,B,C}∥X-〚A,B,C;R〛{∥}_{F}^{2}$$

through an alternating minimization of three quadratic cost functions deduced from matrix unfoldings of X, each one being quadratic with respect to one factor, while the other two are fixed with their previously estimated values.

At iteration *t*, the minimization of each cost function provides an estimated matrix factor:

(60) $$\left\{\begin{array}{l} \min_{A}{∥X_{JK\times I}-\left(B_{t-1}⋄C_{t-1}\right)A^{T}∥}_{F}^{2}\;\;\Rightarrow \;\;A_{t}^{T}={\left(B_{t-1}⋄C_{t-1}\right)}^{†}\;X_{JK\times I}\\ \min_{B}{∥X_{KI\times J}-\left(C_{t-1}⋄A_{t}\right)B^{T}∥}_{F}^{2}\;\;\Rightarrow \;\;B_{t}^{T}={\left(C_{t-1}⋄A_{t}\right)}^{†}\;X_{KI\times J}\\ \min_{C}{∥X_{IJ\times K}-\left(A_{t}⋄B_{t}\right)C^{T}∥}_{F}^{2}\;\;\Rightarrow \;\;C_{t}^{T}={\left(A_{t}⋄B_{t}\right)}^{†}\;X_{IJ\times K} \end{array}\right.$$

where $A_{t}$ denotes the LS estimate of A at iteration *t*.

### 4.2. NCPD-4 Model

In the next two subsections, we first present a five-step ALS-based algorithm to estimate the five matrix factors $\left(A^{\left(1\right)},B^{\left(1\right)},G,A^{\left(2\right)},B^{\left(2\right)}\right)$ of the NCPD-4 model (21). The case where the factors $\left(B^{\left(1\right)},A^{\left(2\right)}\right)$ are known is also considered. Then, under this assumption, two closed-form algorithms using the KRF method are described. Necessary identifiability conditions are given for each identification algorithm.

#### 4.2.1. ALS Algorithm

Considering the first four matrix unfoldings in Table 14 with Equation (27), and applying the same approach as for the CPD model, we derive the following five LS cost functions to estimate the parameters of a NCPD-4 model:

(61) $$\begin{array}{cc} \; & \min_{B^{\left(1\right)}}{∥X_{I_{1}I_{3}I_{4}\times I_{2}}-\left[A_{t-1}^{\left(1\right)}⋄\left(A_{t-1}^{\left(2\right)}⋄B_{t-1}^{\left(2\right)}\right)G_{t-1}^{T}\right]{\left(B^{\left(1\right)}\right)}^{T}∥}_{F}^{2}\;\;\Rightarrow \;\;B_{t}^{\left(1\right)} \end{array}$$

(62) $$\begin{array}{cc} \; & \min_{A^{\left(1\right)}}{∥X_{I_{2}I_{3}I_{4}\times I_{1}}-\left[B_{t}^{\left(1\right)}⋄\left(A_{t-1}^{\left(2\right)}⋄B_{t-1}^{\left(2\right)}\right)G_{t-1}^{T}\right]{\left(A^{\left(1\right)}\right)}^{T}∥}_{F}^{2}\;\;\Rightarrow \;\;A_{t}^{\left(1\right)} \end{array}$$

(63) $$\begin{array}{cc} \; & \min_{B^{\left(2\right)}}{∥X_{I_{3}I_{1}I_{2}\times I_{4}}-\left[A_{t-1}^{\left(2\right)}⋄\left(A_{t}^{\left(1\right)}⋄B_{t}^{\left(1\right)}\right)G_{t-1}\right]{\left(B^{\left(2\right)}\right)}^{T}∥}_{F}^{2}\;\;\Rightarrow \;\;B_{t}^{\left(2\right)} \end{array}$$

(64) $$\begin{array}{cc} \; & \min_{A^{\left(2\right)}}{∥X_{I_{4}I_{1}I_{2}\times I_{3}}-\left[B_{t}^{\left(2\right)}⋄\left(A_{t}^{\left(1\right)}⋄B_{t}^{\left(1\right)}\right)G_{t-1}\right]{\left(A^{\left(2\right)}\right)}^{T}∥}_{F}^{2}\;\;\Rightarrow \;\;A_{t}^{\left(2\right)} \end{array}$$

(65) $$\begin{array}{cc} \; & \min_{G}{∥x_{I_{3}I_{4}I_{1}I_{2}}-\left(\left(A_{t}^{\left(2\right)}⋄B_{t}^{\left(2\right)}\right)⊗\left(A_{t}^{\left(1\right)}⋄B_{t}^{\left(1\right)}\right)\right)\mathrm{vec}\left(G\right)∥}_{F}^{2}\;\;\Rightarrow \;\;G_{t}. \end{array}$$

Minimizing the above LS criteria leads to the following equations for the ALS algorithm:

(66) $$\begin{array}{cc} \; & {\left(B_{t}^{\left(1\right)}\right)}^{T}={\left[A_{t-1}^{\left(1\right)}⋄\left(A_{t-1}^{\left(2\right)}⋄B_{t-1}^{\left(2\right)}\right)G_{t-1}^{T}\right]}^{†}X_{I_{1}I_{3}I_{4}\times I_{2}} \end{array}$$

(67) $$\begin{array}{cc} \; & {\left(A_{t}^{\left(1\right)}\right)}^{T}={\left[B_{t}^{\left(1\right)}⋄\left(A_{t-1}^{\left(2\right)}⋄B_{t-1}^{\left(2\right)}\right)G_{t-1}^{T}\right]}^{†}X_{I_{2}I_{3}I_{4}\times I_{1}} \end{array}$$

(68) $$\begin{array}{cc} \; & {\left(B_{t}^{\left(2\right)}\right)}^{T}={\left[A_{t-1}^{\left(2\right)}⋄\left(A_{t}^{\left(1\right)}⋄B_{t}^{\left(1\right)}\right)G_{t-1}\right]}^{†}X_{I_{3}I_{1}I_{2}\times I_{4}} \end{array}$$

(69) $$\begin{array}{cc} \; & {\left(A_{t}^{\left(2\right)}\right)}^{T}={\left[B_{t}^{\left(2\right)}⋄\left(A_{t}^{\left(1\right)}⋄B_{t}^{\left(1\right)}\right)G_{t-1}\right]}^{†}X_{I_{4}I_{1}I_{2}\times I_{3}} \end{array}$$

(70) $$\begin{array}{cc} \; & \mathrm{vec}\left(G_{t}\right)={\left(\left(A_{t}^{\left(2\right)}⋄B_{t}^{\left(2\right)}\right)⊗\left(A_{t}^{\left(1\right)}⋄B_{t}^{\left(1\right)}\right)\right)}^{†}x_{I_{3}I_{4}I_{1}I_{2}}. \end{array}$$

This algorithm requires the pseudo-inversion of five matrices, implying the following necessary identifiability conditions on the dimensions to guarantee the uniqueness of the pseudo-inverses:

(71) $$I_{1}I_{3}I_{4}\geq R_{1}\;;\;I_{2}I_{3}I_{4}\geq R_{1}\;;\;I_{3}I_{1}I_{2}\geq R_{2}\;;\;I_{4}I_{1}I_{2}\geq R_{2}\;;\;I_{3}I_{4}I_{1}I_{2}\geq R_{1}R_{2}.$$

As is well known, the main drawback of iterative algorithms based on ALS is slow convergence, with the possibility of converging to a local minimum, which strongly depends on the choice of initialization.

When the factors $\left(B^{\left(1\right)},A^{\left(2\right)}\right)$ are known, as will be the case with coding matrices in the context of relay systems, a three-step ALS algorithm composed of Equations (67), (68) and (70) can be used to estimate the unknown factors $\left(A^{\left(1\right)},B^{\left(2\right)},G\right)$.

#### 4.2.2. KRF-Based Closed-Form Algorithms

As mentioned in Section 3.3.1, when the factors $\left(B^{\left(1\right)},A^{\left(2\right)}\right)$ are known, two closed-form algorithms using the KRF method can be devised as shown hereafter. These algorithms, composed of two steps, are deduced from unfoldings (in green) in Table 13.

The first algorithm is based on the following matrix unfoldings of $X_{c_{2}}$ and $X^{\left(1\right)}$ deduced from the coupled CPD-3 models (26) and (19), respectively:

(72) $$\begin{array}{cc} X_{I_{4}I_{1}I_{2}\times I_{3}}= & \;\left(B^{\left(2\right)}⋄X_{I_{1}I_{2}\times R_{2}}^{\left(1\right)}\right){\left(A^{\left(2\right)}\right)}^{T} \end{array}$$

(73) $$\begin{array}{cc} X_{I_{1}R_{2}\times I_{2}}^{\left(1\right)}= & \;\left(A^{\left(1\right)}⋄G^{T}\right){\left(B^{\left(1\right)}\right)}^{T}. \end{array}$$

These equations are successively exploited as follows. In a first step, the LS estimate of the Khatri–Rao product (KRP) in (72) is calculated, and its factors $\left(B^{\left(2\right)},X_{I_{1}I_{2}\times R_{2}}^{\left(1\right)}\right)$ are determined using the KRF method. In the second step, after a reshaping of the estimate ${\hat{X}}_{I_{1}I_{2}\times R_{2}}^{\left(1\right)}$, the LS estimate of the KRP in (73), with $X_{I_{1}R_{2}\times I_{2}}^{\left(1\right)}$ replaced with its estimate ${\hat{X}}_{I_{1}R_{2}\times I_{2}}^{\left(1\right)}$ deduced from the reshaping, is calculated, and the KRF method is applied again to estimate the factors $\left(A^{\left(1\right)},G^{T}\right)$. The resulting algorithm, namely Closed-Form Algorithm 1, is summarized in Table 18.

Similarly, a second closed-form solution can be derived from the following coupled matrix unfoldings of $X_{c_{1}}$ and $X^{\left(2\right)}$ deduced from the CPD-3 models (25) and (20), respectively:

(74) $$\begin{array}{cc} X_{I_{1}I_{3}I_{4}\times I_{2}}= & \;\left(A^{\left(1\right)}⋄X_{I_{3}I_{4}\times R_{1}}^{\left(2\right)}\right){\left(B^{\left(1\right)}\right)}^{T} \end{array}$$

(75) $$\begin{array}{cc} X_{I_{4}R_{1}\times I_{3}}^{\left(2\right)}= & \;\left(B^{\left(2\right)}⋄G\right){\left(A^{\left(2\right)}\right)}^{T}. \end{array}$$

Following the same approach as for the Closed-Form Algorithm 1, the Closed-Form Algorithm 2 is summarized in Table 18.

Identifiability conditions for the closed-form algorithms are linked with the uniqueness of right inverses of ${\left(B^{\left(1\right)}\right)}^{T}$ and ${\left(A^{\left(2\right)}\right)}^{T}$, which yields the following necessary conditions:

(76) $$I_{2}\geq R_{1}\;\;\;\mathrm{and}\;\;\;I_{3}\geq R_{2}.$$

Comparing the identifiability conditions (76) with (71), one can conclude that the ALS algorithm is less constraining than the closed-form ones.

**Remark 5.**  *It is worth noting that closed-form algorithms involve the drawback of error propagation for computing the LS estimate of the KRP in the second step due to the use of the estimates ${\hat{X}}_{I_{1}I_{2}\times R_{2}}^{\left(1\right)}$ and ${\hat{X}}_{I_{3}I_{4}\times R_{1}}^{\left(2\right)}$ obtained in the first step. To alleviate error propagation, a solution consists of combining both closed-form algorithms to estimate the factors $\left(A^{\left(1\right)},B^{\left(2\right)}\right)$ in parallel. Then, the factor G is estimated using one of the following formulae deduced from the first and third equations in Table 13:*

(77) $$\hat{G}={\left({\hat{A}}^{\left(1\right)}⋄B^{\left(1\right)}\right)}^{†}{\hat{X}}_{I_{1}I_{2}\times R_{2}}^{\left(1\right)}\;\;\;\mathrm{or}\;\;\;{\hat{G}}^{T}={\left(A^{\left(2\right)}⋄{\hat{B}}^{\left(2\right)}\right)}^{†}{\hat{X}}_{I_{3}I_{4}\times R_{1}}^{\left(2\right)},$$

*where $\left({\hat{A}}^{\left(1\right)},{\hat{X}}_{I_{1}I_{2}\times R_{2}}^{\left(1\right)}\right)$ and $\left({\hat{B}}^{\left(2\right)},{\hat{X}}_{I_{3}I_{4}\times R_{1}}^{\left(2\right)}\right)$ are estimates obtained with the closed-form algorithms. The identifiability conditions for these combined closed-form algorithms are given by (76) with additional conditions resulting from the pseudo-inverses to be calculated in (77):*

(78) $$I_{1}I_{2}\geq R_{1}\;\;\;\mathrm{and}\;\;\;I_{3}I_{4}\geq R_{2}.$$

*These conditions are necessarily satisfied when conditions (76) are.*

### 4.3. NTD-4 Model

In the following, we assume that the core tensors $\left(G^{\left(1\right)},G^{\left(2\right)}\right)$ are known, and we describe a three-step ALS-based algorithm and two closed-form algorithms to estimate the factors $\left(A^{\left(1\right)},U,A^{\left(2\right)}\right)$ of the NTD-4 model.

#### 4.3.1. ALS Algorithm

As the LS cost functions (61)–(65) for the NCPD-4 model, unfoldings in Table 16 can be used to define three LS criteria to be minimized in order to estimate the matrix factors $\left(A^{\left(1\right)},U,A^{\left(2\right)}\right)$ by means of a three-step ALS-based algorithm. From the first, third, and fifth equations (in blue) in Table 16, we define the following LS cost functions:

(79) $$\begin{array}{cc} \; & \min_{A^{\left(2\right)}}{∥X_{I_{3}I_{1}I_{2}\times I_{4}}-\left[I_{I_{3}}⊗\left(A_{t-1}^{\left(1\right)}⊗I_{I_{2}}\right)G_{R_{1}I_{2}\times R_{2}}^{\left(1\right)}U_{t-1}\right]G_{I_{3}R_{3}\times R_{4}}^{\left(2\right)}{\left(A^{\left(2\right)}\right)}^{T}∥}_{F}^{2}\;\;\Rightarrow \;\;A_{t}^{\left(2\right)} \end{array}$$

(80) $$\begin{array}{cc} \; & \min_{A^{\left(1\right)}}{∥X_{I_{2}I_{3}I_{4}\times I_{1}}-\left[I_{I_{2}}⊗\left(I_{I_{3}}⊗A_{t}^{\left(2\right)}\right)G_{I_{3}R_{4}\times R_{3}}^{\left(2\right)}U_{t-1}^{T}\right]G_{I_{2}R_{2}\times R_{1}}^{\left(1\right)}{\left(A^{\left(1\right)}\right)}^{T}∥}_{F}^{2}\;\;\Rightarrow \;\;A_{t}^{\left(1\right)} \end{array}$$

(81) $$\begin{array}{cc} \; & \min_{U}{∥x_{I_{3}I_{4}I_{1}I_{2}}-\left[\left(I_{I_{3}}⊗A_{t}^{\left(2\right)}\right)G_{I_{3}R_{4}\times R_{3}}^{\left(2\right)}⊗\left(A_{t}^{\left(1\right)}⊗I_{I_{2}}\right)G_{R_{1}I_{2}\times R_{2}}^{\left(1\right)}\right]\mathrm{vec}\left(U\right)∥}_{F}^{2}\;\;\Rightarrow \;\;U_{t}. \end{array}$$

Minimizing these LS criteria leads to the following equations for the ALS algorithm:

(82) $$\begin{array}{cc} \; & {\left(A_{t}^{\left(2\right)}\right)}^{T}={\left[\left[I_{I_{3}}⊗\left(A_{t-1}^{\left(1\right)}⊗I_{I_{2}}\right)G_{R_{1}I_{2}\times R_{2}}^{\left(1\right)}U_{t-1}\right]G_{I_{3}R_{3}\times R_{4}}^{\left(2\right)}\right]}^{†}X_{I_{3}I_{1}I_{2}\times I_{4}} \end{array}$$

(83) $$\begin{array}{cc} \; & {\left(A_{t}^{\left(1\right)}\right)}^{T}={\left[\left[I_{I_{2}}⊗\left(I_{I_{3}}⊗A_{t}^{\left(2\right)}\right)G_{I_{3}R_{4}\times R_{3}}^{\left(2\right)}U_{t-1}^{T}\right]G_{I_{2}R_{2}\times R_{1}}^{\left(1\right)}\right]}^{†}X_{I_{2}I_{3}I_{4}\times I_{1}} \end{array}$$

(84) $$\begin{array}{cc} \; & \mathrm{vec}\left(U_{t}\right)={\left[\left(I_{I_{3}}⊗A_{t}^{\left(2\right)}\right)G_{I_{3}R_{4}\times R_{3}}^{\left(2\right)}⊗\left(A_{t}^{\left(1\right)}⊗I_{I_{2}}\right)G_{R_{1}I_{2}\times R_{2}}^{\left(1\right)}\right]}^{†}x_{I_{3}I_{4}I_{1}I_{2}}. \end{array}$$

The uniqueness of the above LS solutions implies the following necessary identifiability conditions in the dimensions:

(85) $$I_{3}I_{1}I_{2}\geq R_{4}\;;\;I_{2}I_{3}I_{4}\geq R_{1}\;;\;I_{3}I_{4}I_{1}I_{2}\geq R_{3}R_{2}.$$

#### 4.3.2. Closed-Form Algorithms

Following the same approach as for the NCPD-4 model, we now present two closed-form solutions exploiting the matrix unfoldings (in green) in Table 15 and using the KronF method. From the coupled TD-(2,3) models (37) and (33) of $X_{c_{2}}$ and $X^{\left(1\right)}$, we deduce the following matrix unfoldings of the tensors X and $X^{\left(1\right)}$:

(86) $$\begin{array}{cc} X_{I_{4}I_{1}I_{2}\times I_{3}}= & \;\left(A^{\left(2\right)}⊗X_{I_{1}I_{2}\times R_{3}}^{\left(1\right)}\right)G_{R_{4}R_{3}\times I_{3}}^{\left(2\right)} \end{array}$$

(87) $$\begin{array}{cc} X_{I_{1}R_{3}\times I_{2}}^{\left(1\right)}= & \;\left(A^{\left(1\right)}⊗U^{T}\right)G_{R_{1}R_{2}\times I_{2}}^{\left(1\right)}. \end{array}$$

These equations are used to build the first closed-form algorithm as follows: In a first step, the LS method is applied to estimate the Kronecker product (KronP) in Equation (86) as follows:

(88) $$A^{\left(2\right)}⊗X_{I_{1}I_{2}\times R_{3}}^{\left(1\right)}=X_{I_{4}I_{1}I_{2}\times I_{3}}{\left[G_{R_{4}R_{3}\times I_{3}}^{\left(2\right)}\right]}^{†}.$$

The factors $\left(A^{\left(2\right)},X_{I_{1}I_{2}\times R_{3}}^{\left(1\right)}\right)$ are then estimated using the KronF method. In a second step, after a reshaping of the estimate ${\hat{X}}_{I_{1}I_{2}\times R_{3}}^{\left(1\right)}$, the LS method is used to estimate the KronP in Equation (87) as follows:

(89) $$A^{\left(1\right)}⊗U^{T}={\hat{X}}_{I_{1}R_{3}\times I_{2}}^{\left(1\right)}{\left(G_{R_{1}R_{2}\times I_{2}}^{\left(1\right)}\right)}^{†}.$$

The factors $\left(A^{\left(1\right)},U\right)$ are then deduced using the KronF method. This two-step Closed-Form Algorithm 1 is summarized in Table 19.

The second closed-form solution is based on the following matrix unfoldings deduced from the coupled TD-(2,3) models (36) and (34) of $X_{c_{1}}$ and $X^{\left(2\right)}$:

(90) $$\begin{array}{cc} X_{I_{1}I_{3}I_{4}\times I_{2}}= & \;\left(A^{\left(1\right)}⊗X_{I_{3}I_{4}\times R_{2}}^{\left(2\right)}\right)G_{R_{1}R_{2}\times I_{2}}^{\left(1\right)} \end{array}$$

(91) $$\begin{array}{cc} X_{I_{4}R_{2}\times I_{3}}^{\left(2\right)}= & \;\left(A^{\left(2\right)}⊗U\right)G_{R_{4}R_{3}\times I_{3}}^{\left(2\right)}, \end{array}$$

which leads to Closed-Form Algorithm 2, summarized in Table 19.

For these closed-form solutions, necessary identifiability conditions, associated with the right inversion of $G_{R_{1}R_{2}\times I_{2}}^{\left(1\right)}$ and $G_{R_{4}R_{3}\times I_{3}}^{\left(2\right)}$, are given by the following:

(92) $$I_{2}\geq R_{1}R_{2}\;\;\;\mathrm{and}\;\;\;I_{3}\geq R_{3}R_{4}.$$

These conditions are to be compared with the ALS ones (85).

**Remark 6.**  *As for the NCPD-4 model, the closed-form algorithms in Table 19 involve the drawback of error propagation due to the use, in the second step, of the estimates ${\hat{X}}_{I_{1}I_{2}\times R_{3}}^{\left(1\right)}$ and ${\hat{X}}_{I_{3}I_{4}\times R_{2}}^{\left(2\right)}$ obtained in the first step. An improved solution consists of combining both closed-form algorithms to estimate the factors $\left(A^{\left(1\right)},A^{\left(2\right)}\right)$ in parallel. Then, the factor U is estimated using one of the following formulae deduced from the expressions of $X_{I_{1}I_{2}\times R_{3}}^{\left(1\right)}$ and $X_{I_{3}I_{4}\times R_{2}}^{\left(2\right)}$ given in Table 15:*

(93) $$\hat{U}={\left(\left({\hat{A}}^{\left(1\right)}⊗I_{I_{2}}\right)G_{R_{1}I_{2}\times R_{2}}^{\left(1\right)}\right)}^{†}{\hat{X}}_{I_{1}I_{2}\times R_{3}}^{\left(1\right)}\;\;\mathrm{or}\;\;{\hat{U}}^{T}={\left(\left(I_{I_{3}}⊗{\hat{A}}^{\left(2\right)}\right)G_{I_{3}R_{4}\times R_{3}}^{\left(2\right)}\right)}^{†}{\hat{X}}_{I_{3}I_{4}\times R_{2}}^{\left(2\right)},$$

*where $\left({\hat{A}}^{\left(1\right)},{\hat{A}}^{\left(2\right)},{\hat{X}}_{I_{1}I_{2}\times R_{3}}^{\left(1\right)},{\hat{X}}_{I_{3}I_{4}\times R_{2}}^{\left(2\right)}\right)$ are estimates obtained in the first step of the closed-form algorithms. Identifiability conditions for these combined closed-form algorithms are given by (92) with additional conditions for the pseudo-inverses to be calculated in (93):*

(94) $$I_{1}I_{2}\geq R_{2}\;\;\;\mathrm{and}\;\;\;I_{3}I_{4}\geq R_{3}.$$

*Note that these conditions are always satisfied when conditions (92) are.*

### 4.4. NTD-6 Model

The closed-form algorithms for the NTD-4 model are generalized to the NTD-6 using the correspondences $\left(I_{2},I_{3},I_{4}\right)↔\left(I_{2}I_{3},I_{4}I_{5},I_{6}\right)$, as summarized in Table 20.

Necessary identifiability conditions for NTD-6 are associated with the uniqueness of the right inverses of $G_{R_{1}R_{2}\times I_{2}I_{3}}^{\left(1\right)}$ and $G_{R_{4}R_{3}\times I_{4}I_{5}}^{\left(2\right)}$, which gives the following:

(95) $$I_{2}I_{3}\geq R_{1}R_{2}\;\;\;\mathrm{and}\;\;\;I_{4}I_{5}\geq R_{3}R_{4}.$$

### 4.5. NGTD-7 Model

Assuming the core tensors $\left(G^{\left(1\right)},G^{\left(2\right)}\right)$ are known, we now present two closed-form algorithms to estimate the parametters $\left(A^{\left(1\right)},U,A^{\left(2\right)}\right)$ of the NGTD-7 model introduced in Table 17. The first one is based on the coupled matrix unfoldings (52) and (49) of X and $X^{\left(1\right)}$. Equation (52) is used to determine the LS estimate of the KronF product from which the factors $\left(A^{\left(2\right)},{\mathrm{bdiag}}_{i_{2}}\left(X_{I_{1}I_{3}I_{4}\times R_{3}}^{\left(1\right)}\left(i_{2}\right)\right)\right)$ are determined by applying the KronF method. Then, after a reshaping of ${\mathrm{bdiag}}_{i_{2}}\left({\hat{X}}_{I_{1}I_{3}I_{4}\times R_{3}}^{\left(1\right)}\left(i_{2}\right)\right))$, the LS estimate of the KronF product in (49) is calculated, and the factors $\left({\mathrm{bdiag}}_{i_{2}}\left(A_{I_{1}\times R_{1}}^{\left(1\right)}\left(i_{2}\right)\right),{\mathrm{bdiag}}_{i_{2}}\left(U_{R_{3}\times R_{2}}\left(i_{2}\right)\right)\right)$ are determined via the KronF method.

The resulting closed-form algorithm for NGTD-7 is summarized in Table 21, which also contains the second closed-form method based on the coupled matrix unfoldings (51) and (50), and derived following the same approach. To simplify the writing of equations in Table 21, the term “$\left(i_{2}\right)$” is dropped from the block-diagonal matrices ${\mathrm{bdiag}}_{i_{2}}\left(.\right)$.

For both closed-form algorithms, necessary identifiability conditions are associated with the uniqueness of the right inverses of $\mathrm{bdiag}(G_{R_{1}R_{2}\times I_{3}I_{4}}^{\left(1\right)})$ and $\mathrm{bdiag}(G_{R_{4}R_{3}\times I_{5}I_{6}}^{\left(2\right)})$, which implies the following:

(96) $$I_{3}I_{4}\geq R_{1}R_{2}\;\;\;\mathrm{and}\;\;\;I_{5}I_{6}\geq R_{3}R_{4}.$$

**Remark 7.**  *The closed-form algorithms for the NGTD-5 model can be deduced from the ones for the NGTD-7 model using the following correspondences: $\left(I_{3}I_{4},I_{5}I_{6},I_{7}\right)↔\left(I_{3},I_{4},I_{5}\right)$. The necessary identifiability conditions for NGTD-5, associated with the uniqueness of the right inverses of $\mathrm{bdiag}(G_{R_{1}R_{2}\times I_{3}}^{\left(1\right)})$ and $\mathrm{bdiag}(G_{R_{4}R_{3}\times I_{4}}^{\left(2\right)})$, are then given by the following:*

(97) $$I_{3}\geq R_{1}R_{2}\;\;\;\mathrm{and}\;\;\;I_{4}\geq R_{3}R_{4}.$$

## 5. Overview of Cooperative and Two-Hop Relay Systems

In this section, we first present an overview of tensor-based MIMO cooperative systems from a semi-blind receiver perspective. Then, we provide a detailed presentation of main two-hop relay systems.

### 5.1. Overview of Cooperative Systems

Table 22 completes Table 9 of the companion paper [45]. This table mentions the cases of OFDM/mmW communications, the type of cooperation (relay/IRS/UAV), the coding, and the tensor model of signals received at destination. It also includes the receiver algorithms used for each cooperative system.

Hereafter, we highlight some important characteristics of cooperative systems to motivate the choice of two-hop relay systems taken into account in our comparative study:

- *Cooperation scheme:* Most cooperative systems use relays for the exchange of information between source and destination nodes. Relay stations are equipped with hardware and signal processing capability that allow signal decoding/coding steps, depending on the relay protocol. Recent works have addressed cooperation schemes using intelligent reflecting surfaces (IRSs), which act as a large number of passive re-reflecting elements with low energy consumption and limited processing capacity to process the received signals. For a comprehensive presentation of IRS-assisted MIMO communication systems and, more generally, of various applications of IRS-assisted wireless networks, the reader is referred to the following review papers: [75,76,77,78,79,80].
  Note that, unlike the semi-blind receivers proposed in this paper that allow an estimation of individual channels in two-hop relay systems with the use of very few pilot symbols, most existing works regarding IRS-assisted wireless communications have presented supervised solutions using a pilot sequence to estimate the individual transmitter-to-IRS and IRS-to-receiver channels or the cascaded transmitter–IRS receiver channel, view as a single channel [65,68,69,81,82,83,84,85]. The use of pilot sequences results in a reduction in transmission rates.
  Channel estimation for IRS-assisted MIMO communication systems is a very challenging task due to the following: (i) the large number of passive IRS elements and, therefore, of channel coefficients to be estimated; and (ii) the lack of processing capacity at the IRS.
  A lot of methods have been proposed in the literature to solve the channel estimation problem, depending on the channel model, system configuration (SISO/MISO/MIMO, single-/multi-user, single-/multi-IRS, or narrowband/broadband communication), type of receiver (supervised using pilot sequences versus semi-blind), and algorithm (ALS, closed-form, compressed sensing, or deep learning) used for individual or cascaded channel estimation. The reader is invited to consult the survey paper [86] for a more detailed presentation of IRS channel estimation methods.
  Another type of cooperative system involves UAV-assisted communications [70,87]. Consult [88] for a survey on civil UAV applications. In the context of 6G wireless networks, from the perspective of connecting everyone and everything, integrated satellite–terrestrial networks [89], also known as integrated satellite terrestrial/aerial networks (IST/ANs) [90], have been the subject of recent studies. Other recent works consider IRS-UAV-assisted wireless communication networks for assisting the communication between a base station and multiple users [91,92] or an Internet of Things (IoT) terminal [71].
  In the future, highly digitized world of all connected objects, 6G wireless networks will integrate different functionalities, including sensing, communication, computing, localization, navigation, signal and image processing, and object recognition, combined with artificial intelligence (AI) technology. This is the case of recent integrated (radar) sensing and communication (ISAC) systems [93,94,95], also known as joint communication and radar/radio sensing (JCAS) systems [96], which aim to improve spectral efficiency while minimizing hardware cost and power consumption.
- *Relaying protocols:* The two most common relaying protocols are DF and AF, depending on whether the relay decodes the received signals. Although the DF protocol generally offers better performance, most of the relay systems presented in Table 22 use the AF protocol. In [55], the authors compare the system performance obtained with both protocols to jointly and semi-blindly estimate the transmitted symbols and individual channels in a one-way two-hop relay system.
- *Coding schemes:* Various coding schemes are used in the cooperative systems reported in Table 22. However, much of the works propose cooperative systems employing KRST and TST codings. In the present paper, we consider two coding families: tensor-based (e.g., TST and TSTF, and simplified versions denoted as STST and STSTF) and matrix-based (e.g., SKRST and DKRSTF) codings employed at the source and the relay. Two combined codings, denoted as SKRST-MSMKR and STST-MSMKron, are also considered.
- *Communication channels:* Two types of channels are most often considered, namely flat-fading and frequency-selective fading channels. In the latter case, the channels, which depend on frequency, are represented by means of third-order tensors, implying more channel coefficients to be estimated.
- *Receiver algorithms:* Parameter estimation algorithms can be classified into two main categories: iterative and closed-form algorithms. The works cited in Table 22 mainly use ALS-based algorithms and closed-form solutions based on KRF and KronF methods, described respectively in Appendix B and Appendix C. These algorithms will be applied in Section 6 to devise semi-blind receivers for the joint estimation of symbol matrices and channel coefficients for all considered relay systems.

### 5.2. Overview of Two-Hop Relay Systems

Relay systems can be classified according to the following characteristics:

- One-way/two-way;
- AF/DF relaying protocol;
- Two-hop/multihop;
- Half-duplex/full-duplex (the relay can then transmit and receive simultaneously in both directions, during the same time slot).

In Section 5.3, Section 5.4 and Section 5.5, we provide an overview of one-way half-duplex two-hop relay systems, applying several coding schemes that take into account different types of diversity, which results in different tensor models for the received signals. The codings considered are the following: tensor space-time-frequency (TSTF), tensor space-time (TST), simplified TSTF (STSTF), simplified TST (STST), simplified Khatri–Rao space-time (SKRST), and double Khatri–Rao space-time-frequency (DKRSTF), all using the AF protocol.

Additionally, to provide a comparison with the DF protocol, we consider STST and SKRST combined with MSMKron and MSMKR codings, as proposed in [97,98] in the context of point-to-point systems.

**Figure 13:**
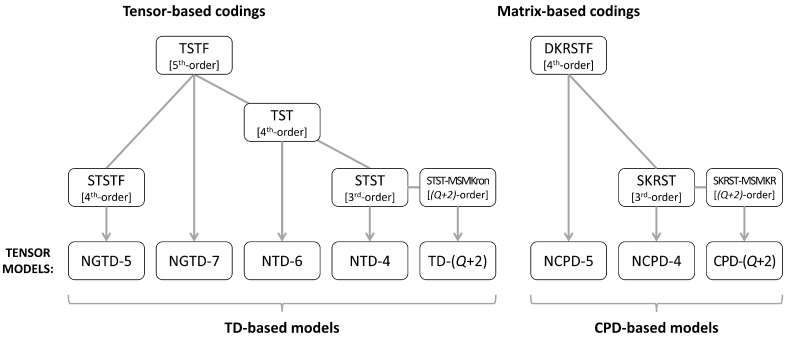
Classification of relay systems according to the coding scheme and tensor model.

The relay systems considered can be classified according to different choices concerning the coding scheme, relaying protocol, tensor model, and receiver algorithm. Figure 13 illustrates this classification according to the coding structure, mentioning the order of the tensor of encoded signals to be transmitted and specifying the tensor model for the signals received at the destination node. It is noteworthy that certain codings are particular cases of more generic ones, resulting from simple restrictions in terms of diversities taken into account, which induces simplified tensor models.

**Figure 14:**
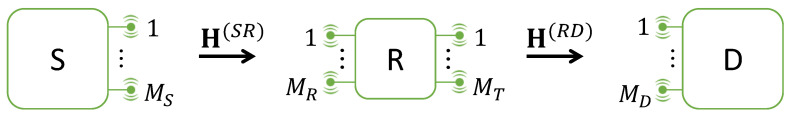
One-way, two-hop cooperative system.

Let us introduce the general framework of a one-way, two-hop relay system, as shown in Figure 14, which is composed of source (S), relay (R), and destination (D) nodes, assumed to be equipped with multiple antennas: $M_{S}$ transmit and $M_{D}$ receive antennas at the source and destination, respectively, while the relay has $M_{R}$ receive and $M_{T}$ transmit antennas. Note that, in this paper, the direct link between the source and the destination is assumed to be unavailable. Table 23 summarizes the definitions of the design parameters and the matrices and tensors used to describe all the relay systems.

In the following, we consider three classes of coding: tensor-based codings in Section 5.3, matrix-based codings in Section 5.4, and tensor- and matrix-based codings combined with MSMKron and MSMKR codings, respectively, in Section 5.5. With the first two classes of coding, the AF relaying protocol will be used, while the DF protocol will be considered in the case of combined codings.

### 5.3. AF Two-Hop Relay Systems Using Tensor-Based Codings

High-order tensors have been used to define tensor coding schemes, as proposed for the first time with the TST coding for CDMA systems [37] and the TSTF coding for OFDM systems [38], in the context of point-to-point wireless communications, in 2012 and 2014, respectively. Let us start our survey by presenting the most general two-hop system using fifth-order TSTF codings at both the source and relay, from which simpler systems will be straightforwardly derived. To alleviate the presentation, we consider the noiseless case. The noise will be introduced in the experimental study, in Section 7.

#### 5.3.1. TSTF Coding

Fifth-order TSTF codings $C^{\left(S\right)}\in C^{M_{S}\times F\times P_{S}\times J_{S}\times R}$ and $C^{\left(R\right)}\in C^{M_{T}\times F\times P_{R}\times J_{R}\times M_{R}}$ are employed at the source and relay, respectively, with the AF protocol at the relay. The channels between the source and the relay (SR), and between the relay and the destination (RD), are assumed to be frequency-selective fading and quasic-static, i.e., time-invariant during the transmission, composed of $P_{S}$ and $P_{R}$ time slots at the source and relay, respectively. They are represented by third-order tensors $H^{\left(SR\right)}\in C^{M_{R}\times F\times M_{S}}$ and $H^{\left(RD\right)}\in C^{M_{D}\times F\times M_{R}}$.

Let us consider the first hop, detailing the coding at the source and the transmission from the source to the relay. The symbol matrix $S\in C^{N\times R}$ to be transmitted contains *R* data streams, each one composed of *N* information symbols. At the source, the *n*th symbols $s_{n,r}$ of the *R* data streams are linearly combined and duplicated in the space–frequency–time–chip domains, owing to the coding tensor $C^{\left(S\right)}$, via the following equation, which defines the fifth-order tensor $U^{\left(S\right)}\in C^{M_{S}\times F\times P_{S}\times J_{S}\times N}$ of coded signals:

(98) $$U^{\left(S\right)}=C^{\left(S\right)}\times_{5}S\;\;↔\;\;u_{m_{S},f,p_{S},j_{S},n}^{\left(S\right)}=\sum_{r=1}^{R}c_{m_{S},f,p_{S},j_{S},r}^{\left(S\right)}s_{n,r}.$$

This coding is associated with a transmission during $P_{S}$ time slots composed of *N* symbol periods each, a time spreading composed of $J_{S}$ chips, and a repetition over *F* subcarriers.

The coded signals are transmitted via the $M_{S}$ transmit antennas from the source to the relay through the transmission channel $H^{\left(SR\right)}$. The $m_{R}$th antenna of the relay receives during the $j_{S}$th chip period of the *n*th symbol period of the $p_{S}$th time slot, and associated with the *f*th subcarrier, the signal given by the following:

(99) $$X^{\left(R\right)}=U^{\left(S\right)}\times_{1}^{3}H^{\left(SR\right)}=H^{\left(SR\right)}\times_{3}^{1}U^{\left(S\right)}\;\;↔\;\;x_{m_{R},f,p_{S},j_{S},n}^{\left(R\right)}=\sum_{m_{S}=1}^{M_{S}}h_{m_{R},f,m_{S}}^{\left(SR\right)}u_{m_{S},f,p_{S},j_{S},n}^{\left(S\right)}.$$

Combining Equations (98) and (99) allows for rewriting the fifth-order tensor $X^{\left(R\right)}\in C^{M_{R}\times F\times P_{S}\times J_{S}\times N}$ of signals received at the relay as follows:

(100) $$X^{\left(R\right)}=H^{\left(SR\right)}\times_{3}^{1}C^{\left(S\right)}\times_{5}S\;\;↔\;\;x_{m_{R},f,p_{S},j_{S},n}^{\left(R\right)}=\sum_{m_{S}=1}^{M_{S}}\sum_{r=1}^{R}h_{m_{R},f,m_{S}}^{\left(SR\right)}c_{m_{S},f,p_{S},j_{S},r}^{\left(S\right)}s_{n,r},$$

or equivalently:

(101) $$X^{\left(R\right)}=C^{\left(S\right)}\times_{1}^{3}H^{\left(SR\right)}\times_{5}S,$$

which is a GTD-(2,5) model whose core tensor is the source-coding tensor $C^{\left(S\right)}$.

We now follow the same reasoning for the second hop. Due to the AF protocol, the signals received at the relay are re-encoded using the coding tensor $C^{\left(R\right)}\in C^{M_{T}\times F\times P_{R}\times J_{R}\times M_{R}}$ to give the seventh-order tensor $U^{\left(R\right)}\in C^{M_{T}\times F\times P_{R}\times J_{R}\times P_{S}\times J_{S}\times N}$ of coded signals:

(102) $$U^{\left(R\right)}=C^{\left(R\right)}\times_{5}^{1}X^{\left(R\right)}\;\;↔\;\;u_{m_{T},f,p_{R},j_{R},p_{S},j_{S},n}^{\left(R\right)}=\sum_{m_{R}=1}^{M_{R}}c_{m_{T},f,p_{R},j_{R},m_{R}}^{\left(R\right)}x_{m_{R},f,p_{S},j_{S},n}^{\left(R\right)}.$$

These coded signals are transmitted via the relay to the destination through the channel $H^{\left(RD\right)}$, giving the seventh-order tensor $X^{\left(D\right)}\in C^{M_{D}\times F\times P_{R}\times J_{R}\times P_{S}\times J_{S}\times N}$ of signals received at the destination:

(103) $$X^{\left(D\right)}=H^{\left(RD\right)}\times_{3}^{1}U^{\left(R\right)}\;\;↔\;\;x_{m_{D},f,p_{R},j_{R},p_{S},j_{S},n}^{\left(D\right)}=\sum_{m_{T}=1}^{M_{T}}h_{m_{D},f,m_{T}}^{\left(RD\right)}u_{m_{T},f,p_{R},j_{R},p_{S},j_{S},n}^{\left(R\right)}.$$

Replacing $U^{\left(R\right)}$, $X^{\left(R\right)}$ and $U^{\left(S\right)}$ with their expressions (102), (99) and (98) gives the following:

(104) $$\begin{array}{cc} X^{\left(D\right)}= & \;H^{\left(RD\right)}\times_{3}^{1}C^{\left(R\right)}\times_{5}^{1}X^{\left(R\right)} \end{array}$$

(105) $$\begin{array}{cc} \;= & \;H^{\left(RD\right)}\times_{3}^{1}C^{\left(R\right)}\times_{5}^{1}H^{\left(SR\right)}\times_{5}^{1}U^{\left(S\right)} \end{array}$$

(106) $$\begin{array}{cc} \;= & \;H^{\left(RD\right)}\times_{3}^{1}C^{\left(R\right)}\times_{5}^{1}H^{\left(SR\right)}\times_{5}^{1}C^{\left(S\right)}\times_{7}S \end{array}$$

or in scalar form:

(107) $$x_{m_{D},f,p_{R},j_{R},p_{S},j_{S},n}^{\left(D\right)}=\sum_{m_{T}=1}^{M_{T}}\sum_{m_{R}=1}^{M_{R}}\sum_{m_{S}=1}^{M_{S}}\sum_{r=1}^{R}h_{m_{D},f,m_{T}}^{\left(RD\right)}c_{m_{T},f,p_{R},j_{R},m_{R}}^{\left(R\right)}h_{m_{R},f,m_{S}}^{\left(SR\right)}c_{m_{S},f,p_{S},j_{S},r}^{\left(S\right)}s_{n,r}.$$

**Figure 15:**
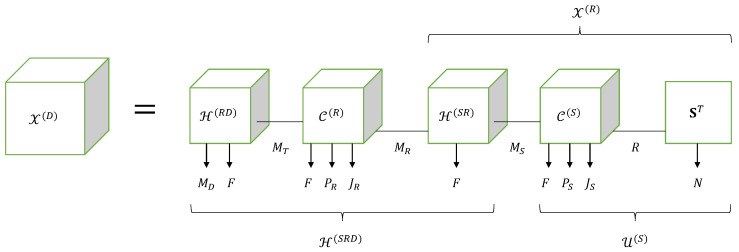
Tucker train model of a two-hop relay system using TSTF codings.

Note that, in Equation (106), the operation $\times_{3}^{1}$ and the first operation $\times_{5}^{1}$ correspond to contractions along the modes $m_{T}$ and $m_{R}$, respectively, while the second operation $\times_{5}^{1}$ and the mode-7 product correspond to contractions along the modes $m_{S}$ and *r*, respectively, as shown in Equation (107). From this equation, we conclude that the tensor $X^{\left(D\right)}$ satisfies the Tucker train (TT) model $〚H^{\left(RD\right)},C^{\left(R\right)},H^{\left(SR\right)},C^{\left(S\right)},S;M_{T},M_{R},M_{S},R〛$, as represented by means of the block diagram in Figure 15.

Comparing Equation (106) with the equation of the NGTD-7 model presented in Table 17, we deduce the following correspondences:

(108) $$\begin{array}{cc} \left(H^{\left(RD\right)},C^{\left(R\right)},H^{\left(SR\right)},C^{\left(S\right)},S\right) & ⟷\left(A^{\left(1\right)},G^{\left(1\right)},U,G^{\left(2\right)},A^{\left(2\right)}\right) \end{array}$$

(109) $$\begin{array}{cc} \left(M_{D},F,P_{R},J_{R},P_{S},J_{S},N,M_{T},M_{R},M_{S},R\right) & ⟷\left(I_{1},I_{2},I_{3},I_{4},I_{5},I_{6},I_{7},R_{1},R_{2},R_{3},R_{4}\right). \end{array}$$

Now, let us define the effective channel tensor $H^{\left(SRD\right)}\in C^{M_{D}\times F\times P_{R}\times J_{R}\times M_{S}}$ between the source and the destination, as follows:

(110) $$H^{\left(SRD\right)}=H^{\left(RD\right)}\times_{3}^{1}C^{\left(R\right)}\times_{5}^{1}H^{\left(SR\right)}=C^{\left(R\right)}\times_{1}^{3}H^{\left(RD\right)}\times_{5}^{1}H^{\left(SR\right)}.$$

This tensor satisfies a GTD-(2,5) model whose core tensor is the relay-coding tensor $C^{\left(R\right)}$. The expressions (105) and (106) of $X^{\left(D\right)}$ can then be rewritten in terms of the effective channel as follows:

(111) $$\begin{array}{cc} X^{\left(D\right)}= & \;H^{\left(SRD\right)}\times_{5}^{1}U^{\left(S\right)} \end{array}$$

(112) $$\begin{array}{cc} \;= & \;H^{\left(SRD\right)}\times_{5}^{1}C^{\left(S\right)}\times_{7}S=C^{\left(S\right)}\times_{1}^{5}H^{\left(SRD\right)}\times_{7}S. \end{array}$$

From Equation (111), we conclude that the TT structure of the tensor $X^{\left(D\right)}$ can be interpreted as the contraction of the tensors $H^{\left(SRD\right)}$ and $U^{\left(S\right)}$ along the mode $m_{S}$, as illustrated in Figure 15.

Moreover, a comparison with the NGTD-7 model reveals that $X^{\left(D\right)}$ is the nesting of the tensor $H^{\left(SRD\right)}$, corresponding to the component $X^{\left(1\right)}$ defined in (45), with the tensor $X^{\left(R\right)}$ defined in (101) and to be associated with the component $X^{\left(2\right)}$ in (46), in red and blue in Equations (106) and (107), respectively, sharing the tensor factor $H^{\left(SR\right)}$ (in green).

In Section 6, under the assumption that coding tensors are known at the destination, the coupled GTD-(2,5) models (112) and (110) will be exploited to design a KronF-based semi-blind receiver composed of two stages; in the first one, the symbol matrix and the effective channel tensor are jointly estimated, applying the KronF algorithm to the model (112), and in the second one, individual channel tensors $\left(H^{\left(RD\right)},H^{\left(SR\right)}\right)$ are estimated using the KronF algorithm applied to model (110) of the effective channel estimated in the previous stage. See Table 28.

The TSTF system presented above is the most comprehensive relay system utilizing tensor codings that combine signal diversities across space (antennas), frequency (subcarriers), time (time-spreading lengths and symbol periods), and code (chips) domains, corresponding to the indices $\left(m_{D},f,p_{R},j_{R},p_{S},j_{S},n\right)$ of the received signals’ tensor $X^{\left(D\right)}$. This system extends the relay system proposed in [41], which does not take frequency and chip diversities into account, inducing third-order instead of fifth-order coding tensors, and it considers flat-fading channels, leading to a NTD-4 model for the fourth-order tensor of signals received at the destination instead of a NGTD-7 model for the TSTF system. The coding in [41], denoted as STST, as well as the tensor-based codings TST and STSTF, can be derived from the TSTF coding by applying certain simplifications, as detailed hereafter.

#### 5.3.2. TST Coding

With TST coding, we consider flat fading channels $H^{\left(SR\right)}\in C^{M_{R}\times M_{S}}$ and $H^{\left(RD\right)}\in C^{M_{D}\times M_{T}}$, and we assume that both the source and relay transmit signals using only one subcarrier. This implies removing frequency diversity from coding tensors, resulting in fourth-order tensors $C^{\left(S\right)}\in C^{M_{S}\times P_{S}\times J_{S}\times R}$ and $C^{\left(R\right)}\in C^{M_{T}\times P_{R}\times J_{R}\times M_{R}}$. The signals received at the destination then form a sixth-order tensor, $X^{\left(D\right)}\in C^{M_{D}\times P_{R}\times J_{R}\times P_{S}\times J_{S}\times N}$, directly deduced from Equation (107), which is simplified as follows:

(113) $$x_{m_{D},p_{R},j_{R},p_{S},j_{S},n}^{\left(D\right)}=\sum_{m_{T}=1}^{M_{T}}\sum_{m_{R}=1}^{M_{R}}\sum_{m_{S}=1}^{M_{S}}\sum_{r=1}^{R}h_{m_{D},m_{T}}^{\left(RD\right)}c_{m_{T},p_{R},j_{R},m_{R}}^{\left(R\right)}h_{m_{R},m_{S}}^{\left(SR\right)}c_{m_{S},p_{S},j_{S},r}^{\left(S\right)}s_{n,r}.$$

The tensor $X^{\left(D\right)}$ satisfies a NTD-6 model, such as that introduced in Table 17, corresponding to the GTTD-(2,4,2,4,2) model $〚H^{\left(RD\right)},C^{\left(R\right)},H^{\left(SR\right)},C^{\left(S\right)},S;M_{T},M_{R},M_{S},R〛$, which can be viewed as the nesting of the following two TD-(2,4) models deduced from Equations (110) and (101), sharing the common matrix factor $H^{\left(SR\right)}$:

(114) $$\begin{array}{cc} H^{\left(SRD\right)} & =C^{\left(R\right)}\times_{1}H^{\left(RD\right)}\times_{4}{\left(H^{\left(SR\right)}\right)}^{T}\in C^{M_{D}\times P_{R}\times J_{R}\times M_{S}} \end{array}$$

(115) $$\begin{array}{cc} X^{\left(R\right)} & =C^{\left(S\right)}\times_{1}H^{\left(SR\right)}\times_{4}S\in C^{M_{R}\times P_{S}\times J_{S}\times N}. \end{array}$$

**Figure 16:**
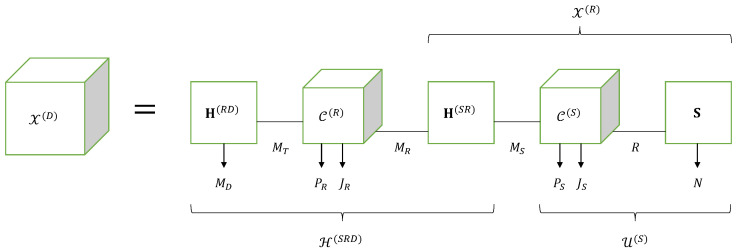
Tucker train model of a two-hop relay system using TST codings.

$H^{\left(SRD\right)}$ and $X^{\left(R\right)}$ are the tensors of the effective channel and of signals received at the relay, respectively. Figure 16 depicts a block diagram of the TST system, highlighting its nesting structure. Equations (114) and (115) are analogous to Equations (43) and (44) with the following correspondences:

(116) $$\begin{array}{cc} \left(H^{\left(RD\right)},C^{\left(R\right)},H^{\left(SR\right)},C^{\left(S\right)},S\right) & ⟷\left(A^{\left(1\right)},G^{\left(1\right)},U,G^{\left(2\right)},A^{\left(2\right)}\right) \end{array}$$

(117) $$\begin{array}{cc} \left(M_{D},P_{R},J_{R},P_{S},J_{S},N,M_{T},M_{R},M_{S},R\right) & ⟷\left(I_{1},I_{2},I_{3},I_{4},I_{5},I_{6},R_{1},R_{2},R_{3},R_{4}\right). \end{array}$$

Moreover, Equation (112) becomes the following:

(118) $$X^{\left(D\right)}=C^{\left(S\right)}\times_{1}^{4}H^{\left(SRD\right)}\times_{6}S.$$

In the next section, the coupled TD-(2,4) models (114) and (118) will be used to design a KronF-based semi-blind receiver. See Table 28.

#### 5.3.3. STSTF and STST Codings

Simplified versions of the TSTF and TST codings, denoted as STSTF and STST, are now introduced. These coding schemes do not consider chip diversity for STSTF, and both chip and frequency diversities for STST, leading to fourth-order coding tensors $C^{\left(S\right)}\in C^{M_{S}\times F\times P_{S}\times R}$, $C^{\left(R\right)}\in C^{M_{T}\times F\times P_{R}\times M_{R}}$, and third-order tensors $C^{\left(S\right)}\in C^{M_{S}\times P_{S}\times R}$, $C^{\left(R\right)}\in C^{M_{T}\times P_{R}\times M_{R}}$, respectively. With these simplifications, we can directly infer the signals received at the destination from Equations (107) and (113):

(119) $$x_{m_{D},f,p_{R},p_{S},n}^{\left(D\right)}=\sum_{m_{T}=1}^{M_{T}}\sum_{m_{R}=1}^{M_{R}}\sum_{m_{S}=1}^{M_{S}}\sum_{r=1}^{R}h_{m_{D},f,m_{T}}^{\left(RD\right)}c_{m_{T},f,p_{R},m_{R}}^{\left(R\right)}h_{m_{R},f,m_{S}}^{\left(SR\right)}c_{m_{S},f,p_{S},r}^{\left(S\right)}s_{n,r}$$

for the STSTF system, and

(120) $$x_{m_{D},p_{R},p_{S},n}^{\left(D\right)}=\sum_{m_{T}=1}^{M_{T}}\sum_{m_{R}=1}^{M_{R}}\sum_{m_{S}=1}^{M_{S}}\sum_{r=1}^{R}h_{m_{D},m_{T}}^{\left(RD\right)}c_{m_{T},p_{R},m_{R}}^{\left(R\right)}h_{m_{R},m_{S}}^{\left(SR\right)}c_{m_{S},p_{S},r}^{\left(S\right)}s_{n,r}$$

for the STST one. The received signals in (119) and (120) define fifth- and fourth-order tensors that satisfy, respectively, the NGTD-5 and NTD-4 models, such as those introduced in Section 3.3.3 and Section 3.3.2, respectively.

**Remark 8.**  *Note that, for STST, the tensors $H^{\left(SRD\right)}$ and $X^{\left(R\right)}$ are modeled by means of the following TD-(2,3) models, directly deduced from (114) and (115):*

(121) $$\begin{array}{cc} H^{\left(SRD\right)} & =C^{\left(R\right)}\times_{1}H^{\left(RD\right)}\times_{3}{\left(H^{\left(SR\right)}\right)}^{T}\in C^{M_{D}\times P_{R}\times M_{S}} \end{array}$$

(122) $$\begin{array}{cc} X^{\left(R\right)} & =C^{\left(S\right)}\times_{1}H^{\left(SR\right)}\times_{3}S\in C^{M_{R}\times P_{S}\times N}. \end{array}$$

In summary, the signals received at the destination for a TSTF system form a seventh-order tensor $X^{\left(D\right)}\in C^{M_{D}\times F\times P_{R}\times J_{R}\times P_{S}\times J_{S}\times N}$, satisfying a NGTD-7 model, i.e., a generalized Tucker train decomposition, GTTD-(3,5,3,5,2). The TST, STSTF, and STST systems are particular cases of TSTF, with certain signal diversities omitted (frequency for TST, chip for STSTF, and both for STST). These systems satisfy Tucker train models previously defined as GTTD-(2,4,2,4,2), GTTD-(3,4,3,4,2), and TTD-(2,3,2,3,2), corresponding to NTD-6, NGTD-5, and NTD-4 models, respectively. Note that the STST system corresponds to the relay system proposed in [41], while the other three are new.

### 5.4. AF Two-Hop Relay Systems Using Matrix-Based Codings

Coding schemes based on Khatri–Rao products provide a less complex coding structure than tensor codings. Following the same approach as in Section 5.3.1, let us start by describing the general DKRSTF coding based on a double Khatri–Rao product to introduce signal diversities in the space, time, and frequency domains.

#### 5.4.1. DKRSTF Coding

This coding was introduced in [39] as an OFDM extension of the Khatri–Rao ST coding (KRST) defined in [99], in the context of a point-to-point communication system. Here, we propose using DKRSTF in a two-hop relay system. As introduced in Table 23, the coding matrices $C^{\left(S\right)}\in C^{P_{S}\times M_{S}}$ and $C^{\left(R\right)}\in C^{P_{R}\times M_{R}}$ spread the transmitted symbols across $P_{S}$ and $P_{R}$ time blocks, while the matrices $A^{\left(S\right)}\in C^{F\times R}$, $W^{\left(S\right)}\in C^{M_{S}\times R}$ and $W^{\left(R\right)}\in C^{M_{T}\times M_{R}}$ are employed to add frequency and space diversities. Table 24 summarizes the equations of the two-hop system using DKRSTF coding.

The first Khatri–Rao product provides space-frequency pre-coded signals at the source, forming the third-order tensor $V^{\left(S\right)}\in C^{M_{S}\times F\times N}$, such as:

(123) $$V_{FN\times M_{S}}^{\left(S\right)}=\left(A^{\left(S\right)}⋄S\right){W^{\left(S\right)}}^{T}\;\;↔\;\;V^{\left(S\right)}=\;I_{R}\times_{1}W^{\left(S\right)}\times_{2}A^{\left(S\right)}\times_{3}S.$$

This tensor $V^{\left(S\right)}$ satisfies the CPD model, $〚W^{\left(S\right)},A^{\left(S\right)},S;R〛$, which can be written in scalar notation as follows:

(124) $$v_{m_{S},f,n}^{\left(S\right)}=\sum_{r=1}^{R}w_{m_{S},r}^{\left(S\right)}a_{f,r}^{\left(S\right)}s_{n,r}.$$

Then, the pre-coded signals are spread over $P_{S}$ blocks using a second Khatri–Rao ST coding matrix $C^{\left(S\right)}$ to give the fourth-order tensor $U^{\left(S\right)}\in C^{M_{S}\times P_{S}\times F\times N}$ of space–time-frequency encoded signals, such as:

(125) $$U_{P_{S}FN\times M_{S}}^{\left(S\right)}=C^{\left(S\right)}⋄V_{FN\times M_{S}}^{\left(S\right)}\;\;↔\;\;U_{c}^{\left(S\right)}=I_{M_{S}}\times_{1}I_{M_{S}}\times_{2}C^{\left(S\right)}\times_{3}V_{FN\times M_{S}}^{\left(S\right)},$$

where $U_{c}^{\left(S\right)}\in C^{M_{S}\times P_{S}\times FN}$ denotes a contracted form of $U^{\left(S\right)}$, resulting from a combination of the modes (f,n). In scalar form, we have:

(126) $$u_{m_{S},p_{S},f,n}^{\left(S\right)}=c_{p_{S},m_{S}}^{\left(S\right)}v_{m_{S},f,n}^{\left(S\right)}.$$

After space-time-frequency coding at the source and transmission through the channel $H^{\left(SR\right)}\in C^{M_{R}\times M_{S}}$, the signals received at the relay form a fourth-order tensor $X^{\left(R\right)}\in C^{M_{R}\times P_{S}\times F\times N}$ given by:

(127) $$X^{\left(R\right)}=U^{\left(S\right)}\times_{1}H^{\left(SR\right)}\;\;↔\;\;x_{m_{R},p_{S},f,n}^{\left(R\right)}=\sum_{m_{S}=1}^{M_{S}}h_{m_{R},m_{S}}^{\left(SR\right)}u_{m_{S},p_{S},f,n}^{\left(S\right)}.$$

Replacing $U^{\left(S\right)}$ with its expression (125) gives the following contracted form $X_{c}^{\left(R\right)}\in C^{M_{R}\times P_{S}\times FN}$ of $X^{\left(R\right)}$:

(128) $$X_{c}^{\left(R\right)}=U_{c}^{\left(S\right)}\times_{1}H^{\left(SR\right)}=I_{M_{S}}\times_{1}H^{\left(SR\right)}\times_{2}C^{\left(S\right)}\times_{3}V_{FN\times M_{S}}^{\left(S\right)},$$

or, in scalar form,

(129) $$x_{m_{R},p_{S},f,n}^{\left(R\right)}=\sum_{m_{S}=1}^{M_{S}}h_{m_{R},m_{S}}^{\left(SR\right)}c_{p_{S},m_{S}}^{\left(S\right)}v_{m_{S},f,n}^{\left(S\right)}.$$

Combining (129) with (124) gives:

(130) $$x_{m_{R},p_{S},f,n}^{\left(R\right)}=\sum_{m_{S}=1}^{M_{S}}\sum_{r=1}^{R}h_{m_{R},m_{S}}^{\left(SR\right)}c_{p_{S},m_{S}}^{\left(S\right)}w_{m_{S},r}^{\left(S\right)}a_{f,r}^{\left(S\right)}s_{n,r}.$$

Equations (123) and (128) are to be compared to Equations (20) and (25) associated with an NCDP-4 model. From this comparison, we conclude that the tensor $X^{\left(R\right)}$ satisfies the NCDP-4 model $〚H^{\left(SR\right)},C^{\left(S\right)},W^{\left(S\right)},A^{\left(S\right)},S;M_{S},R〛$ with the following correspondences:

(131) $$\begin{array}{cc} \left(H^{\left(SR\right)},C^{\left(S\right)},W^{\left(S\right)},A^{\left(S\right)},S\right) & ⟷\left(A^{\left(1\right)},B^{\left(1\right)},G,A^{\left(2\right)},B^{\left(2\right)}\right) \end{array}$$

(132) $$\begin{array}{cc} \left(M_{R},P_{S},F,N,M_{S},R\right) & ⟷\left(I_{1},I_{2},I_{3},I_{4},R_{1},R_{2}\right). \end{array}$$

Due to the AF protocol, the signals received at the relay are encoded using a Khatri–Rao space-time coding with matrices $W^{\left(R\right)}$ and $C^{\left(R\right)}$ to give the fifth-order tensor $U^{\left(R\right)}\in C^{M_{T}\times P_{R}\times P_{S}\times F\times N}$ of coded signals defined by the following equation:

(133) $$U_{P_{R}P_{S}FN\times M_{T}}^{\left(R\right)}=\left(C^{\left(R\right)}⋄X_{P_{S}FN\times M_{R}}^{\left(R\right)}\right){W^{\left(R\right)}}^{T}\;\;↔\;\;U_{c}^{\left(R\right)}=\;I_{M_{R}}\times_{1}W^{\left(R\right)}\times_{2}C^{\left(R\right)}\times_{3}X_{P_{S}FN\times M_{R}}^{\left(R\right)},$$

where $U_{c}^{\left(R\right)}\in C^{M_{T}\times P_{R}\times P_{S}FN}$ denotes a contracted form of $U^{\left(R\right)}$, resulting from a combination of the modes $\left(p_{S},f,n\right)$. After transmission through the channel $H^{\left(RD\right)}\in C^{M_{D}\times M_{T}}$, the signals received at the destination form a fifth-order tensor $X^{\left(D\right)}\in C^{M_{D}\times P_{R}\times P_{S}\times F\times N}$ whose a contracted form is given by:

(134) $$X_{c}^{\left(D\right)}=U_{c}^{\left(R\right)}\times_{1}H^{\left(RD\right)}\;\;↔\;\;X_{M_{D}\times P_{R}P_{S}FN}^{\left(D\right)}=H^{\left(RD\right)}U_{M_{T}\times P_{R}P_{S}FN}^{\left(R\right)}.$$

Combining Equations (134) and (133) gives:

(135) $$X_{c}^{\left(D\right)}=I_{M_{R}}\times_{1}\left(H^{\left(RD\right)}W^{\left(R\right)}\right)\times_{2}C^{\left(R\right)}\times_{3}X_{P_{S}FN\times M_{R}}^{\left(R\right)}.$$

When $M_{R}>M_{T}$, the term $H^{\left(RD\right)}W^{\left(R\right)}\in C^{M_{D}\times M_{R}}$ can be interpreted as a virtual antenna array at the destination due to a virtual increase in the number of transmit antennas at the relay, thanks to the space coding matrix $W^{\left(R\right)}$. Note that a necessary identifiability condition (184) with the KRF receiver for the DKRSTF system is $M_{R}\geq M_{T}$.

Let us define the virtual channel as follows:

(136) $$B≜H^{\left(RD\right)}W^{\left(R\right)}\;\;↔\;\;b_{m_{D},m_{R}}=\sum_{m_{T}=1}^{M_{T}}h_{m_{D},m_{T}}^{\left(RD\right)}w_{m_{T},m_{R}}^{\left(R\right)}.$$

**Figure 17:**
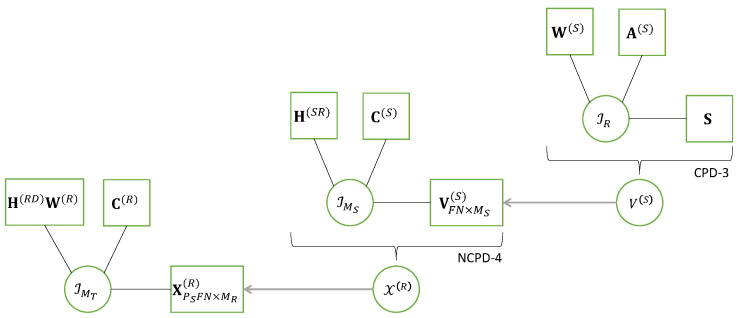
NCPD-5 model for the DKRSTF system as a cascade of three CPD-3 models.

Then, Equation (135) defines the CPD-3 model $〚B,C^{\left(R\right)},X_{P_{S}FN\times M_{R}}^{\left(R\right)};M_{R}〛$. From this equation, we conclude that the tensor $X^{\left(D\right)}$ satisfies a NCPD-5 model, corresponding to the cascade of the CPD-3 model (135) with the NCPD-4 model (130) of the tensor $X^{\left(R\right)}$, which is itself the cascade of two CPD-3 models, as illustrated in the block diagram in Figure 17.

When the CPD-3 model (135) and the expression (129) of $x_{m_{R},f,p_{S},n}^{\left(R\right)}$ are taken into account, the tensor of signals received at the destination can also be written in scalar form as follows:

(137) $$\begin{array}{cc} x_{m_{D},p_{R},p_{S},f,n}^{\left(D\right)}= & \sum_{m_{R}=1}^{M_{R}}b_{m_{D},m_{R}}c_{p_{R},m_{R}}^{\left(R\right)}x_{m_{R},p_{S},f,n}^{\left(R\right)} \end{array}$$

(138) $$\begin{array}{cc} \;= & \sum_{m_{R}=1}^{M_{R}}\sum_{m_{S}=1}^{M_{S}}b_{m_{D},m_{R}}c_{p_{R},m_{R}}^{\left(R\right)}h_{m_{R},m_{S}}^{\left(SR\right)}c_{p_{S},m_{S}}^{\left(S\right)}v_{m_{S},f,n}^{\left(S\right)} \end{array}$$

(139) $$\begin{array}{cc} \;= & \sum_{m_{S}=1}^{M_{S}}h_{m_{D},p_{R},m_{S}}^{\left(SRD\right)}c_{p_{S},m_{S}}^{\left(S\right)}v_{m_{S},f,n}^{\left(S\right)}, \end{array}$$

where $H^{\left(SRD\right)}\in C^{M_{D}\times P_{R}\times M_{S}}$ is the effective channel between the source and the destination, defined as follows:

(140) $$h_{m_{D},p_{R},m_{S}}^{\left(SRD\right)}=\sum_{m_{R}=1}^{M_{R}}b_{m_{D},m_{R}}c_{p_{R},m_{R}}^{\left(R\right)}h_{m_{R},m_{S}}^{\left(SR\right)}$$

and $v_{m_{S},f,n}^{\left(S\right)}$ is defined in (124). In Section 6.4, the three CPD-3 models (123), (139), and (140), with the definition (136) of B, will be exploited to devise a KRF-based, closed-form receiver, allowing for the joint estimation of the three unknown matrices $\left(S,H^{\left(SR\right)},H^{\left(RD\right)}\right)$.

**Remark 9.**  *From Equations (124), (136), and (138), the signals received at the destination can be written as follows:*

(141) $$x_{m_{D},p_{R},p_{S},f,n}^{\left(D\right)}=\sum_{m_{T}=1}^{M_{T}}\sum_{m_{R}=1}^{M_{R}}\sum_{m_{S}=1}^{M_{S}}\sum_{r=1}^{R}h_{m_{D},m_{T}}^{\left(RD\right)}w_{m_{T},m_{R}}^{\left(R\right)}c_{p_{R},m_{R}}^{\left(R\right)}h_{m_{R},m_{S}}^{\left(SR\right)}c_{p_{S},m_{S}}^{\left(S\right)}w_{m_{S},r}^{\left(S\right)}a_{f,r}^{\left(S\right)}s_{n,r}.$$

*Comparing this equation with (119), we deduce that the DKRSTF system can be viewed as a particular case of STSTF when considering flat-fading channels, with the following choices for the coding tensors:*

(142) $$c_{m_{S},f,p_{S},r}^{\left(S\right)}=c_{p_{S},m_{S}}^{\left(S\right)}w_{m_{S},r}^{\left(S\right)}a_{f,r}^{\left(S\right)}\;\;\mathrm{and}\;\;c_{m_{T},f,p_{R},m_{R}}^{\left(R\right)}=w_{m_{T},m_{R}}^{\left(R\right)}c_{p_{R},m_{R}}^{\left(R\right)}.$$

*Note that, in the case of DKRSTF, the frequency diversity is omitted from the coding at the relay.*

#### 5.4.2. SKRST Coding

Now, we consider a simplified version of KRST coding, denoted as SKRST, introduced in [40,53] for two-hop relay systems. At the source, the coding consists of a simple Khatri–Rao product using the coding matrix $C^{\left(S\right)}\in C^{P_{S}\times M_{S}}$, with $S\in C^{N\times M_{S}}$, resulting in the following encoded signals tensor $U^{\left(S\right)}\in C^{M_{S}\times P_{S}\times N}$:

(143) $$U^{\left(S\right)}=I_{M_{S}}\times_{1}I_{M_{S}}\times_{2}C^{\left(S\right)}\times_{3}S\;\;↔\;\;U_{P_{S}N\times M_{S}}^{\left(S\right)}=C^{\left(S\right)}⋄S,$$

or, in scalar form,

(144) $$u_{m_{S},p_{S},n}^{\left(S\right)}=c_{p_{S},m_{S}}^{\left(S\right)}s_{n,m_{S}}.$$

Without the frequency-space coding matrices $\left(A^{\left(S\right)},W^{\left(S\right)},W^{\left(R\right)}\right)$, Equations (130), (137) and (135) of the signals received at the relay and the destination are then simplified as follows:

(145) $$\begin{array}{cc} x_{m_{R},p_{S},n}^{\left(R\right)} & =\sum_{m_{S}=1}^{M_{S}}h_{m_{R},m_{S}}^{\left(SR\right)}c_{p_{S},m_{S}}^{\left(S\right)}s_{n,m_{S}}⟷X^{\left(R\right)}=I_{M_{S}}\times_{1}H^{\left(SR\right)}\times_{2}C^{\left(S\right)}\times_{3}S \end{array}$$

(146) $$\begin{array}{cc} x_{m_{D},p_{R},p_{S},n}^{\left(D\right)} & =\sum_{m_{R}=1}^{M_{R}}h_{m_{D},m_{R}}^{\left(RD\right)}c_{p_{R},m_{R}}^{\left(R\right)}x_{m_{R},p_{S},n}^{\left(R\right)}⟷X_{c}^{\left(D\right)}=I_{M_{R}}\times_{1}H^{\left(RD\right)}\times_{2}C^{\left(R\right)}\times_{3}X_{P_{S}N\times M_{R}}^{\left(R\right)}, \end{array}$$

with $X_{c}^{\left(D\right)}\in C^{M_{D}\times P_{R}\times P_{S}N}$. Note that this simplification implies a constraint on the relay, such that the numbers of transmit and receive antennas must be equal ($M_{T}=M_{R}$). The coupled Equations (145) and (146), to be compared to Equations (20) and (25), define a NCPD-4 model for the fourth-order tensor $X^{\left(D\right)}\in C^{M_{D}\times P_{R}\times P_{S}\times N}$, which can be written in the following scalar form:

(147) $$x_{m_{D},p_{R},p_{S},n}^{\left(D\right)}=\sum_{m_{R}=1}^{M_{R}}\sum_{m_{S}=1}^{M_{S}}h_{m_{D},m_{R}}^{\left(RD\right)}c_{p_{R},m_{R}}^{\left(R\right)}h_{m_{R},m_{S}}^{\left(SR\right)}c_{p_{S},m_{S}}^{\left(S\right)}s_{n,m_{S}},$$

with the following correspondences:

(148) $$\begin{array}{cc} \left(H^{\left(RD\right)},C^{\left(R\right)},H^{\left(SR\right)},C^{\left(S\right)},S\right) & ⟷\left(A^{\left(1\right)},B^{\left(1\right)},G,A^{\left(2\right)},B^{\left(2\right)}\right) \end{array}$$

(149) $$\begin{array}{cc} \left(M_{D},P_{R},P_{S},N,M_{R},M_{S}\right) & ⟷\left(I_{1},I_{2},I_{3},I_{4},R_{1},R_{2}\right). \end{array}$$

**Figure 18:**
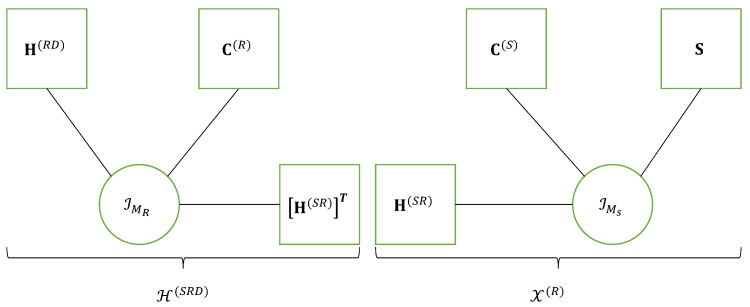
NCPD-4 model for the SKRST system.

Let us define the effective channel tensor $H^{\left(SRD\right)}\in C^{M_{D}\times P_{R}\times M_{S}}$ as follows:

(150) $$H^{\left(SRD\right)}=I_{M_{R}}\times_{1}H^{\left(RD\right)}\times_{2}C^{\left(R\right)}\times_{3}{\left(H^{\left(SR\right)}\right)}^{T},$$

which satisfies the CPD-3 model $〚H^{\left(RD\right)},C^{\left(R\right)},{H^{\left(SR\right)}}^{T};M_{R}〛$ and corresponds to Equation (19). Noting that the tensor $X^{\left(R\right)}$ of signals received at the relay, defined in (145), satisfies the CPD-3 model $〚H^{\left(SR\right)},C^{\left(S\right)},S;M_{S}〛$ sharing the common factor $H^{\left(SR\right)}$ with $H^{\left(SRD\right)}$, we conclude that the NCPD-4 model of $X^{\left(D\right)}$ is associated with the nesting of the CPD-3 models of $H^{\left(SRD\right)}$ and $X^{\left(R\right)}$, as illustrated with the block diagram in Figure 18. The correspondences (148) and (149) will be employed to design ALS- and KRF-based receivers for the SKRST system. See Table 28.

### 5.5. DF Two-Hop Relay Systems Using STST-MSMKron and SKRST-MSMKR Codings

In the preceding two sections, as well as in most of the systems presented in Table 22, the AF protocol was employed. A significant improvement in the symbol error rate (SER) can be obtained with the DF protocol at the cost of additional computational complexity at the relay. In [55], the authors propose two-hop relay systems using multiple Khatri–Rao product-based space-time (MKRST) and multiple Kronecker product-based space-time (MKronST) codings at the source and relay, with a comparison of AF, DF, and estimate-forward (EF) protocols.

In this section, we consider two-hop relay systems with a DF protocol, using STST and SKRST codings, respectively, combined with multiple symbol matrices Kronecker and Khatri–Rao products, which simplify the MKronST and MKRST codings in eliminating the pre-coding matrix. These combined codings, respectively denoted as STST-MSMKron and SKRST-MSMKR, were originally proposed in [97,98], in the context of point-to-point systems. Here, we extend their use to a cooperative scenario.

Let us consider *Q* symbol matrices $S^{\left(q\right)}$, with q∈〈Q〉, to be transmitted via the source. With MSMKron and MSMKR codings, each symbol $s_{i,j}^{\left(q\right)}$ of a given symbol matrix $S^{\left(q\right)}$ is duplicated via Kronecker (Kron) and Khatri–Rao (KR) products with other symbol matrices $S^{\left(q^{\prime }\right)}$, where $q^{\prime }\neq q$. These multiple Kron and KR products induce a mutual spreading of transmitted symbols, thus providing additional diversity. Below, we detail these two systems.

#### 5.5.1. STST-MSMKron Coding

For the STST-MSMKron coding, the symbol matrices $S^{\left(q\right)}\in C^{N_{q}\times R_{q}}$ are encoded at the source using the (Q+2)-order ST coding tensor $C^{\left(S\right)}\in C^{M_{S}\times P_{S}\times R_{1}\times \cdots \times R_{Q}}$, leading to the following (Q+2)-order tensor $U^{\left(S\right)}\in C^{M_{S}\times P_{S}\times N_{1}\times \cdots \times N_{Q}}$ of signals to be transmitted:

(151) $$U^{\left(S\right)}=C^{\left(S\right)}\times_{1}I_{M_{S}}\times_{2}I_{P_{S}}\times_{3}S^{\left(1\right)}\times_{4}\cdots \times_{Q+2}S^{\left(Q\right)},$$

which can be represented in a compact form as $U_{c}^{\left(S\right)}=C^{\left(S\right)}\times_{3}S\in C^{M_{S}\times P_{S}\times N}$, with $S=\overset{Q}{\underset{q=1}{⊗}}S^{\left(q\right)}\in C^{N\times R}$, $N=\prod_{q=1}^{Q}N_{q}$ and $R=\prod_{q=1}^{Q}R_{q}$. In a scalar format, the coded signals transmitted via the $m_{S}$th antenna, during the $p_{S}$th time slot, are given by:

(152) $$u_{m_{S},p_{S},n_{1},\cdots ,n_{Q}}^{\left(S\right)}=\sum_{r_{1}=1}^{R_{1}}\cdots \sum_{r_{Q}=1}^{R_{Q}}c_{m_{S},p_{S},r_{1},\cdots ,r_{Q}}^{\left(S\right)}\prod_{q=1}^{Q}s_{n_{q},r_{q}}^{\left(q\right)}.$$

After transmission through the channel $H^{\left(SR\right)}\in C^{M_{R}\times M_{S}}$, the signals received at the relay form the (Q+2)-order tensor $X^{\left(R\right)}\in C^{M_{R}\times P_{S}\times N_{1}\times \cdots \times N_{Q}}$ given by:

(153) $$\begin{array}{cc} X^{\left(R\right)} & =U^{\left(S\right)}\times_{1}H^{\left(SR\right)}\\ \; & =C^{\left(S\right)}\times_{1}H^{\left(SR\right)}\times_{2}I_{P_{S}}\times_{3}S^{\left(1\right)}\times_{4}\cdots \times_{Q+2}S^{\left(Q\right)}. \end{array}$$

Note that the mode-2 product with identity matrix $I_{P_{S}}$ can be interpreted as repeating the transmission of the same symbols during $P_{S}$ time slots. The DF protocol, used at the relay, involves estimating the information symbols and then re-encoding the estimated symbols using the tensor $C^{\left(R\right)}\in C^{M_{S}\times P_{R}\times R_{1}\times \cdots \times R_{Q}}$ before transmission to the destination. After transmission through the channel $H^{\left(RD\right)}\in C^{M_{D}\times M_{S}}$ of the signals coded at the relay, the tensor structure (153) is repeated at the destination for the tensor $X^{\left(D\right)}\in C^{M_{D}\times P_{R}\times N_{1}\times \cdots \times N_{Q}}$, given by:

(154) $$\begin{array}{c} X^{\left(D\right)}=C^{\left(R\right)}\times_{1}H^{\left(RD\right)}\times_{3}{\hat{S}}^{\left(1\right)}\times_{4}\cdots \times_{Q+2}{\hat{S}}^{\left(Q\right)}, \end{array}$$

where ${\hat{S}}^{\left(q\right)}$ denotes the estimate of $S^{\left(q\right)}$ obtained via the decoding process at the relay.

Due to the DF protocol, the equations describing the signals received at the relay and the destination are similar, and they are written in scalar form as follows:

(155) $$\begin{array}{cc} x_{m_{R},p_{S},n_{1},...,n_{Q}}^{\left(R\right)} & =\sum_{m_{S}=1}^{M_{S}}\sum_{r_{1}=1}^{R_{1}}\cdots \sum_{r_{Q}=1}^{R_{Q}}h_{m_{R},m_{S}}^{\left(SR\right)}c_{m_{S},p_{S},r_{1},...,r_{Q}}^{\left(S\right)}\prod_{q=1}^{Q}s_{n_{q},r_{q}}^{\left(q\right)} \end{array}$$

(156) $$\begin{array}{cc} x_{m_{D},p_{R},n_{1},...,n_{Q}}^{\left(D\right)} & =\sum_{m_{S}=1}^{M_{S}}\sum_{r_{1}=1}^{R_{1}}\cdots \sum_{r_{Q}=1}^{R_{Q}}h_{m_{D},m_{S}}^{\left(RD\right)}c_{m_{S},p_{R},r_{1},...,r_{Q}}^{\left(R\right)}\prod_{q=1}^{Q}{\hat{s}}_{n_{q},r_{q}}^{\left(q\right)}. \end{array}$$

#### 5.5.2. SKRST-MSMKR Coding

For the SKRST-MSMKR coding, the symbol matrices $S^{\left(q\right)}\in C^{N_{q}\times M_{S}}$ are encoded at the source using the coding matrix $C^{\left(S\right)}\in C^{P_{S}\times M_{S}}$, giving a (Q+2)-order tensor $U^{\left(S\right)}\in C^{M_{S}\times P_{S}\times N_{1}\times \cdots \times N_{Q}}$ for the encoded signals, defined by means of the following matrix unfolding:

(157) $$U_{P_{S}N_{1}\cdots N_{Q}\times M_{S}}^{\left(S\right)}=C^{\left(S\right)}⋄S^{\left(1\right)}⋄\cdots ⋄S^{\left(Q\right)}\;\;↔\;\;U^{\left(S\right)}=I_{M_{S}}\times_{1}I_{M_{S}}\times_{2}C^{\left(S\right)}\times_{3}S^{\left(1\right)}\cdots \times_{Q+2}S^{\left(Q\right)},$$

which can be compactly written as $U_{P_{S}N\times M_{S}}^{\left(S\right)}=C^{\left(S\right)}⋄S$, with $S=\overset{Q}{\underset{q=1}{⋄}}S^{\left(q\right)}\in C^{N\times M_{S}}$ and $N=\prod_{q=1}^{Q}N_{q}$. The coded signals to be transmitted by the $m_{S}$th antenna, during the $p_{S}$th time slot, are given by:

(158) $$u_{m_{S},p_{S},n_{1},...,n_{Q}}^{\left(S\right)}=c_{p_{S},m_{S}}^{\left(S\right)}\prod_{q=1}^{Q}s_{n_{q},m_{S}}^{\left(q\right)}.$$

After transmission through the channel $H^{\left(SR\right)}$, the signals received at the relay form a (Q+2)-order tensor $X^{\left(R\right)}\in C^{M_{R}\times P_{S}\times N_{1}\times \cdots \times N_{Q}}$ given by:

(159) $$X^{\left(R\right)}=U^{\left(S\right)}\times_{1}H^{\left(SR\right)}\;\;↔\;\;X_{M_{R}\times P_{S}N_{1}\cdots N_{Q}}^{\left(R\right)}=H^{\left(SR\right)}U_{M_{S}\times P_{S}N_{1}\cdots N_{Q}}^{\left(S\right)}.$$

Replacing $U_{M_{S}\times P_{S}N_{1}\cdots N_{Q}}^{\left(S\right)}$ with its expression (157) gives:

(160) $$X_{M_{R}\times P_{S}N_{1}\cdots N_{Q}}^{\left(R\right)}=H^{\left(SR\right)}{\left(C^{\left(S\right)}⋄S^{\left(1\right)}⋄\cdots ⋄S^{\left(Q\right)}\right)}^{T}.$$

The tensor $X^{\left(R\right)}$ satisfies the CPD-(Q+2) model $〚H^{\left(SR\right)},C^{\left(S\right)},S^{\left(1\right)},...,S^{\left(Q\right)};M_{S}〛$. With the DF protocol, the symbol matrices are estimated at the relay and then re-encoded with the coding matrix $C^{\left(R\right)}\in C^{P_{R}\times M_{S}}$ before transmission to the destination. The tensor $X^{\left(D\right)}\in C^{M_{D}\times P_{R}\times N_{1}\times \cdots \times N_{Q}}$ of signals received at the destination satisfies the CPD-(Q+2) model $〚H^{\left(RD\right)},C^{\left(R\right)},{\hat{S}}^{\left(1\right)},...,{\hat{S}}^{\left(Q\right)};M_{S}〛$, given by:

(161) $$X_{M_{D}\times P_{R}N_{1}\cdots N_{Q}}^{\left(D\right)}=H^{\left(RD\right)}{\left(C^{\left(R\right)}⋄{\hat{S}}^{\left(1\right)}⋄\cdots ⋄{\hat{S}}^{\left(Q\right)}\right)}^{T}.$$

Note that this equation implies that $M_{T}=M_{S}$. The signals received at the relay and the destination are given in scalar format as follows:

(162) $$\begin{array}{cc} x_{m_{R},p_{S},n_{1},...,n_{Q}}^{\left(R\right)} & =\sum_{m_{S}=1}^{M_{S}}h_{m_{R},m_{S}}^{\left(SR\right)}c_{p_{S},m_{S}}^{\left(S\right)}\prod_{q=1}^{Q}s_{n_{q},m_{S}}^{\left(q\right)} \end{array}$$

(163) $$\begin{array}{cc} x_{m_{D},p_{R},n_{1},...,n_{Q}}^{\left(D\right)} & =\sum_{m_{S}=1}^{M_{S}}h_{m_{D},m_{S}}^{\left(RD\right)}c_{p_{R},m_{S}}^{\left(R\right)}\prod_{q=1}^{Q}{\hat{s}}_{n_{q},m_{S}}^{\left(q\right)}. \end{array}$$

It is worth mentioning that STST and SKSRT systems with a DF protocol can be deduced easily from STST-MSMKron and SKRST-MSMKR systems by considering only one matrix symbol, i.e., Q=1. This configuration will be considered in the experimental study, in Section 7.

Table 25 summarizes the tensors of signals encoded at the source and of signals received at the relay and the destination for each two-hop relay system considered in this overview, unifying their tensor representation and mentioning the tensor model of $X^{\left(D\right)}$.

**Remark 10.**  *Analyzing the nested models in Table 25 leads to the following conclusions:*

- *In the case of tensor-based codings, the nested models of signals received at the relay and the destination result from the nesting of two TD-(2,4) and two TD-(2,3) models for the TST and STST systems, respectively, and two GTD-(2,5) and two GTD-(2,4) models for the TSTF and STSTF systems, respectively, with coding tensors as core tensors.*
- *In the case of matrix-based codings, the nested models are cascades of three and two CPD-3 models for the DKRSTF and SKRST systems, respectively, with a coding matrix as factor of each CPD-3 model.*
- *In the case of combined codings, with the DF protocol, the signals received at relay and destination form (Q+2)-order tensor models which satisfy a CPD model with a coding matrix as a factor for the SKRST-MSMKR system, and a TD model with a coding tensor as a core tensor for the STST-MSMKron system.*

Capitalizing on these tensor models and assuming the coding matrices/tensors are known at relay and destination allow closed-form, semi-blind receivers to be devised for all the considered relay systems, as shown in the next section. Moreover, knowledge of coding matrices/tensors guarantees the uniqueness of the tensor models. In Section 7, extensive Monte Carlo simulation results will be presented to compare the performance of the different relay systems and associated semi-blind receivers.

## 6. Semi-Blind Receivers

In the following, we assume that the coding matrices and tensors used at the source and relay are known at the destination. In Section 4, two families of algorithms were presented to estimate unknown parameters in various NCPD and NTD models, namely ALS and closed-form algorithms. In this section, these algorithms are used to design the following semi-blind receivers for relay systems modeled by means of nested tensor models:

- KronF receiver for the TSTF system (Table 21, for a NGTD-7 model);
- KronF receiver for the TST system (Table 20, for a NTD-6 model);
- KronF receiver for the STSTF system (Remark 7, for a NGTD-5 model);
- KronF receiver for the STST system (Table 19, for a NTD-4 model);
- ALS receiver for the STST system (Equations (82)–(84), for a NTD-4 model);
- KRF receiver for the SKRST system (Table 18, for a NCPD-4 model);
- ALS receiver for the SKRST system (Equations (67), (68) and (70), for a NCPD-4 model).

In Table 26, we provide the correspondences between the tensors $X^{\left(D\right)}$ of signals received at the destination in systems TSTF/TST/STSTF/STST/SKRST with the generic nested tensor models described in Section 3.

Note that the DKRSTF, STST-MSMKron, and SKRST-MSMKR systems that satisfy specific tensor models will be considered separately in Section 6.4, Section 6.5 and Section 6.6, respectively. To facilitate the understanding of the receivers presented in a unified way, in Table 28, we first detail the development of the KronF receiver for the TSTF system represented by a NGTD-7 model, which is the most general one. An analysis of the parametric complexity of the proposed receivers is presented in Appendix D.

### 6.1. KronF Receiver for the TSTF System

In Table 21, two closed-form algorithms derived from two different ways of combining the matrix unfoldings (49)–(52) of the NGTD-7 model are proposed. Each algorithm is composed of two steps conditioning the order of estimation of the unknown factors $\left(A^{\left(1\right)},U,A^{\left(2\right)}\right)$. In the context of a relay system, the closed-form algorithm 1 allows for first estimating the matrix factor $A^{\left(2\right)}$, corresponding to the symbol matrix S, while algorithm 2 first estimates the tensor factor $A^{\left(1\right)}$ which corresponds to the channel tensor $H^{\left(RD\right)}$. Note that these two closed-form algorithms can be used in parallel in order to eliminate propagation errors in the estimation of these two factors. In the next subsection, such a strategy will be exploited to devise closed-form receivers for the STST and SKRST systems. Now, let us detail the two-step KronF receiver for the TSTF system, derived from the coupled GTD-(2,5) models (112) and (110).

Using the correspondences in Table 26, Equation (52) gives the following block-diagonal matrix unfolding of $X^{\left(D\right)}$ deduced from the GTD-(2,5) model (112):

(164) $${\mathrm{bdiag}}_{f}\left(X_{NM_{D}P_{R}J_{R}\times P_{S}J_{S}}^{\left(D\right)}\left(f\right)\right)=\left[S⊗{\mathrm{bdiag}}_{f}\left(H_{M_{D}P_{R}J_{R}\times M_{S}}^{\left(SRD\right)}\left(f\right)\right)\right]{\mathrm{bdiag}}_{f}\left(C_{RM_{S}\times P_{S}J_{S}}^{\left(S\right)}\left(f\right)\right).$$

The LS estimate of the KronP in this equation is given by:

(165) $$S⊗{\mathrm{bdiag}}_{f}\left(H_{M_{D}P_{R}J_{R}\times M_{S}}^{\left(SRD\right)}\left(f\right)\right)={\mathrm{bdiag}}_{f}\left(X_{NM_{D}P_{R}J_{R}\times P_{S}J_{S}}^{\left(D\right)}\left(f\right)\right){\mathrm{bdiag}}_{f}{\left(C_{RM_{S}\times P_{S}J_{S}}^{\left(S\right)}\left(f\right)\right)}^{†}.$$

The factors S and $H^{\left(SRD\right)}$ are then estimated by applying the KronF method.

After reshaping the estimate ${\mathrm{bdiag}}_{f}\left({\hat{H}}_{M_{D}P_{R}J_{R}\times M_{S}}^{\left(SRD\right)}\left(f\right)\right)$ into ${\mathrm{bdiag}}_{f}\left({\hat{H}}_{M_{D}M_{S}\times P_{R}J_{R}}^{\left(SRD\right)}\left(f\right)\right)$, in a second step, we use the following unfolding deduced from the GTD-(2,5) model (110) of $H^{\left(SRD\right)}$ to estimate the individual channels:

(166) $${\mathrm{bdiag}}_{f}\left(H_{M_{D}M_{S}\times P_{R}J_{R}}^{\left(SRD\right)}\left(f\right)\right)={\mathrm{bdiag}}_{f}\left(H_{M_{D}\times M_{T}}^{\left(RD\right)}\left(f\right)⊗H_{M_{S}\times M_{R}}^{\left(SR\right)}\left(f\right)\right){\mathrm{bdiag}}_{f}\left(C_{M_{T}M_{R}\times P_{R}J_{R}}^{\left(R\right)}\left(f\right)\right),$$

which corresponds to the unfolding (49), leading to the following LS estimate of the KronP:

(167) $${\mathrm{bdiag}}_{f}\left(H_{M_{D}\times M_{T}}^{\left(RD\right)}\left(f\right)⊗H_{M_{S}\times M_{R}}^{\left(SR\right)}\left(f\right)\right)={\mathrm{bdiag}}_{f}\left({\hat{H}}_{M_{D}M_{S}\times P_{R}J_{R}}^{\left(SRD\right)}\left(f\right)\right){\mathrm{bdiag}}_{f}{\left(C_{M_{T}M_{R}\times P_{R}J_{R}}^{\left(R\right)}\left(f\right)\right)}^{†}.$$

The KronF method is applied once again to obtain estimates of the individual channel tensors $H^{\left(SR\right)}$ and $H^{\left(RD\right)}$.

KronF receivers for other tensor-based coding systems can easily be derived using the same approach, with simplified unfoldings. For instance, in the case of the TST system, where frequency diversity is not considered, the simplification involves fixing F=1. Similarly, for the STSTF system which does not consider chip diversity, the simplification results from choosing $J_{S}=J_{R}=1$.

For each two-step KronF receiver, the procedure consists of (i) jointly estimating $\left(S,H^{\left(SRD\right)}\right)$ using the expression of the received signal tensor $X^{\left(D\right)}$ in terms of the effective channel and (ii) estimating the individual channels using the estimated effective channel. Table 28 presents the unfoldings used for each system to derive KronF receivers.

**Remark 11.**  The matrix Moore–Penrose pseudo-inverses to be calculated with the proposed closed-form receivers can be simplified when choosing orthonormal matrices. For instance, let us consider the case of a column-orthonormal matrix, $A\in C^{M\times N}$, i.e., $A^{H}A=I_{N}$, which implies r(A)=N and M≥N. Its left-inverse is then given by $A^{†}=A^{H}$. Similarly, if A is row-orthonormal, i.e., $AA^{H}=I_{M}$, implying r(A)=M and N≥M, its right-inverse is also given by $A^{†}=A^{H}$. In the case of the pseudo-inversion of $A^{T}$, we have ${\left(A^{T}\right)}^{†}=A^{*}$ if $A^{T}$ is column- or row-orthonormal.

*In Table 28, this property is used to simplify the calculation of right-inverses of matrix unfoldings of coding tensors, chosen row-orthonormal by construction.*

*The same simplification is valid for the left-inverse of the KRP and KronP of two column-orthonormal matrices. Indeed, for $A\in C^{M\times N}$, $B\in C^{P\times N}$ and $C\in C^{P\times Q}$, we have:*

(168) $$\begin{array}{cc} {\left(A⋄B\right)}^{H}\left(A⋄B\right)= & \;A^{H}A⊙B^{H}B=I_{N}⊙I_{N}=I_{N} \end{array}$$

(169) $$\begin{array}{cc} {\left(A⊗C\right)}^{H}\left(A⊗C\right)= & \;A^{H}A⊗C^{H}C=I_{N}⊗I_{Q}=I_{NQ}, \end{array}$$

*which implies ${\left(A⋄B\right)}^{†}={\left(A⋄B\right)}^{H}$ and ${\left(A⊗C\right)}^{†}={\left(A⊗C\right)}^{H}$. This property is used to simplify the calculation of the left-inverse of the KRP $\left(A^{\left(S\right)}⋄W^{\left(S\right)}\right)$ of coding matrices in the KRF receiver of the DKRSTF system.*

### 6.2. KronF Receiver for the STST System and KRF Receiver for the SKRST System

Closed-form receivers for the STST and SKRST systems are derived from Table 19 and Table 18, respectively, by exploiting the correspondences in Table 26, with $\left(H^{\left(SRD\right)},X^{\left(R\right)}\right)$ playing the role of $\left(X^{\left(1\right)},X^{\left(2\right)}\right)$. This gives the two closed-form receivers summarized in Table 27.

Closed-form receiver 1 will be taken as the standard algorithm, allowing for the estimation of the symbol matrix in the first step and thus avoiding error propagation in the information symbol recovery. As ${\hat{H}}^{\left(SR\right)}$ and ${\hat{H}}^{\left(RD\right)}$ are calculated, in the second step, from the estimate ${\hat{H}}_{M_{D}M_{S}\times P_{R}}^{\left(SRD\right)}$, this introduces error propagation in channel estimates.

To eliminate this error propagation in the estimation of $H^{\left(RD\right)}$, an alternative solution consists of using both closed-form algorithms in parallel.

The channel $H^{\left(SR\right)}$ can then be estimated either from the estimate ${\hat{H}}_{M_{D}P_{R}\times M_{S}}^{\left(SRD\right)}$ obtained with Algorithm 1, or the estimate ${\hat{X}}_{P_{S}N\times M_{R}}^{\left(R\right)}$ obtained with Algorithm 2. For SKRST, using mode-3 and mode-1 unfoldings of the CPD-3 models (150) of $H^{\left(SRD\right)}$ and (145) of $X^{\left(R\right)}$, respectively, we derive the following two solutions to estimate $H^{\left(SR\right)}$:

(170) $${\hat{H}}^{\left(SR\right)}={\left({\hat{H}}^{\left(RD\right)}⋄C^{\left(R\right)}\right)}^{†}{\hat{H}}_{M_{D}P_{R}\times M_{S}}^{\left(SRD\right)}\;\;\mathrm{and}\;\;{\left({\hat{H}}^{\left(SR\right)}\right)}^{T}={\left(C^{\left(S\right)}⋄\hat{S}\right)}^{†}{\hat{X}}_{P_{S}N\times M_{R}}^{\left(R\right)}.$$

Similarly, for STST, from unfoldings of the TD-(2,3) models (121) and (122), we obtain:

(171) $$\begin{array}{ccc} {\hat{H}}^{\left(SR\right)} & = & {\left(\left({\hat{H}}^{\left(RD\right)}⊗I_{P_{R}}\right)C_{M_{T}P_{R}\times M_{R}}^{\left(R\right)}\right)}^{†}{\hat{H}}_{M_{D}P_{R}\times M_{S}}^{\left(SRD\right)}\\ \; & \mathrm{and} & \\ \;{\left({\hat{H}}^{\left(SR\right)}\right)}^{T} & = & {\left(\left(I_{P_{S}}⊗\hat{S}\right)C_{P_{S}R\times M_{S}}^{\left(S\right)}\right)}^{†}{\hat{X}}_{P_{S}N\times M_{R}}^{\left(R\right)}. \end{array}$$

In Section 7, these alternative solutions will be compared with the closed-form receivers 1.

Necessary identifiability conditions for the algorithms presented in Table 27 are linked with the uniqueness of right inverses of the coding matrices/unfoldings, which gives:

(172) $$P_{S}\geq M_{S}\;\;,\;\;P_{R}\geq M_{R}$$

for the KRF receiver of the SKRST system, and

(173) $$P_{S}\geq M_{S}R\;\;,\;\;P_{R}\geq M_{R}M_{T}$$

for the KronF receiver of the STST system.

For the alternative solutions proposed above, the additional steps (170) and (171) to estimate $H^{\left(SR\right)}$ imply the following supplementary identifiability conditions: $M_{D}P_{R}\geq M_{R}$ and $P_{S}N\geq M_{S}$. Note that these conditions are always satisfied when (172) and (173) are satisfied.

### 6.3. ALS Receivers for the STST and SKRST Systems

To derive ALS-based receivers, we use the unfoldings (82)–(84) for the STST system and (67), (68) and (70) for the SKRST system, with the correspondences in Table 26, to estimate the symbol matrix and the channels in an alternate and iterative way. Equations for the ALS-based receivers are presented in Table 28.

### 6.4. KRF Receiver for the DKRSTF System

Recall the CPD-3 models (139), (140) and (123), with the definition (136), used to design the KRF-based receiver for the DKRSTF system:

(174) $$\begin{array}{cc} X_{c}^{\left(D\right)}= & \;I_{M_{S}}\times_{1}H_{M_{D}P_{R}\times M_{S}}^{\left(SRD\right)}\times_{2}C^{\left(S\right)}\times_{3}V_{FN\times M_{S}}^{\left(S\right)} \end{array}$$

(175) $$\begin{array}{cc} H^{\left(SRD\right)}= & \;I_{M_{R}}\times_{1}B\times_{2}C^{\left(R\right)}\times_{3}{\left(H^{\left(SR\right)}\right)}^{T} \end{array}$$

(176) $$\begin{array}{cc} V^{\left(S\right)}= & \;I_{R}\times_{1}W^{\left(S\right)}\times_{2}A^{\left(S\right)}\times_{3}S \end{array}$$

(177) $$\begin{array}{cc} B= & \;H^{\left(RD\right)}W^{\left(R\right)}, \end{array}$$

where $X_{c}^{\left(D\right)}\in C^{M_{D}P_{R}\times P_{S}\times FN}$ is a contracted form of $X^{\left(D\right)}\in C^{M_{D}\times P_{R}\times P_{S}\times F\times N}$, and $H_{M_{D}P_{R}\times M_{S}}^{\left(SRD\right)}$ and $V_{FN\times M_{S}}^{\left(S\right)}$ are matrix unfoldings of the tensors $H^{\left(SRD\right)}$ and $V^{\left(S\right)}$. From the CPD models (174) and (175), we obtain the following tall mode-2 unfoldings:

(178) $$\begin{array}{cc} X_{FNM_{D}P_{R}\times P_{S}}^{\left(D\right)} & =\left(V_{FN\times M_{S}}^{\left(S\right)}⋄H_{M_{D}P_{R}\times M_{S}}^{\left(SRD\right)}\right){C^{\left(S\right)}}^{T} \end{array}$$

(179) $$\begin{array}{cc} H_{M_{D}M_{S}\times P_{R}}^{\left(SRD\right)} & =\left(B⋄{H^{\left(SR\right)}}^{T}\right){C^{\left(R\right)}}^{T}. \end{array}$$

The above unfoldings are alternately exploited in a two-step KRF-based algorithm. In the first step, the LS estimate of the KRP in (178) is calculated, and its factors are estimated using the KRF method:

(180) $$V_{FN\times M_{S}}^{\left(S\right)}⋄H_{M_{D}P_{R}\times M_{S}}^{\left(SRD\right)}=X_{FNM_{D}P_{R}\times P_{S}}^{\left(D\right)}{\left({C^{\left(S\right)}}^{T}\right)}^{†}\;\;\overset{\mathrm{KRF}}{\Rightarrow }\;\;\left({\hat{V}}_{FN\times M_{S}}^{\left(S\right)},{\hat{H}}_{M_{D}P_{R}\times M_{S}}^{\left(SRD\right)}\right).$$

In the second step, the estimate ${\hat{H}}_{M_{D}P_{R}\times M_{S}}^{\left(SRD\right)}$ is reshaped into ${\hat{H}}_{M_{D}M_{S}\times P_{R}}^{\left(SRD\right)}$, and then the LS estimate of the KRP in (179) is calculated. The KRF method is applied again to estimate its factors:

(181) $$B⋄{H^{\left(SR\right)}}^{T}={\hat{H}}_{M_{D}M_{S}\times P_{R}}^{\left(SRD\right)}{\left({C^{\left(R\right)}}^{T}\right)}^{†}\;\;\overset{\mathrm{KRF}}{\Rightarrow }\;\;\left(\hat{B},{\hat{H}}^{\left(SR\right)}\right).$$

Finally, after reshaping ${\hat{V}}_{FN\times M_{S}}^{\left(S\right)}$ into ${\hat{V}}_{FM_{S}\times N}^{\left(S\right)}$, LS estimates of S and $H^{\left(RD\right)}$ are obtained using the following unfolding deduced from the CPD-3 model (176):

(182) $$V_{FM_{S}\times N}^{\left(S\right)}=\left(A^{\left(S\right)}⋄W^{\left(S\right)}\right)S^{T},$$

and the definition (177) of B, which gives:

(183) $${\hat{S}}^{T}={\left(A^{\left(S\right)}⋄W^{\left(S\right)}\right)}^{†}{\hat{V}}_{FM_{S}\times N}^{\left(S\right)}\;\;\mathrm{and}\;\;{\hat{H}}^{\left(RD\right)}=\hat{B}{\left(W^{\left(R\right)}\right)}^{†}.$$

Necessary identifiability conditions are linked to the uniqueness of LS estimates in (180)–(183), i.e., full column-rank conditions for $C^{\left(S\right)}$, $C^{\left(R\right)}$, $A^{\left(S\right)}⋄W^{\left(S\right)}$, and ${\left(W^{\left(R\right)}\right)}^{T}$, which gives:

(184) $$P_{S}\geq M_{S}\;\;,\;\;P_{R}\geq M_{R}\;\;,\;\;FM_{S}\geq R\;\;,\;\;M_{R}\geq M_{T}.$$

### 6.5. KronF Receiver for the STST-MSMKron System

Since the DF protocol is employed for this system, the signals received at the relay are processed to estimate the symbol matrices before re-encoding and forwarding to the destination. From the TD-(Q+2) model (153), we deduce the following tall mode-2 unfolding of the tensor $X^{\left(R\right)}\in C^{M_{R}\times P_{S}\times N_{1}\times \cdots \times N_{Q}}$ of signals received at the relay:

(185) $$X_{N_{1}\cdots N_{Q}M_{R}\times P_{S}}^{\left(R\right)}=\left(\overset{Q}{\underset{q=1}{⊗}}S^{\left(q\right)}⊗H^{\left(SR\right)}\right)C_{R_{1}\cdots R_{Q}M_{S}\times P_{S}}^{\left(S\right)}.$$

Analogously, from the TD model (154), we deduce the following tall mode-2 unfolding of the tensor $X^{\left(D\right)}\in C^{M_{D}\times P_{R}\times N_{1}\times \cdots \times N_{Q}}$:

(186) $$X_{N_{1}\cdots N_{Q}M_{D}\times P_{R}}^{\left(D\right)}=\left(\overset{Q}{\underset{q=1}{⊗}}{\hat{S}}^{\left(q\right)}⊗H^{\left(RD\right)}\right)C_{R_{1}\cdots R_{Q}M_{S}\times P_{R}}^{\left(R\right)}.$$

Equations (185) and (186) are exploited by a KronF-based receiver, at the relay and the destination, to estimate the symbol matrices ${\hat{S}}^{\left(q\right)}$ and ${\hat{\hat{S}}}^{\left(q\right)}$, q∈〈Q〉, simultaneously with the channel matrices ${\hat{H}}^{\left(SR\right)}$ and ${\hat{H}}^{\left(RD\right)}$, using the THOSVD algorithm:

(187) $$\begin{array}{cc} \overset{Q}{\underset{q=1}{⊗}}S^{\left(q\right)}⊗H^{\left(SR\right)}=X_{N_{1}\cdots N_{Q}M_{R}\times P_{S}}^{\left(R\right)}{\left(C_{R_{1}\cdots R_{Q}M_{S}\times P_{S}}^{\left(S\right)}\right)}^{†}\;\; & \overset{\mathrm{KronF}}{\Rightarrow }\;\;\left({\hat{S}}^{\left(1\right)},\cdots ,{\hat{S}}^{\left(Q\right)},{\hat{H}}^{\left(SR\right)}\right), \end{array}$$

(188) $$\begin{array}{cc} \overset{Q}{\underset{q=1}{⊗}}{\hat{S}}^{\left(q\right)}⊗H^{\left(RD\right)}=X_{N_{1}\cdots N_{Q}M_{D}\times P_{R}}^{\left(D\right)}{\left(C_{R_{1}\cdots R_{Q}M_{S}\times P_{R}}^{\left(R\right)}\right)}^{†}\;\; & \overset{\mathrm{KronF}}{\Rightarrow }\;\;\left({\hat{\hat{S}}}^{\left(1\right)},\cdots ,{\hat{\hat{S}}}^{\left(Q\right)},{\hat{H}}^{\left(RD\right)}\right). \end{array}$$

Note that the symbol matrices estimated at the relay and the destination are denoted ${\hat{S}}^{\left(q\right)}$ and ${\hat{\hat{S}}}^{\left(q\right)}$, respectively. It is worth mentioning that, with the DF protocol, the destination node estimates only the RD channel, while the SR channel is estimated at the relay.

### 6.6. KRF Receiver for the SKRST-MSMKR System

Similarly to the STST-MSMKron system, the SKRST-MSMKR one employs the DF protocol, and the proposed KRF receiver follows the same steps as previously described for the KronF receiver. Thus, from the CPD-(Q+2) models (160) and (161), we deduce the following tall mode-2 unfoldings of the tensors $X^{\left(R\right)}$ and $X^{\left(D\right)}$:

(189) $$\begin{array}{cc} X_{N_{1}\cdots N_{Q}M_{R}\times P_{S}}^{\left(R\right)} & =\left(\overset{Q}{\underset{q=1}{⋄}}S^{\left(q\right)}⋄H^{\left(SR\right)}\right){C^{\left(S\right)}}^{T}, \end{array}$$

(190) $$\begin{array}{cc} X_{N_{1}\cdots N_{Q}M_{D}\times P_{R}}^{\left(D\right)} & =\left(\overset{Q}{\underset{q=1}{⋄}}{\hat{S}}^{\left(q\right)}⋄H^{\left(RD\right)}\right){C^{\left(R\right)}}^{T}. \end{array}$$

with $C^{\left(S\right)}\in C^{P_{S}\times M_{S}}$ and $C^{\left(R\right)}\in C^{P_{R}\times M_{S}}$. Note that these equations imply $M_{S}=M_{T}$, which means that the source and the relay must have the same number of transmit antennas.

Equations (189) and (190) are exploited by a KRF-based receiver, at the relay and the destination, to estimate the symbol matrices ${\hat{S}}^{\left(q\right)}$ and ${\hat{\hat{S}}}^{\left(q\right)}$, q∈〈Q〉, with the channel matrices ${\hat{H}}^{\left(SR\right)}$ and ${\hat{H}}^{\left(RD\right)}$, using the THOSVD algorithm:

(191) $$\begin{array}{cc} \overset{Q}{\underset{q=1}{⋄}}S^{\left(q\right)}⋄H^{\left(SR\right)}=X_{N_{1}\cdots N_{Q}M_{R}\times P_{S}}^{\left(R\right)}{\left({C^{\left(S\right)}}^{T}\right)}^{†}\;\; & \overset{\mathrm{KRF}}{\Rightarrow }\;\;\left({\hat{S}}^{\left(1\right)},\cdots ,{\hat{S}}^{\left(Q\right)},{\hat{H}}^{\left(SR\right)}\right), \end{array}$$

(192) $$\begin{array}{cc} \overset{Q}{\underset{q=1}{⋄}}{\hat{S}}^{\left(q\right)}⋄H^{\left(RD\right)}=X_{N_{1}\cdots N_{Q}M_{D}\times P_{R}}^{\left(D\right)}{\left({C^{\left(R\right)}}^{T}\right)}^{†}\;\; & \overset{\mathrm{KRF}}{\Rightarrow }\;\;\left({\hat{\hat{S}}}^{\left(1\right)},\cdots ,{\hat{\hat{S}}}^{\left(Q\right)},{\hat{H}}^{\left(RD\right)}\right). \end{array}$$

As for the STST-MSMKron system, the destination node estimates only the RD channel, while the SR channel is estimated at the relay.

**Remark 12.**  *The necessary identifiability conditions for the KronF and KRF receivers (187)–(188) and (191)–(192) are linked to the uniqueness of LS estimates in these equations, which respectively gives:*

(193) $$P_{S}\geq M_{S}\prod_{q=1}^{Q}R_{q}\;\;,\;\;P_{R}\geq M_{T}\prod_{q=1}^{Q}R_{q},$$

*and*

(194) $$P_{S}\geq M_{S}\;\;,\;\;P_{R}\geq M_{S}.$$

From the conditions (193) and (194), we conclude that the SKRST-MSMKR system is less constraining for choosing the time-spreading lengths than the STST-MSMKron system.

The unfoldings used to derive the proposed semi-blind receivers for all considered relay systems, as well as the factors estimated at each step, are summarized in Table 28. The correspondences with equations and algorithms associated with the generic nested tensor models are also given.

### 6.7. Zero-Forcing Receivers for the STST and SKRST Systems

In Section 7, the proposed ALS and closed-form receivers of the STST and SKRST systems will be compared to zero-forcing (ZF) receivers which assume a perfect knowledge of communication channels at the destination node. These receivers can be directly derived from the unfoldings of the received signals tensor used to estimate the symbol matrix with the ALS receivers.

Thus, by exploiting the correspondences in Table 26, the ZF receiver for the STST system is deduced from (82) as follows:

(195) $${\hat{S}}^{T}={\left[\left(I_{P_{S}}⊗\left(H^{\left(RD\right)}⊗I_{P_{R}}\right)C_{M_{T}P_{R}\times M_{R}}^{\left(R\right)}H^{\left(SR\right)}\right)C_{P_{S}M_{S}\times R}^{\left(S\right)}\right]}^{†}X_{P_{S}M_{D}P_{R}\times N}^{\left(D\right)}.$$

Similarly, the ZF receiver for the SKRST system, deduced from (68), is given by:

(196) $${\hat{S}}^{T}={\left[C^{\left(S\right)}⋄\left(H^{\left(RD\right)}⋄C^{\left(R\right)}\right)H^{\left(SR\right)}\right]}^{†}X_{P_{S}M_{D}P_{R}\times N}^{\left(D\right)}.$$

The SER obtained with ZF receivers serves as a reference to evaluate the proposed semi-blind receivers. ZF receivers are also used to analyze the impact of the choice of some design parameters on the SER performance in the ideal situation where the channels are perfectly known.

### 6.8. Ambiguity Relations, Identifiability Conditions, and Transmission Rates

Factors estimated using KRF and KronF methods are obtained up to scalar ambiguities, as discussed in Appendix B and Appendix C. To eliminate these ambiguities, some a priori knowledge is required.

For KronF receivers, the knowledge of only one element of one of the matrices involved in each KronP is enough. Thus, for the KronF receiver of the TSTF system, based on KronPs in (165) and (167), we assume the knowledge of the elements $s_{1,1}$ and $h_{1,f,1}^{\left(RD\right)}$ of S and $H_{\cdot f\cdot }^{\left(RD\right)}$, for f∈〈F〉. This a priori knowledge is used to deduce the scaling factors allowing to eliminate the scaling ambiguities as follows:

(197) $$\begin{array}{ccc} \lambda_{S}=s_{1,1}/{\hat{s}}_{1,1}\;; & & \;\lambda_{H_{\cdot f\cdot }^{\left(RD\right)}}=h_{1,f,1}^{\left(RD\right)}/{\hat{h}}_{1,f,1}^{\left(RD\right)}\;\;,\;\;\mathrm{for}\;\;f\in \langle F\rangle ,\\ \hat{S}\leftarrow \lambda_{S}\;\hat{S}\;; & & \;\;\;{\hat{H}}_{FM_{D}P_{R}J_{R}\times FM_{S}}^{\left(SRD\right)}\leftarrow \lambda_{S}^{-1}\;{\hat{H}}_{FM_{D}P_{R}J_{R}\times FM_{S}}^{\left(SRD\right)}\;;\\ {\hat{H}}_{\cdot f\cdot }^{\left(RD\right)}\leftarrow \lambda_{H_{\cdot f\cdot }^{\left(RD\right)}}\;{\hat{H}}_{\cdot f\cdot }^{\left(RD\right)}\;, & & \;\;\;{\hat{H}}_{\cdot f\cdot }^{\left(SR\right)}\leftarrow \lambda_{H_{\cdot f\cdot }^{\left(RD\right)}}^{-1}\;{\hat{H}}_{\cdot f\cdot }^{\left(SR\right)}, \end{array}$$

with $H_{FM_{D}P_{R}J_{R}\times FM_{S}}^{\left(SRD\right)}={\mathrm{bdiag}}_{f}\left(H_{M_{D}P_{R}J_{R}\times M_{S}}^{\left(SRD\right)}\left(f\right)\right)$. The same procedure is applied to the receivers of the TST, STSTF, and STST systems, noting that the TST and STST systems do not exploit frequency diversity, and therefore, F=1 in these cases.

For KRF receivers, a priori knowledge of one row of one of the matrices involved in each KRP is required. For instance, in the case of the DKRSTF system based on KRPs in (180) and (181), the first rows of $V_{FN\times M_{S}}^{\left(S\right)}$ and B can be calculated using the first rows of S and $H^{\left(RD\right)}$, respectively, from the unfolding (123) of $V^{\left(S\right)}$ and the definition (177) of B, as follows:

(198) $${\left[V_{FN\times M_{S}}^{\left(S\right)}\right]}_{1,\cdot }=\left(a_{1,\cdot }^{\left(S\right)}⋄s_{1,\cdot }\right){W^{\left(S\right)}}^{T}\;\;\mathrm{and}\;\;b_{1,\cdot }=h_{1,\cdot }^{\left(RD\right)}W^{\left(R\right)}.$$

The ambiguity relations are then given by the following:

(199) $$\begin{array}{ccc} \Lambda_{V^{\left(S\right)}}=\mathrm{diag}\left(\left.{\left[V_{FN\times M_{S}}^{\left(S\right)}\right]}_{1,\cdot }\right.\right){\left[\mathrm{diag}\left(\left.{\left[{\hat{V}}_{FN\times M_{S}}^{\left(S\right)}\right]}_{1,\cdot }\right.\right)\right]}^{-1}\;; & & \;\Lambda_{B}=\mathrm{diag}\left(b_{1,\cdot }\right){\left[\mathrm{diag}({\hat{b}}_{1,\cdot })\right]}^{-1}\\ {\hat{V}}_{FN\times M_{S}}^{\left(S\right)}\leftarrow {\hat{V}}_{FN\times M_{S}}^{\left(S\right)}\Lambda_{V^{\left(S\right)}}\;; & & \;\;\;{\hat{H}}_{M_{D}P_{R}\times M_{S}}^{\left(SRD\right)}\leftarrow {\hat{H}}_{M_{D}P_{R}\times M_{S}}^{\left(SRD\right)}\Lambda_{V^{\left(S\right)}}^{-1}\\ \hat{B}\leftarrow \hat{B}\Lambda_{B}\;; & & \;\;\;{\left.{\hat{H}}^{\left(SR\right)}\right.}^{T}\leftarrow {\left.{\hat{H}}^{\left(SR\right)}\right.}^{T}\Lambda_{B}^{-1}. \end{array}$$

For the KRF receiver of the SKRST system, we have the following ambiguity relations:

(200) $$\begin{array}{ccc} \Lambda_{S}=\mathrm{diag}\left(s_{1,\cdot }\right){\left[\mathrm{diag}({\hat{s}}_{1,\cdot })\right]}^{-1}\;; & & \;\Lambda_{H^{\left(RD\right)}}=\mathrm{diag}\left(h_{1,\cdot }^{\left(RD\right)}\right){\left[\mathrm{diag}({\hat{h}}_{1,\cdot }^{\left(RD\right)})\right]}^{-1},\\ \hat{S}\leftarrow \hat{S}\Lambda_{S}\;; & & \;\;\;{\hat{H}}_{M_{D}P_{R}\times M_{S}}^{\left(SRD\right)}\leftarrow {\hat{H}}_{M_{D}P_{R}\times M_{S}}^{\left(SRD\right)}\Lambda_{S}^{-1},\\ {\hat{H}}^{\left(RD\right)}\leftarrow {\hat{H}}^{\left(RD\right)}\Lambda_{H^{\left(RD\right)}}\;; & & \;\;\;{\left.{\hat{H}}^{\left(SR\right)}\right.}^{T}\leftarrow {\left.{\hat{H}}^{\left(SR\right)}\right.}^{T}\Lambda_{H^{\left(RD\right)}}^{-1}. \end{array}$$

For combined codings based on multiple KronPs and KRPs, the closed-form receivers are described by Equations (187) and (188) for the STST-MSMKron system and (191)–(192) for the SKRST-MSMKR one. Due to the DF protocol, each estimation step at the relay and destination requires a priori knowledge of one element or one row of each symbol matrix to remove ambiguities. In this case, the channels $H^{\left(SR\right)}$ and $H^{\left(RD\right)}$ are estimated blindly, that is to say without a priori knowledge on $H^{\left(RD\right)}$, unlike the AF protocol.

Finally, for the ALS receivers of the SKRST and STST systems, the procedure to eliminate the ambiguities results from the discussion in Section 3.3.1 and Section 3.3.2 on the uniqueness of the NCDP-4 and NTD-4 models. The fact that the coding matrices/tensors are assumed to be perfectly known at the destination ensures the uniqueness of the tensor models and avoids permutation ambiguity in estimated factor matrices. Only scaling ambiguities have to be removed. By exploiting the ambiguity relations (32) and (42) and the correspondences in Table 26, we deduce the following ambiguity relations for the ALS receivers of the SKRST and STST systems:

(201) $$\begin{array}{cc} \mathrm{NCPD}\text{-}4:\;\; & \Lambda_{S}=\mathrm{diag}\left(s_{1,\cdot }\right){\left[\mathrm{diag}({\hat{s}}_{1,\cdot })\right]}^{-1}\;\;\mathrm{and}\;\;\Lambda_{H^{\left(RD\right)}}=\mathrm{diag}\left(h_{1,\cdot }^{\left(RD\right)}\right){\left[\mathrm{diag}({\hat{h}}_{1,\cdot }^{\left(RD\right)})\right]}^{-1},\\ \; & \hat{S}\leftarrow {\hat{S}}_{\infty }\Lambda_{S}\;;\;\;\;{\hat{H}}^{\left(RD\right)}\leftarrow {\hat{H}}_{\infty }^{\left(RD\right)}\Lambda_{H^{\left(RD\right)}}\;;\;\;\;{\hat{H}}^{\left(SR\right)}\leftarrow \Lambda_{H^{\left(RD\right)}}^{-1}{\hat{H}}_{\infty }^{\left(SR\right)}\Lambda_{S}^{-1}, \end{array}$$

(202) $$\begin{array}{cc} \mathrm{NTD}\text{-}4:\;\; & \lambda_{S}=s_{1,1}/{\hat{s}}_{1,1}\;\;\mathrm{and}\;\;\lambda_{H^{\left(RD\right)}}=h_{1,1}^{\left(RD\right)}/{\hat{h}}_{1,1}^{\left(RD\right)},\\ \; & \hat{S}\leftarrow \lambda_{S}\;{\hat{S}}_{\infty }\;;\;\;\;{\hat{H}}^{\left(RD\right)}\leftarrow \lambda_{H^{\left(RD\right)}}\;{\hat{H}}_{\infty }^{\left(RD\right)}\;;\;\;\;{\hat{H}}^{\left(SR\right)}\leftarrow \lambda_{S}^{-1}\lambda_{H^{\left(RD\right)}}^{-1}\;{\hat{H}}_{\infty }^{\left(SR\right)}, \end{array}$$

where $\left({\hat{S}}_{\infty },{\hat{H}}_{\infty }^{\left(RD\right)},{\hat{H}}_{\infty }^{\left(SR\right)}\right)$ are estimates at convergence. In practice, the a priori information on the RD channel, necessary to remove ambiguities, can be obtained by applying a supervised procedure, that is to say a pilot symbol or a pilot sequence sent via the relay to the destination in the cases of KronF and KRF receivers, respectively. Several examples of this procedure can be found in the literature in the context of relay systems [40,41,42,43,55,58,60,100].

In Section 4, necessary conditions have been established for identifiability with ALS and closed-form estimation algorithms. By exploiting the correspondences in Table 26, identifiability conditions are derived in terms of design parameters for the proposed semi-blind receivers. For the KRF and KronF receivers of the DKRSTF, STST-MSMKron, and SKRST-MSMKR systems, identifiability conditions are given in (184), (193) and (194).

Table 29 summarizes the identifiability conditions for each proposed semi-blind receiver, as well as the transmission rate for each relay system. An analysis of the results in this table allows us to draw some preliminary conclusions with a view of choosing the design parameters in order to obtain the best trade-off between different performance criteria:

- ALS-based receivers are less constraining than closed-form ones. The matrices to be inverted in the ALS steps induce greater flexibility in the choice of design parameters compared to closed-form receivers which impose more restrictive conditions on these parameters.
- Matrix-based coding schemes induce fewer restrictions than tensor codings, i.e., softer constraints for the choice of design parameters.
- Exploiting chip diversity in the encoding process leads to identifiability conditions that are less constraining without degrading transmission rate.

## 7. Simulation Results

In this section, we present Monte Carlo simulation results to illustrate the performance of the proposed MIMO relay systems and associated semi-blind receivers.

For each system, information symbols are randomly drawn from a unit energy 4-QAM constellation. Channel coefficients are simulated as independent identically distributed (i.i.d.) complex random variables, following a Gaussian distribution with zero-mean and unit variance. The coding matrices/tensors are generated in such a way that each matrix or matrix unfolding to be inverted in the closed-form receiver algorithms is an orthonormal truncated discrete Fourier transform (DFT) matrix in order to simplify the computation of pseudo-inverses, as discussed in Remark 11.

All receiving antennas are assumed to be subject to an additive white Gaussian noise (AWGN), so the noisy received signal tensors are simulated as follows:

(203) $$\begin{array}{cc} X_{n}^{\left(R\right)} & =X^{\left(R\right)}+\sqrt{N_{0}}\;N^{\left(R\right)} \end{array}$$

(204) $$\begin{array}{cc} X_{n}^{\left(D\right)} & =X^{\left(D\right)}+\sqrt{N_{0}}\;N^{\left(D\right)}, \end{array}$$

where the noise-free tensors $X^{\left(R\right)}$ and $X^{\left(D\right)}$ are defined in Section 5 and Section 6, $N^{\left(R\right)}$ and $N^{\left(D\right)}$ represent unit energy noise tensors at the relay and the destination, respectively, and $N_{0}$ is the noise spectral density. At each Monte Carlo run, $N_{0}$ is calculated according to the desired signal-to-noise ratio (SNR) value, varying between −10 and 20 dB, and determined as follows:

(205) $$\mathrm{SNR}=10\;\log_{10}\left(\frac{∥X^{\left(D\right)}{∥}_{F}^{2}}{N_{0}}\right).$$

The criteria used to evaluate the proposed receivers are the SER and NMSEs of the estimated SR and RD channels and of the received signals tensor $X^{\left(D\right)}$, reconstructed using the parameters estimated at each Monte Carlo run. The corresponding curves are plotted versus the SNR, and the design parameter values are indicated at the top of each figure.

The SER is computed after the projection of the estimated symbols onto the symbol alphabet. The NMSE of estimated channels and reconstructed signals is defined as follows:

(206) $$\begin{array}{cc} \mathrm{NMSE}(H) & =10\;\log_{10}\left(\frac{1}{MC}\sum_{mc=1}^{MC}\frac{∥H_{mc}-{\hat{H}}_{mc}{∥}_{F}^{2}}{∥H_{mc}{∥}_{F}^{2}}\right)\\ \mathrm{NMSE}(X^{\left(D\right)}) & =10\;\log_{10}\left(\frac{1}{MC}\sum_{mc=1}^{MC}\frac{∥X_{mc}^{\left(D\right)}-{\hat{X}}_{mc}^{\left(D\right)}{∥}_{F}^{2}}{∥X_{mc}^{\left(D\right)}{∥}_{F}^{2}}\right), \end{array}$$

where MC denotes the number of Monte Carlo runs. $H_{mc}$ and ${\hat{H}}_{mc}$ represent the channel H simulated and estimated at the mc-th Monte Carlo run, respectively. $X_{mc}^{\left(D\right)}$ and ${\hat{X}}_{mc}^{\left(D\right)}$ are defined similarly. The performance criteria are averaged over $10^{4}$ Monte Carlo runs for various system configurations. In addition to the SER and NMSE curves, an assessment of the computational complexity of each receiver is also measured based on the computation time.

**Figure 19:**
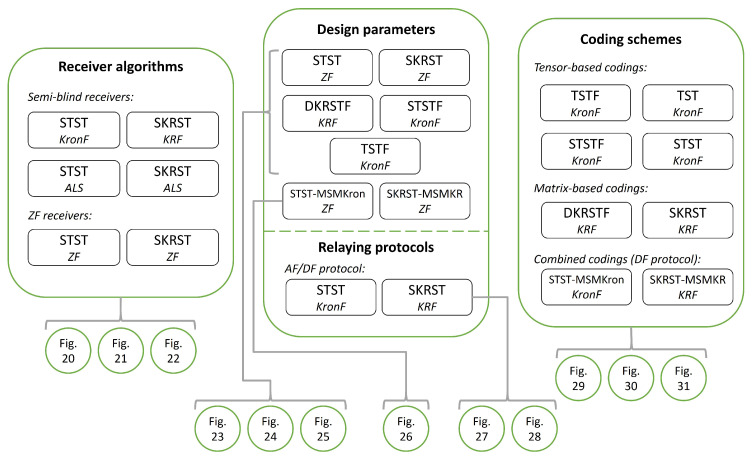
Plan of simulations for performance comparison.

To perform a comprehensive evaluation of the considered systems, we follow the methodology summarized in Figure 19, consisting of four main comparisons concerning receiver algorithms, design parameters, relaying protocols, and coding schemes. The STST and SKRST codings were selected as reference systems, except for in the last set of simulations, where all coding schemes are compared.

### 7.1. Comparison of Receiver Algorithms

The first simulations aimed to compare the performance of the closed-form (KronF/KRF) and ALS algorithms used in the semi-blind receivers presented in Table 28 for the STST and SKRST systems. A comparison was also conducted with the corresponding ZF receivers.

In the case of ALS receivers, the convergence is decided when the difference between two successive normalized reconstruction errors becomes smaller than a predefined threshold $\eta =10^{-5}$, i.e., $|\epsilon_{t}-\epsilon_{t-1}|\leq \eta$ with:

(207) $$\epsilon_{t}=\frac{∥X_{n}^{\left(D\right)}-{\hat{X}}_{t}^{\left(D\right)}{∥}_{F}^{2}}{∥X_{n}^{\left(D\right)}{∥}_{F}^{2}}.$$

**Figure 20:**
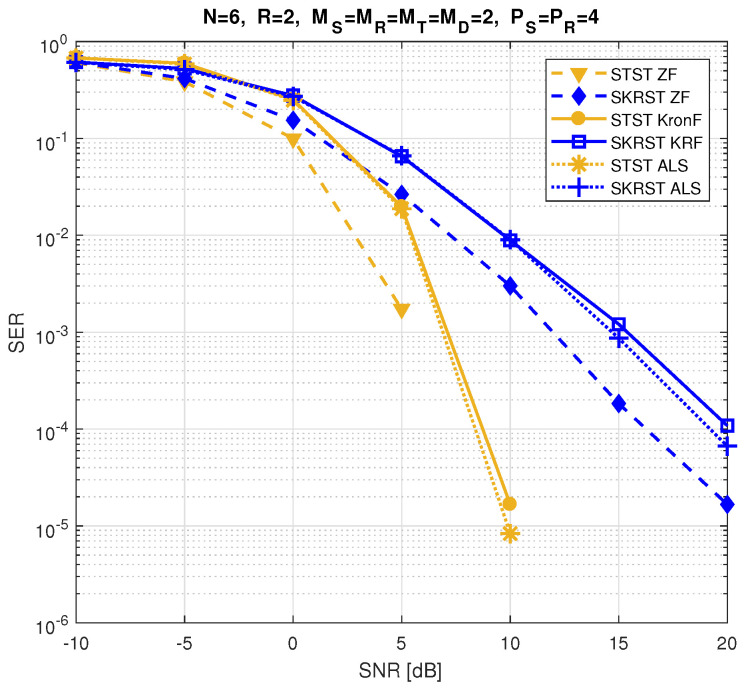
SER comparison with different receivers for STST and SKRST.

Figure 20 shows the SER versus SNR for the STST and SKRST systems. As expected, the best SER is obtained with ZF receivers due to a perfect knowledge of the channels at the destination. Slightly better performance is observed when ALS receivers are employed, compared to closed-form receivers. However, this improvement strongly depends on the choice of the threshold for convergence. A lower threshold improves the performance but at the cost of a higher computation time.

**Figure 21:**
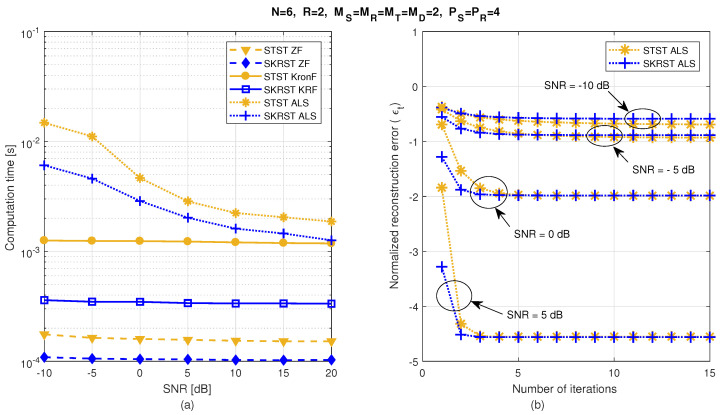
Comparison of (**a**) computation time for ZF, KronF/KRF, and ALS receivers and (**b**) number of iterations for convergence of ALS receivers for STST and SKRST.

From the results in Figure 20, we conclude that the STST system outperforms the SKRST system in terms of SER. For example, for a SER of $10^{-3}$, there is a gap of about 8 dB between the STST and SKRST performances. This is due to the use of tensor codings that provide increased diversity compared to matrix codings. Analyzing the signals encoded at the source, in Table 25, we conclude that, for STST, each symbol $s_{n,r}$ is repeated $M_{S}P_{S}$ times, while, for SKRST, each symbol $s_{n,m_{S}}$ is repeated only $P_{S}$ times. On the other hand, it is worth noting that the STST system requires a higher computation time with quasi the same number of iterations for convergence compared to the SKRST system, as illustrated in Figure 21a,b.

Figure 21a compares the computation times (in seconds) versus SNR, and Figure 21b shows the normalized reconstruction error $\epsilon_{t}$ versus the number of iterations for four different SNR values. As expected, closed-form and ZF receivers have constant computation times versus SNR, the shortest being obtained with ZF receivers since they only involve the estimation of symbols. Due to the knowledge of coding matrices and tensors, the three-step ALS receivers converge in very few iterations (three iterations for high SNR and up to six iterations for low SNR). Nevertheless, closed-form receivers require much less calculation than ALS-based ones, the cheapest being the KRF receiver for SKRST.

**Figure 22:**
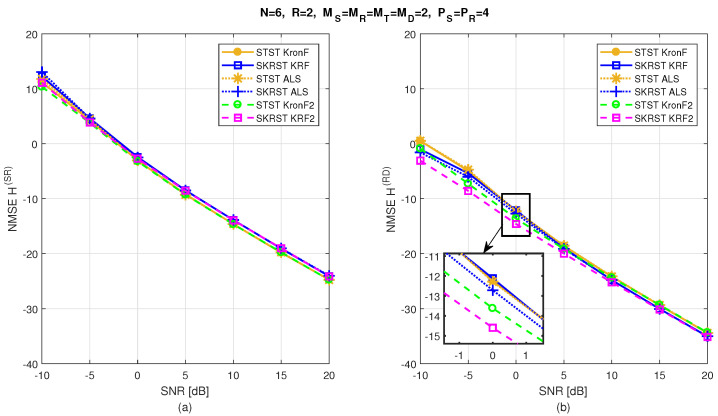
NMSE of estimated channels with the KronF/KRF and ALS receivers for STST and SKRST: (**a**) ${\hat{H}}^{\left(SR\right)}$ and (**b**) ${\hat{H}}^{\left(RD\right)}$.

Figure 22 shows the NMSE of the estimated channels $H^{\left(SR\right)}$ and $H^{\left(RD\right)}$ versus SNR for the ALS and KronF /KRF receivers of the STST and SKRST systems. For both channels, the performance of ALS receivers is very close to that of closed-form receivers. We note that the alternative method proposed in Section 6.2, denoted as KRF2 and KronF2 in Figure 22, which combines both closed-form algorithms in parallel, allows for a significant improvement of the estimate of $H^{\left(RD\right)}$.

From this first set of simulation results, we can conclude that ALS and closed-form receivers lead to a gap of approximately 3 dB for a SER of $10^{-2}$, compared to ZF receivers. Additionally, ALS receivers slightly outperform closed-form receivers, penalized by error propagation, at the cost of a higher computation time.

### 7.2. Impact of Design Parameters and Relay Protocol

The following simulations aimed to evaluate the impact of certain design parameters on the SER performance of STST and SKRST systems using ZF receivers. More precisely, we evaluate the impact of time-spreading lengths and numbers of antennas while keeping the other design parameters fixed. The impact of the number *Q* of symbol matrices in combined codings STST-MSMKron and SKRST-MSMKR is also evaluated.

#### 7.2.1. Impact of Time-Spreading Lengths: $P_{S}$ and $P_{R}$

**Figure 23:**
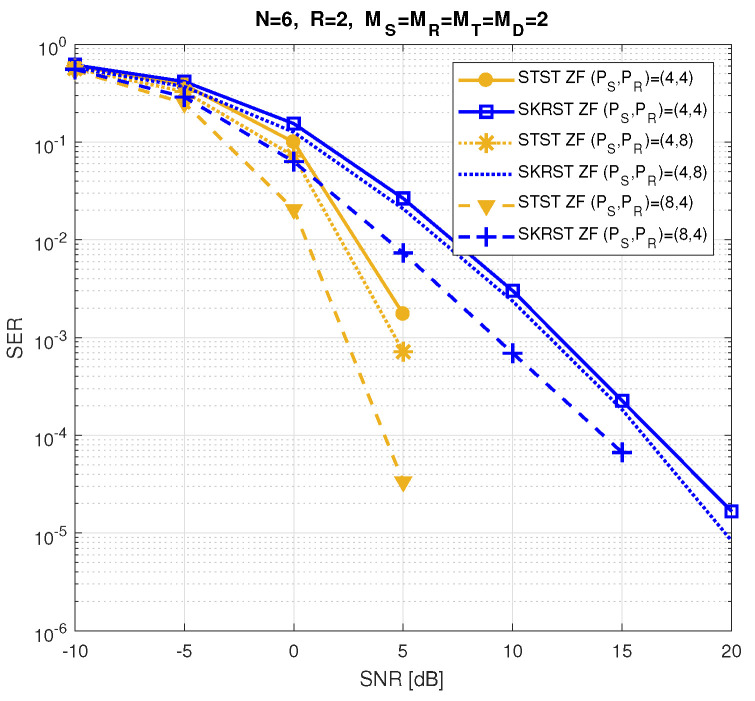
Impact of time-spreading lengths with ZF receivers of STST and SKRST.

Figure 23 shows the SER versus SNR for different values of time-spreading lengths at the source and relay nodes. We compare the following configurations: $\left(P_{S},P_{R}\right)=\left(4,4\right),\left(4,8\right)$, and (8,4). From the simulation results, we deduce that an increase in time-spreading lengths, whether at the source or the relay, leads to a gain in diversity and, therefore, to an improvement in the SER. A comparison of the configurations $\left(P_{S},P_{R}\right)=\left(4,8\right)$ and (8,4) shows that the SER improves more by increasing $P_{S}$, rather than $P_{R}$. This is because $P_{S}$ modifies the system diversity at both the relay and destination, while $P_{R}$ acts only at the destination. On the other hand, from Table 29, we conclude that an increase in $P_{S}$ degrades the transmission rate more than an increase in $P_{R}$.

The above conclusions regarding the impact of time-spreading lengths on SER performance are valid for both the STST and SKRST systems, with, however, a greater impact for STST, whose SER vanishes for an SNR greater than 5 dB.

#### 7.2.2. Impact of Numbers of Antennas: $M_{S}$, $M_{R}$, $M_{T}$, and $M_{D}$

**Figure 24:**
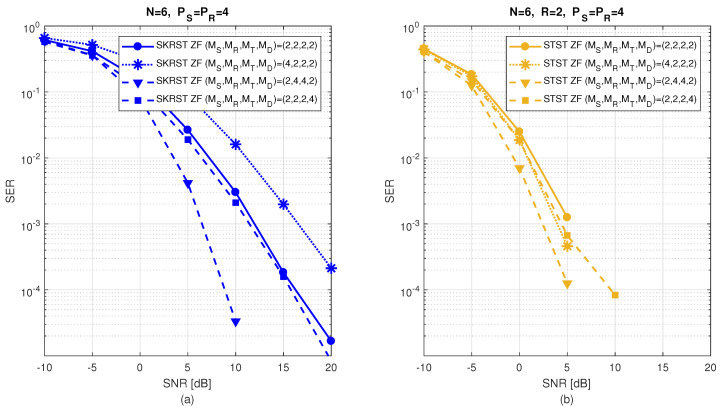
Impact of numbers of antennas with ZF receivers of (**a**) SKRST and (**b**) STST.

Figure 24 shows the SER versus SNR for different configurations of the number of antennas at the source ($M_{S}$), the relay ($M_{R},M_{T}$), and the destination ($M_{D}$). Due to the constraint imposed on the SKRST system, we assume that $M_{R}=M_{T}$ for the following configurations: $\left(M_{S},M_{R},M_{T},M_{D}\right)=\left(2,2,2,2\right)$, (4,2,2,2), (2,4,4,2), and (2,2,2,4), compared using the ZF receivers of STST and SKRST systems.

The (2,2,2,2) configuration serves as a reference to evaluate the impact of an increase in the number of antennas at the different nodes.

With the SKRST system (Figure 24a), increasing the number $M_{S}$ of antennas at the source leads to a degradation of the SER for all SNR values. This is due to the dimension of the symbol matrix depending on $M_{S}$ and, therefore, to an increase in the number of symbols to be estimated when $M_{S}$ is increased without changing the diversity of the system. With the (2,4,4,2) and (2,2,2,4) configurations, we observe some improvement in performance compared to the (2,2,2,2) configuration. In particular, an increase in the number $M_{R}$ of receive antennas at the relay allows a more significant improvement in the SER than an increase in the number $M_{D}$ of receiving antennas at the destination because $M_{R}$ is directly linked to the spatial diversity provided via the coding matrix $C^{\left(R\right)}$ at the relay, which is not the case for $M_{D}$.

For the STST system (Figure 24b), any increase in the numbers of antennas improves the SER. The (2,4,4,2) configuration provides the most significant improvement because $M_{R}$ and $M_{T}$ are both involved in the dimension of the coding tensor $C^{\left(R\right)}$ at the relay. Comparing the (4,2,2,2) and (2,2,2,4) configurations, we observe that both give nearly the same SER, with a slight advantage when increasing the number $M_{S}$ of antennas at the source.

**Figure 25:**
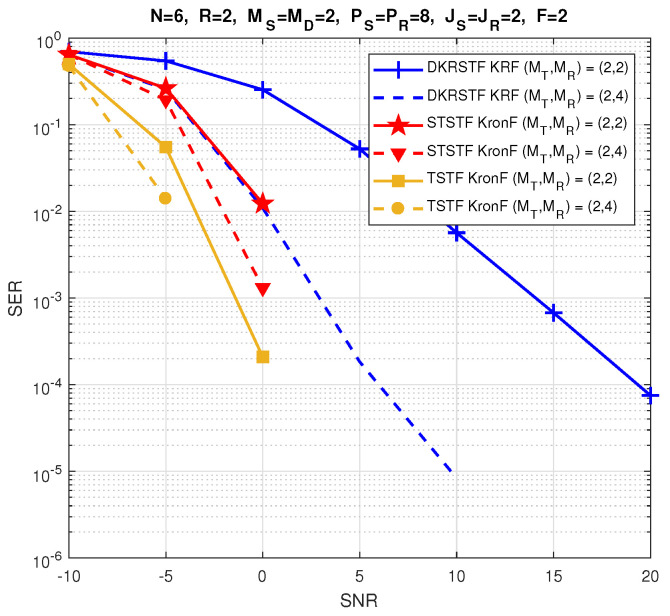
SER comparison for the DKRSTF, STSTF, and TSTF systems with $M_{R}\geq M_{T}$.

In Figure 25, we compare the DKRSTF, STSTF, and TSTF systems for the following configurations: $\left(M_{T},M_{R}\right)=\left(2,2\right)$ and (2,4). As discussed in Section 5.4.1, the configuration $M_{R}>M_{T}$ is associated with a virtual array at the destination for the DKRSTF system due to space coding at the relay. Note that all three systems exploit frequency diversity, with frequency-selective channels for the STSTF and TSTF systems, while flat-fading channels are assumed for the DKRSTF system. As expected, from the results on Figure 25, we conclude that increasing the number $M_{R}$ of receive antennas at the relay allows for an improvement of the SER, with the best improvement obtained using DKRSTF. For example, for a SER of $10^{-2}$, the DKRSTF system achieves a performance gain of approximately 8 dB between the two configurations, while the STSTF and TSTF systems provide a maximum gain of 2 dB. Note also that the $\left(M_{T},M_{R}\right)=\left(2,4\right)$ configuration with DKRSTF gives the same SER as the $\left(M_{T},M_{R}\right)=\left(2,2\right)$ configuration with STSTF because the doubling of $M_{R}$ compensates for the lack of frequency coding at the relay in the case of DKRSTF, while F=2 with STSTF. See the equivalence relationships (142) between the two codings at the relay. The TSTF system provides the best SER, thanks to a greater diversity provided via tensor coding.

#### 7.2.3. Impact of the Number *Q* of Symbol Matrices in Combined Codings

We now evaluate the impact of the number *Q* of symbol matrices in the MSMKron and MSMKR codings by varying the numbers $N_{q}$ of data streams for q∈〈Q〉, so as to have the same number of symbols to estimate for each value of *Q*.

**Figure 26:**
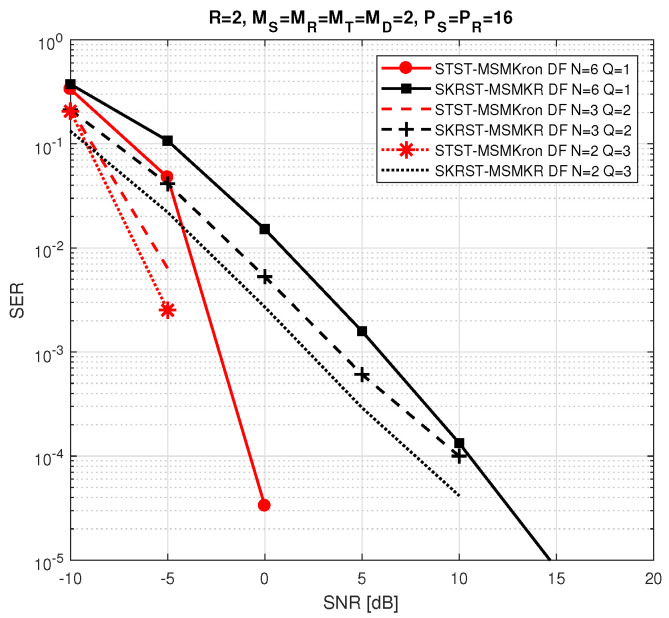
Impact of the number *Q* of symbol matrices in combined codings with ZF receivers.

Figure 26 shows the SER versus SNR obtained with the STST-MSMKron and SKRST-MSMKR systems for the following configurations: $\left(N_{q},Q\right)\in \left\{\left(6,1\right),\left(3,2\right),\left(2,3\right)\right\},q\in \langle Q\rangle$. From the simulation results, we conclude that increasing *Q* improves the SER, with the best performance obtained for Q=3, leading to an SNR gain of 3 dB with STST-MSMKron and 4 dB with SKRST-MSMKR, for a SER of $10^{-2}$, compared to the case of Q=1. This improvement is due to the fact that each symbol is repeated $N_{q}^{Q-1}$ times with the SKRST-MSMKR coding and ${\left(N_{q}R_{q}\right)}^{Q-1}$ times with the STST-MSMKron coding, which induces a greater repetition of each transmitted symbol when *Q* is increased, thanks to the mutual space-time spreading of information symbols provided via the multiple KRPs and KronPs of symbol matrices. The best SER is obtained with the STST-MSMKron coding.

#### 7.2.4. Impact of Relaying Protocol: AF and DF

In this section, we compare the impact of the AF and DF protocols for the STST and SKRST systems. With the DF protocol, the KronF and KRF receivers are directly deduced from the ones proposed for the combined codings by fixing Q=1. See Section 6.5 and Section 6.6.

**Figure 27:**
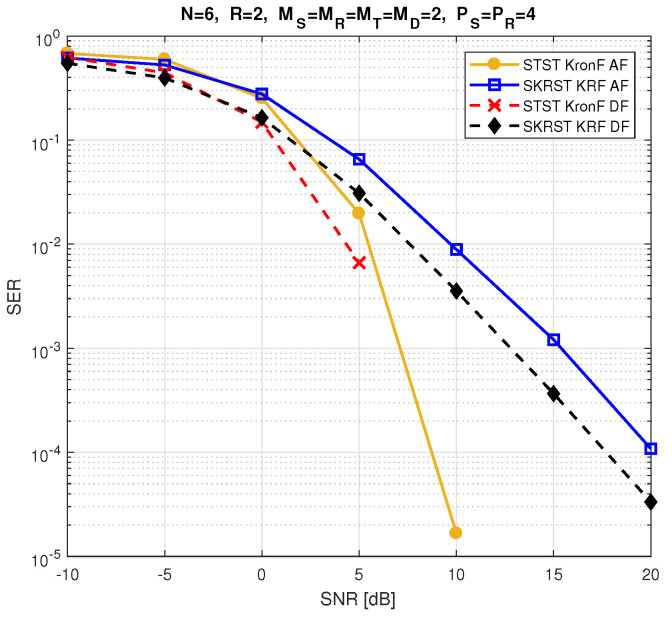
Impact of AF/DF protocols on SER performance of STST and SKRST.

**Figure 28:**
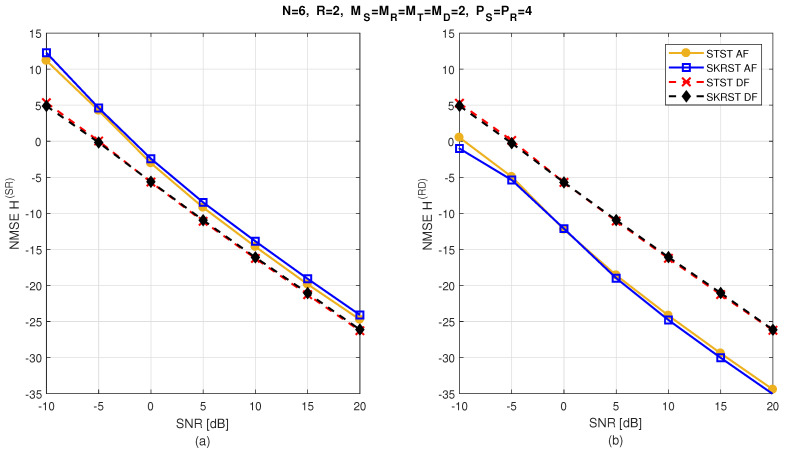
Impact of AF/DF protocols on NMSE of estimated channels for STST and SKRST: (**a**) ${\hat{H}}^{\left(SR\right)}$ and (**b**) ${\hat{H}}^{\left(RD\right)}$.

Figure 27 and Figure 28 respectively depict the SER and NMSE of estimated channels versus SNR. From Figure 27, we observe a clear advantage of using the DF protocol at the relay to improve the SER at the destination. Indeed, estimating the symbols at the relay before their re-encoding and then their transmission to the destination makes it possible to avoid the amplification of the noise contained in the signals received at the relay and directly coded in the case of the AF protocol. Moreover, it is obvious that, whatever protocol is used, the STST system achieves a better SER than the SKRST system. This corroborates the greater efficiency of tensor coding compared to matrix coding.

From Figure 28a,b, we observe that, for both protocols, the NMSEs of estimated channels obtained with the STST and SKRST systems are nearly identical, although the SKRST system uses more a priori information on the RD channel (a row instead of a single element of $H^{\left(RD\right)}$ with STST). This additional information compensates for the consideration of less diversity with SKRST than with STST.

Moreover, regarding the DF protocol, we observe that the SR and RD channels, which play a symmetrical role in terms of estimation, present the same NMSE. Note that this estimation is then perfectly blind in the sense that the a priori information needed to remove ambiguities only concerns the transmitted symbols.

As expected, and for the same reason as for the SER, from Figure 28a, we conclude that the SR channel is better estimated with the DF protocol than with AF, while, from Figure 28b, we draw the conclusion that the NMSE of the RD channel is smaller with AF than with DF, thanks to a priori information on the RD channel used with AF.

### 7.3. Comparison of Coding Schemes

**Figure 29:**
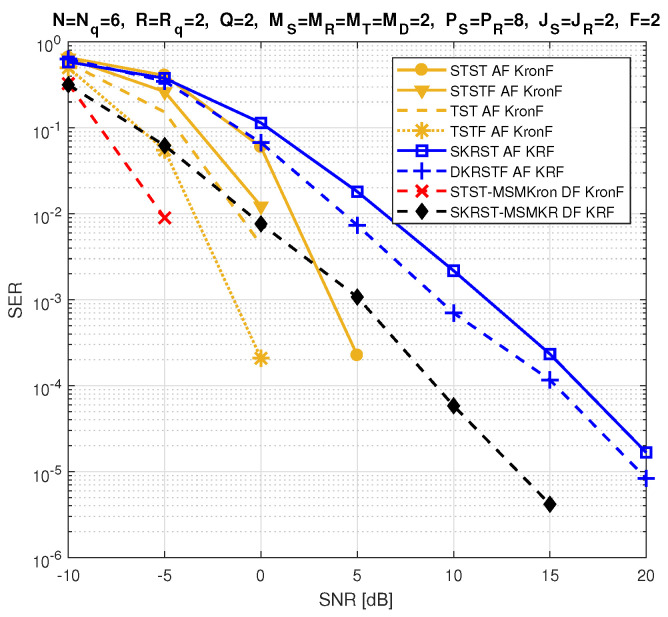
SER comparison for all considered relay systems.

**Figure 30:**
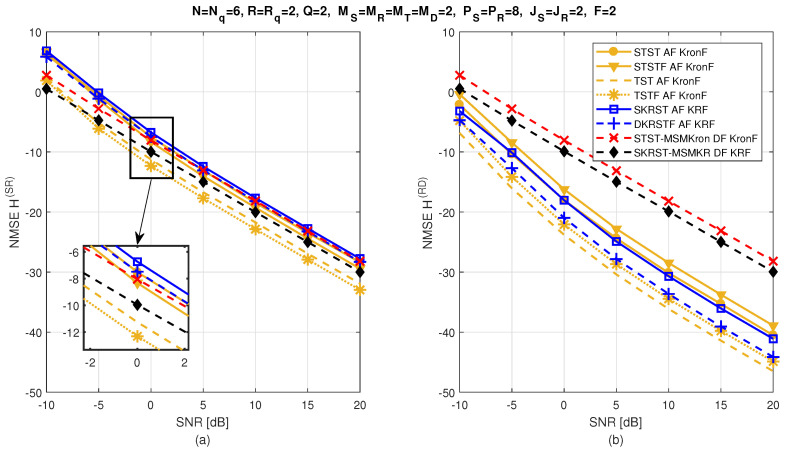
NMSE of estimated channels for all considered relay systems: (**a**) ${\hat{H}}^{\left(SR\right)}$ and (**b**) ${\hat{H}}^{\left(RD\right)}$.

**Figure 31:**
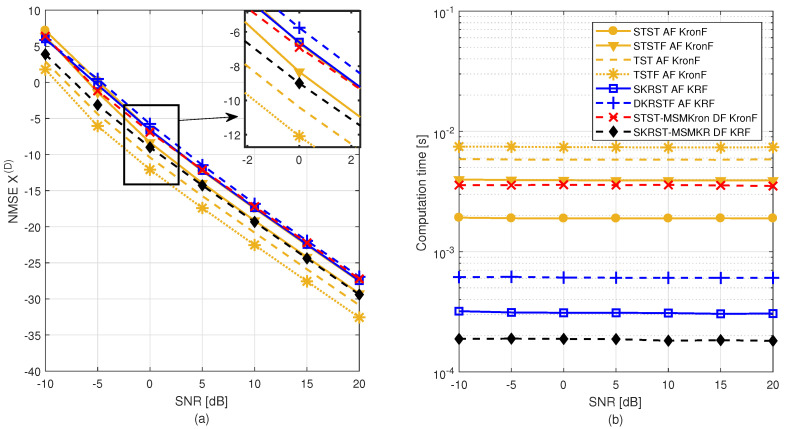
Comparison of considered relay systems in terms of (**a**) NMSE of reconstructed received signals and (**b**) computation time.

In this section, we carry out a comparison of all the relay systems considered in this paper, in terms of SER (Figure 29), the NMSE of estimated channels (Figure 30), the NMSE of reconstructed signals, and the computation time (Figure 31), versus SNR. The design parameters are chosen such that the transmission rate is approximately the same for all simulated systems.

In Figure 29, we observe the best performance when tensor-based codings (STST-MSMKron, TSTF, TST, STSTF, and STST) are used. The best SER is obtained with the combined STST-MSMKron coding, thanks to the multiple Kronecker product of symbol matrices that induces mutual diversity in the transmitted signals and to the use of the DF protocol.

For codings with the AF protocol, a classification can be made according to a continuous improvement in SER performance: (1) STST, (2) STSTF, (3) TST, and (4) TSTF, which corresponds to an increasing diversity associated with an increasing order (from four to seven) of the tensor $X^{\left(D\right)}$ of signals received at the destination.

Comparing the SERs obtained with TST and STSTF codings allows us to conclude that exploiting chip diversity at both the source and relay nodes ($J_{S}$ and $J_{R}$ dimensions of $X^{\left(D\right)}$) is more beneficial than exploiting frequency diversity, which introduces only one dimension (*F*) to $X^{\left(D\right)}$.

Regarding matrix-based codings, we draw a similar conclusion for the improvement in SER when increasing signal diversity, leading to the following classification (from worst to best): (1) SKRST, (2) DKRSTF, and (3) SKRST-MSMKR.

Concerning channel estimation, Figure 30a and Figure 30b respectively plot the NMSE of the estimated SR and RD channel matrices/tensors for all the systems considered.

To facilitate the comparative analysis of simulation results in these figures, Table 30 provides the numerical values of the NMSE of estimated channels for an SNR of 0 dB. This table also presents the values of the NMSE of reconstructed signals, shown in Figure 31a.

Note that the SR channel is estimated without a priori knowledge, unlike the RD channel for which a coefficient or a row is assumed to be known a priori to remove ambiguities, which explains a better estimation of the RD channel, except with the STST-MSMKron and SKRST-MSMKR systems for which the use of the DF protocol avoids any a priori knowledge on the channels.

As expected, the best NMSE results are achieved with codings that provide greater diversity. For instance, when comparing the TSTF and TST systems with their simplified versions, STSTF and STST, it is evident that TSTF and TST deliver superior performance.

For the same reason, tensor-based coding systems outperform matrix-based coding systems. It is also important to highlight the significant improvement in RD channel estimation observed with KRF-based receivers (for SKRST and DKRSTF systems) compared to KronF-based receivers (for STST and STSTF systems). This improvement is due to the a priori knowledge of an entire RD channel row required to remove ambiguities with KRF-based receivers, whereas KronF-based receivers only require a priori knowledge of a single channel coefficient, as detailed in Equations (200) and (197), respectively.

Finally, Figure 31 compares the performance of all relay systems in terms of the NMSE of reconstructed received signals and computation time. It is worth noting that the reconstruction of the signals received at the destination using symbol and channel estimates provides a meaningful measure to compare the effectiveness of the proposed semi-blind receivers.

In Figure 31a, we observe that the best performance is obtained with the TSTF system, with a SNR gain of about 2 dB compared to the TST system which gives the second best performance, for any SNR value. This better performance is achieved at the cost of a higher computation time, as shown in Figure 31b. In general, as already mentioned, we observe that the lowest performance is obtained with matrix codings that offer less diversity than tensor codings, inducing a lower computational complexity and, therefore, a lower computation time.

Comparing the results in Figure 31b and Table A1 in Appendix D makes the difference in computational cost even more evident. For instance, when comparing STST and SKRST systems which both generate fourth-order tensors for received signals, we observe that the complexity of the receiver STST/KronF is $O\left(NM^{2}PR\right)+O\left(M^{4}\right)$, while for SKRST/KRF, it is $O\left(NM^{2}P\right)+O\left(M^{3}\right)$, which corroborates the higher computational cost of STST/KronF, as shown in Figure 31b. A similar conclusion can be drawn for the comparison of STSTF/KronF and DKRSTF/KRF receivers. For the STST-MSMKRon/KronF receiver, the complexity increases with a multiplicative factor $R^{Q}$ which induces an exponential growth of the complexity as the number *Q* of symbol matrices increases in the coding scheme. This highlights the trade-off between computational cost and SER performance that improves as *Q* increases.

It should be noted that the computational costs depend more on the number of antennas than on other design parameters, with, in particular, $M^{4}$ and $M^{5}$ terms for the complexities of the KronF and ALS algorithms, respectively. This can become problematic in the case of massive MIMO systems involving a large number of antennas. For such systems, optimizing the computational cost will be crucial to avoid possible real-time difficulties.

Comparing the results in Figure 31a and Table 30 with those in Figure 29, we observe that the NMSE of reconstructed signals follows the same improvement as the SER when the system diversity is increased with the AF protocol, which is not the case of combined codings due to a degradation in channel estimation with the DF protocol. Regarding the robustness to noise, Table 31 provides the SNR thresholds from which the systems achieve the desired performance in terms of SER or NMSE of the reconstructed signals, deduced, respectively, from Figure 29 and Figure 31a. Two different values of SER and NMSE are considered. For example, for a fixed SER of $10^{-2}$, the STST-MSMKron system is the most robust to noise, achieving the desired SER performance from an SNR level of −5 dB. On the other hand, for the NMSE of the reconstructed signals set to −10 dB, the TSTF system is the most robust, with the required NMSE performance obtained from an SNR level of −2 dB. Table 31 highlights the systems with the best (green) and least (red) robustness to noise relative to the SNR levels required to achieve the desired performance. This analysis provides key information for selecting the system based on the desired balance between performance and noise tolerance. Note that the absence of an SNR threshold in Table 31 means that the corresponding performance is achieved for any SNR value greater than the largest value for which the SER is non-zero. For example, with the TST/KronF receiver, for any SNR value greater than 0 dB, the SER is zero.

To complement the previous comparison and help the user achieve the best trade-off between performance and complexity, taking into account the constraints imposed by the necessary identifiability conditions (NIC) and the required a priori knowledge (AK), Table 32 summarizes the characteristics of each system and the performance of associated semi-blind receivers, with the following criteria: (i) diversities, (ii) assumption of the channels (flat fading (FF) versus frequency selective fading (FSF)), and (iii) performance in terms of the NIC, AK, SER, NMSE of estimated channels, and computation time (CT). Performance is compared using positive signs (+) for advantages and negative signs (−) for disadvantages.

From Table 32, we can draw the following conclusions:

- *Diversity:* All coding schemes used at both the source and the relay include space-time (ST) coding. TST coding incorporates additional chip diversity, while STSTF and DKRSTF take into account frequency diversity. TSTF is the most comprehensive coding simultaneously exploiting the space-time–chip–frequency diversities (M,P,J,F), resulting in the best performance.
- *Channels:* Systems based on TSTF and STSTF codings consider frequency-selective fading channels, i.e., third-order channel tensors, while all other systems assume flat fading channels, i.e., channel matrices. It is worth noting that, despite a larger number of channel coefficients to be estimated, the best SER is obtained with TSTF coding due to the exploitation of all diversities, at the cost of a higher computation time.
- *Necessary identifiability conditions:* These conditions are related to the uniqueness of the pseudo-inverses of coding matrices or matrix unfoldings of coding tensors, depending on both the coding and the receiver type (ALS/KronF/KRF). ALS receivers are the least restrictive, i.e., the most flexible in the choice of design parameters. TSTF and TST are less restrictive than their simplified versions (STSTF and STST), thanks to the incorporation of chip diversity. Matrix codings entail fewer constraints than tensor codings. Note that frequency diversity has no impact on the identifiability conditions.
- *A priori knowledge:* With matrix codings (SKRST and DKRSTF) leading to CP models for received signals, associated semi-blind receivers use the KRF algorithm, which requires knowledge of an entire row of symbol and RD-channel matrices to remove ambiguities. On the other hand, systems based on tensor codings are modeled using Tucker or generalized Tucker models for which the semi-blind receivers use the KronF algorithm, which requires knowledge of only one element of the symbol and RD-channel matrices. With the DF protocol, only a few pilot symbols are needed, with receivers performing blind channel estimation in the sense that no a priori channel information is needed. In particular, the STST-MSMKron system requires knowledge of only one pilot symbol in each symbol matrix.
- *SER and NMSE of estimated channels:* These performances have already been commented in detail previously. It is worth highlighting that the TSTF and STST-MSMKron systems provide the best performance in terms of symbol estimation. Regarding channel estimation, the performance of the TSTF and TST systems are particularly remarkable.
- *Computation time:* As expected, iterative receivers based on the ALS algorithm require the highest computation times due to their iterative nature, which leads to a refinement in the estimation of unknown parameters. In general, the computation time reflects the amount of diversity taken into account. Higher diversity leads to higher-order tensors, which induce higher computation times. Matrix coding systems perform the best in terms of computational complexity.

In summary, the numerical simulations performed reveal a clear hierarchy in the performances of relay systems, depending on diversity management (via the choice of the coding scheme), relaying protocol and channel characteristics, which induce a more or less complex receiver and guide the choice of coding in order to satisfy a desired trade-off for a given application. Systems incorporating frequency and/or chip diversity present superior overall performance with less restrictive identifiability conditions using chip diversity and less a priori knowledge with KronF type receivers. Matrix-based codings are more suitable for low complexity requirements, but for better performance, tensor codings such as TST, TSTF, or STST-MSMKron are more appropriate, inducing higher computational costs. Moreover, these three coding schemes offer the best balance between SER and NMSE, in addition to exhibiting greater robustness to noise for different SNR levels.

## 8. Conclusions and Perspectives

In this paper, we have first provided an in-depth overview of tensor models, with a detailed description of nested tensor decompositions which are very useful to represent cooperative communication systems. Initially proposed for fourth-order tensors, nested decompositions have been generalized to higher-order tensors using graph-based representations and a novel interpretation in terms of cascading tensor models. Some new nested models have been introduced to represent relay systems using tensor codings. Several parameter estimation methods have been described, with a particular focus on closed-form algorithms based on KRF and KronF methods when some factors of nested models are a priori known, as is the case with coding matrices/tensors in the context of relay systems.

In a second part, a thorough survey of cooperative communication systems, and more particularly of one-way two-hop MIMO relay systems, was carried out using various coding strategies, with the aim of showing how these strategies impact the tensor model of signals received at the destination node. Capitalizing on this tensor model, two classes of semi-blind receivers were proposed to jointly estimate the transmitted information symbols and the individual communication channels: iterative receivers based on ALS and closed-form ones using KRF and KronF methods, which consist of rank-one matrix or tensor approximations. Extensive Monte Carlo simulation results were presented to analyze and compare the impact of the coding strategy, of the choice of design parameters, of the relay protocol and of the type of receiver on the system performance in terms of SER, channel estimate NMSE, and computation time. Simulation results show that the best SER performance is achieved with the most general TSTF tensor coding at the price of higher computational complexity.

Several extensions of this work can be considered, such as multi-user and multi-relay scenarios, two-way relaying case, and power and resource allocation, as well as three-dimensional (3D) polarized channels that combine dual-polarized antenna arrays with double-directional channels and time-varying mmWave channels characterized by an inherent sparse and low-rank structure that allows for the employment of compressed sensing methods. In terms of cooperative communications, combinations of relay-, IRS-, and UAV-aided communication systems are particularly interesting to study for future 6G wireless networks. We believe the tensor codings and nested tensor models highlighted in this paper for two-hop relay systems can be very useful in designing such new combined assisted communication networks.

The development of ISAC systems opens the way for new research problems for which sensing and cooperative communication systems are combined in a general framework allowing for estimates of target physical parameters, e.g., angles of arrival/departure (AoA/AoD), time delays, and Doppler shift, at a sensing base station, and communication channels and information symbols at an user equipment. The nested tensor models studied in this paper are also very attractive for modeling such ISAC systems.

## Figures and Tables

**Table 1:** Notation.

Symbols	Definitions
K=R or C	Set of real or complex numbers
〈N〉≜{1,⋯,N}	Set of first *N* integers
${\underline{i}}_{N}≜\left\{i_{1},\cdots ,i_{N}\right\}$	Set of *N* indices
${\underline{I}}_{N}≜I_{1}\times \cdots \times I_{N}$	Size of an *N*th-order tensor
*a*, a, A, A	Scalar, column vector, matrix, tensor
$a_{{\underline{i}}_{N}}=a_{i_{1},i_{2},\cdots ,i_{N}}$ or ${\left[A\right]}_{i_{1},i_{2},\cdots ,i_{N}}$	$\left(i_{1},i_{2},\cdots ,i_{N}\right)$-th element of $A\in K^{{\underline{I}}_{N}}$
$A^{T}$, $A^{*}$, $A^{H}$	Transpose, complex conjugate, Hermitian transpose of A
$A^{†}$	Moore–Penrose pseudo-inverse of A
$A_{i,•}$ $\left(A_{•,j}\right)$	*i*-th (*j*-th) row (column) of $A\in K^{I\times J}$
$I_{I_{N}}$	Identity matrix of size $I_{N}\times I_{N}$
$e_{n}^{\left(N\right)}$	*n*th canonical basis vector of the Euclidean space $R^{N}$
vec(·)	Vectorization operator
diag(·)	Diagonalization operator that forms a diagonal matrix from its vector argument
$D_{i}\left(A\right)=\mathrm{diag}\left(A_{i,•}\right)$	Diagonal matrix whose diagonal entries are the elements of the *i*-th row of $A\in K^{I\times J}$
${\mathrm{bdiag}}_{i}\left(A_{i,•,•}\right)$	Block-diagonal matrix whose diagonal blocks are horizontal slices * of $A\in K^{I\times J\times K}$
$\langle \cdot ,\cdot \rangle$	Inner product
${∥\cdot ∥}_{2}\;,\;{∥\cdot ∥}_{F}$	Euclidian and Frobenius norms
∘	Outer product
⊙	Hadamard product
⋄	Khatri–Rao product
⊗	Kronecker product
$\times_{n}$	Mode-*n* product
$\times_{p}^{n}$	Modes-(p,n) product

* See Table 4 for the definition of horizontal slices.

**Table 2:** Notation for sets of indices and dimensions.

${\underline{i}}_{P}≜\left\{i_{1},\cdots ,i_{P}\right\}$ ; ${\underline{j}}_{N}≜\left\{j_{1},\cdots ,j_{N}\right\}$
${\underline{I}}_{P}≜\left\{I_{1},\cdots ,I_{P}\right\}$ ; ${\underline{J}}_{N}≜\left\{J_{1},\cdots ,J_{N}\right\}$
${\underline{I}}_{P}≜I_{1}\times \cdots \times I_{P}$ ; ${\underline{J}}_{N}≜J_{1}\times \cdots \times J_{N}$

**Table 3:** Vector and matrix Kronecker products and matrix product using the index convention.

$u\in K^{I},v\in K^{J}$
$u⊗v=\left(u_{i}e_{i}^{\left(I\right)}\right)⊗\left(v_{j}e_{j}^{\left(J\right)}\right)=u_{i}v_{j}e_{ij}\in K^{IJ}$
$u⊗v^{T}=\left(u_{i}e_{i}^{\left(I\right)}\right)⊗\left(v_{j}{\left(e_{j}^{\left(J\right)}\right)}^{T}\right)=u_{i}v_{j}e_{i}^{j}\in K^{I\times J}$
$A\in K^{I\times J},B\in K^{J\times K},C\in K^{K\times M}$
$AB=\sum_{i=1}^{I}\sum_{k=1}^{K}\left(\sum_{j=1}^{J}a_{ij}b_{jk}\right)e_{i}^{k}=a_{ij}b_{jk}e_{i}^{k}\in K^{I\times K}$
$A⊗C=\left(a_{ij}e_{i}^{j}\right)⊗\left(b_{km}\right)e_{k}^{m})=a_{ij}b_{km}e_{ik}^{jm}\in K^{IK\times JM}$

**Table 4:** Vector and matrix slices for a third-order tensor, $X\in K^{I\times J\times K}$.

Slices	Definitions	Dimensions
Vectors	Fibers	
column	$x_{•,j,k}$	*I*
row	$x_{i,•,k}$	*J*
tube	$x_{i,j,•}$	*K*
Matrices	Matrix slices	
frontal	$X_{•,•,k}$	I×J
lateral	$X_{•,j,•}$	K×I
horizontal	$X_{i,•,•}$	J×K

**Table 5:** Definitions of some basic operations.

Vectors/Matrices/Tensors	Operations	Definitions
$u^{\left(p\right)}\in K^{I_{p}},p\in \langle P\rangle$	$X=\overset{P}{\underset{p=1}{\circ }}u^{\left(p\right)}$	$x_{{\underline{i}}_{P}}=\prod_{p=1}^{P}u_{i_{p}}^{\left(p\right)}$
$X\in K^{{\underline{I}}_{P}},A\in K^{J\times I_{p}}$	$Y=X\times_{p}A$	$y_{i_{1},\cdots ,i_{p-1}\;,j,\;i_{p+1},\cdots ,i_{P}}=\sum_{i_{p}=1}^{I_{p}}a_{j,i_{p}}x_{{\underline{i}}_{P}}=a_{j,i_{p}}x_{{\underline{i}}_{P}}$
$X\in K^{{\underline{I}}_{P}},A^{\left(p\right)}\in K^{J_{p}\times I_{p}},p\in \langle P\rangle$	$Y=X\overset{P}{\underset{p=1}{\times }}A^{\left(p\right)}≜\;X\times_{1}A^{\left(1\right)}\times_{2}\cdots \times_{P}A^{\left(P\right)}$	$y_{{\underline{j}}_{P}}=\sum_{i_{1}=1}^{I_{1}}\cdots \sum_{i_{P}=1}^{I_{P}}\;\prod_{p=1}^{P}a_{j_{p},i_{p}}^{\left(p\right)}x_{{\underline{i}}_{P}}=\prod_{p=1}^{P}a_{j_{p},i_{p}}^{\left(p\right)}x_{{\underline{i}}_{P}}$
$X\in K^{{\underline{I}}_{P}},Y\in K^{{\underline{J}}_{N}}$	$Z=X\times_{p}^{n}Y$	$z_{i_{1},\cdots ,i_{p-1},\;i_{p+1},\cdots ,i_{P},j_{1},\cdots ,j_{n-1},\;j_{n+1},\cdots ,j_{N}}=$
with $I_{p}=J_{n}=K$		$\sum_{k=1}^{K}\;a_{i_{1},\cdots ,i_{p-1},\;k,\;i_{p+1},\cdots ,i_{P}}\;b_{j_{1},\cdots ,j_{n-1},\;k,\;j_{n+1},\cdots ,j_{N}}$

**Table 6:** TD of an *N*th-order and third-order tensor.

	*N*th-Order Tensor	Third-Order Tensor
Tensors	$X\in K^{{\underline{I}}_{N}}$	$X\in K^{I\times J\times K}$
Core tensors	$G\in K^{{\underline{R}}_{N}}$	$G\in K^{P\times Q\times S}$
Matrix factors	$A^{\left(n\right)}\in K^{I_{n}\times R_{n}}$	$A\in K^{I\times P},B\in K^{J\times Q},C\in K^{K\times S}$
Scalar writing	$x_{{\underline{i}}_{N}}=\sum_{r_{1}=1}^{R_{1}}\cdots \sum_{r_{N}=1}^{R_{N}}g_{r_{1},\cdots ,r_{N}}\prod_{n=1}^{N}a_{i_{n},r_{n}}^{\left(n\right)}$	$x_{i,j,k}=\sum_{p=1}^{P}\sum_{q=1}^{Q}\sum_{s=1}^{S}g_{pqs}a_{ip}b_{jq}c_{ks}$
With mode-*n* products	$X=G\overset{N}{\underset{n=1}{\times }}A^{\left(n\right)}$	$X=G\times_{1}A\times_{2}B\times_{3}C$
With outer products	$X=\sum_{r_{1}=1}^{R_{1}}\cdots \sum_{r_{N}=1}^{R_{N}}g_{r_{1},\cdots ,r_{N}}\overset{N}{\underset{n=1}{\circ }}A_{.r_{n}}^{\left(n\right)}$	$X=\sum_{p=1}^{P}\sum_{q=1}^{Q}\sum_{s=1}^{S}g_{pqs}A_{.p}\circ B_{.q}\circ C_{.s}$
		$X_{IJ\times K}=\left(A⊗B\right)G_{PQ\times S}C^{T}$
Matrix unfoldings	$X_{S_{1};S_{2}}=\left(\underset{n\in S_{1}}{⊗}A^{\left(n\right)}\right)G_{S_{1};S_{2}}{\left(\underset{n\in S_{2}}{⊗}A^{\left(n\right)}\right)}^{T}$	$X_{JK\times I}=\left(B⊗C\right)G_{QS\times P}A^{T}$
		$X_{KI\times J}=\left(C⊗A\right)G_{SP\times Q}B^{T}$

**Table 7:** CPD of an *N*th-order and third-order tensor.

	*N*th-Order Tensor	Third-Order Tensor
Tensors	$X\in K^{{\underline{I}}_{N}}$	$X\in K^{I\times J\times K}$
Core tensors	$I_{N,R}\in R^{R\times R\times ...\times R}$	$I_{3,R}\in R^{R\times R\times R}$
Matrix factors	$A^{\left(n\right)}\in K^{I_{n}\times R}$	$A\in K^{I\times R},B\in K^{J\times R},C\in K^{K\times R}$
Scalar writing	$x_{{\underline{i}}_{N}}=\sum_{r}^{R}\prod_{n=1}^{N}a_{i_{n},r}^{\left(n\right)}$	$x_{i,j,k}=\sum_{r=1}^{R}a_{ir}b_{jr}c_{kr}$
With mode-*n* products	$X=I_{N,R}\overset{N}{\underset{n=1}{\times }}A^{\left(n\right)}$	$X=I_{3,R}\times_{1}A\times_{2}B\times_{3}C$
With outer products	$X=\sum_{r=1}^{R}\overset{N}{\underset{n=1}{\circ }}A_{.r}^{\left(n\right)}$	$X=\sum_{r=1}^{R}A_{.r}\circ B_{.r}\circ C_{.r}$
		$X_{IJ\times K}=\left(A⋄B\right)C^{T}$
Matrix unfoldings	$X_{S_{1};S_{2}}=\left(\underset{n\in S_{1}}{⋄}A^{\left(n\right)}\right){\left(\underset{n\in S_{2}}{⋄}A^{\left(n\right)}\right)}^{T}$	$X_{JK\times I}=\left(B⋄C\right)A^{T}$
		$X_{KI\times J}=\left(C⋄A\right)B^{T}$

**Table 8:** Tucker-$\left(N_{1},N\right)$ decomposition and special cases for N=3.

$X\in K^{{\underline{I}}_{N}}$
TD-$\left(N_{1},N\right)$ model, $N\geq N_{1}$
$A^{\left(n\right)}\in K^{I_{n}\times R_{n}}$, for $n\in \langle N_{1}\rangle$, $A^{\left(n\right)}=I_{I_{n}}$, for $n=N_{1}+1,\cdots ,N$, $G\in K^{R_{1}\times \cdots \times R_{N_{1}}\times I_{N_{1}+1}\times \cdots \times I_{N}}$
$x_{{\underline{i}}_{N}}=\sum_{r_{1}=1}^{R_{1}}\cdots \sum_{r_{N_{1}}=1}^{R_{N_{1}}}g_{r_{1},\cdots ,r_{N_{1}},i_{N_{1}+1},\cdots ,i_{N}}\prod_{n=1}^{N_{1}}a_{i_{n},r_{n}}^{\left(n\right)}$
$X=G\times_{1}A^{\left(1\right)}\times_{2}\cdots \times_{N_{1}}A^{\left(N_{1}\right)}\times_{N_{1}+1}I_{I_{N_{1}+1}}\cdots \times_{N}I_{I_{N}}=G\overset{N_{1}}{\underset{n=1}{\times }}A^{\left(n\right)}$
Special cases for N=3
$X\in K^{I\times J\times K}$
Tucker(2,3) model
$G\in K^{P\times Q\times K}$, $A\in K^{I\times P},B\in K^{J\times Q},C=I_{K}$
$x_{ijk}=\sum_{p=1}^{P}\sum_{q=1}^{Q}g_{pqk}a_{ip}b_{jq}$, $X=G\times_{1}A\times_{2}B$
Tucker(1,3) model
$A\in K^{I\times P},B=I_{J},C=I_{K}$
$x_{ijk}=\sum_{p=1}^{P}g_{pjk}a_{ip}$, $X=G\times_{1}A$

**Table 9:** Generalized Tucker models.

GTD-(2,4) model
$X\in K^{{\underline{I}}_{4}}$
$G\in K^{R_{1}\times R_{2}\times I_{3}\times I_{4}}\;,\;A\in K^{I_{1}\times R_{1}\times I_{3}}\;,\;B\in K^{I_{2}\times R_{2}}$
$x_{{\underline{i}}_{4}}=\sum_{r_{1}=1}^{R_{1}}\sum_{r_{2}=1}^{R_{2}}g_{r_{1},r_{2},i_{3},i_{4}}a_{i_{1},r_{1},i_{3}}b_{i_{2},r_{2}}\;\;,\;\;X=G\times_{1}^{2}A\times_{2}B$

**Table 10:** TTD for a *P*th-order tensor, $X\in K^{{\underline{I}}_{P}}$.

$X\in K^{{\underline{I}}_{P}}$
$G^{\left(1\right)}\in K^{I_{1}\times R_{1}}\;\;\;;\;\;\;G^{\left(P\right)}\in K^{R_{P-1}\times I_{P}}\;\;\;;\;\;\;G^{\left(p\right)}\in K^{R_{p-1}\times I_{p}\times R_{p}},p\in \left\{2,\cdots ,P-1\right\}$
$x_{{\underline{i}}_{P}}=\sum_{{\underline{r}}_{P-1}=\underline{1}}^{{\underline{R}}_{P-1}}\prod_{p=1}^{P}g_{r_{p-1},i_{p},r_{p}}^{\left(p\right)}\;\;\;\mathrm{with}\;\;\;\;g_{r_{0},i_{1},r_{1}}^{\left(1\right)}=g_{i_{1},r_{1}}^{\left(1\right)}$ and $g_{r_{P-1},i_{P},r_{P}}^{\left(P\right)}=g_{r_{P-1},i_{P}}^{\left(P\right)}$
$x_{{\underline{i}}_{P}}=g_{i_{1},•}^{\left(1\right)}\;G_{•,i_{2},•}^{\left(2\right)}G_{•,i_{3},•}^{\left(3\right)}\cdots G_{•,i_{P-1},•}^{\left(P-1\right)}\;g_{•,i_{P}}^{\left(P\right)}$
$X=\left(\left(\cdots \left(\left(G^{\left(1\right)}\times_{2}^{1}G^{\left(2\right)}\right)\times_{3}^{1}G^{\left(3\right)}\right)\times_{4}^{1}\cdots \right)\times_{P-1}^{1}G^{\left(P-1\right)}\right)\times_{P}^{1}G^{\left(P\right)}$
$X=\sum_{r_{1}=1}^{R_{1}}\cdots \sum_{r_{P-1}=1}^{R_{P-1}}g_{•,r_{1}}^{\left(1\right)}\circ g_{r_{1},•,r_{2}}^{\left(2\right)}\cdots \circ g_{r_{P-2},•,r_{P-1}}^{\left(P-1\right)}\circ {\left[g_{r_{P-1},•}^{\left(P\right)}\right]}^{T}$

**Table 11:** Parametric complexity of CPD, TD, and TTD.

Models	Notations	Element $x_{{\underline{i}}_{N}}$
CPD	$〚A^{\left(1\right)},\cdots ,A^{\left(N\right)};R\;〛$	$\sum_{r=1}^{R}\prod_{n=1}^{N}a_{i_{n},r}^{\left(n\right)}$
TD	$〚G\;;A^{\left(1\right)},\cdots ,A^{\left(N\right)};R_{1},\cdots ,R_{N}〛$	$\sum_{r_{1}=1}^{R_{1}}\cdots \sum_{r_{N}=1}^{R_{N}}g_{r_{1},\cdots ,r_{N}}\prod_{n=1}^{N}a_{i_{n},r_{n}}^{\left(n\right)}$
TTD	$〚G^{\left(1\right)},G^{\left(2\right)},\cdots ,G^{\left(N-1\right)},G^{\left(N\right)};R_{1},\cdots ,R_{N-1}〛$	$\sum_{r_{1}=1}^{R_{1}}\cdots \sum_{r_{N-1}=1}^{R_{N-1}}\prod_{n=1}^{N}g_{r_{n-1},i_{n},r_{n}}^{\left(n\right)}\;\;,\;\;r_{0}=r_{N}$
**Models**	**Parameters**	**Complexity**
CPD	$A^{\left(n\right)}\in K^{I_{n}\times R}\;,\;\forall n\in \langle N\rangle$	O(NIR)
TD	$G\in K^{{\underline{R}}_{N}};A^{\left(n\right)}\in K^{I_{n}\times R_{n}}\;,\;\forall n\in \langle N\rangle$	$O(NIR+R^{N})$
	$G^{\left(1\right)}\in K^{I_{1}\times R_{1}}\;,\;G^{\left(N\right)}\in K^{R_{N-1}\times I_{N}}$	
TTD	$G^{\left(n\right)}\in K^{R_{n-1}\times I_{n}\times R_{n}},\;\forall n\in \left\{2,3,\cdots ,N-1\right\}$	$O\left(2IR+\left(N-2\right)IR^{2}\right)$

**Table 12:** TTD, NCPD, and NTD models for a fourth-order tensor.

$X\in K^{{\underline{I}}_{4}}$
TTD-4 model
$G^{\left(1\right)}\in K^{I_{1}\times R_{1}}\;\;;\;\;G^{\left(4\right)}\in K^{R_{3}\times I_{4}}\;\;\;;\;\;\;G^{\left(p\right)}\in K^{R_{p-1}\times I_{p}\times R_{p}},p\in \left\{2,3\right\}$
$x_{{\underline{i}}_{4}}=\sum_{r_{1}=1}^{R_{1}}\sum_{r_{2}=1}^{R_{2}}\sum_{r_{3}=1}^{R_{3}}g_{i_{1},r_{1}}^{\left(1\right)}g_{r_{1},i_{2},r_{2}}^{\left(2\right)}g_{r_{2},i_{3},r_{3}}^{\left(3\right)}g_{r_{3},i_{4}}^{\left(4\right)}$
NCPD-4 model
$A^{\left(1\right)}\in K^{I_{1}\times R_{1}}\;\;\;;\;\;\;B^{\left(1\right)}\in K^{I_{2}\times R_{1}}\;\;;\;\;G\in K^{R_{1}\times R_{2}}\;\;;\;A^{\left(2\right)}\in K^{I_{3}\times R_{2}}\;\;\;;\;\;\;B^{\left(2\right)}\in K^{I_{4}\times R_{2}}$
$x_{{\underline{i}}_{4}}=\sum_{r_{1}=1}^{R_{1}}\sum_{r_{2}=1}^{R_{2}}a_{i_{1},r_{1}}^{\left(1\right)}b_{i_{2},r_{1}}^{\left(1\right)}g_{r_{1},r_{2}}a_{i_{3},r_{2}}^{\left(2\right)}b_{i_{4},r_{2}}^{\left(2\right)}$
NTD-4 model
$A^{\left(1\right)}\in K^{I_{1}\times R_{1}}\;\;\;;\;\;\;G^{\left(1\right)}\in K^{R_{1}\times I_{2}\times R_{2}}\;\;\;;\;\;\;U\in K^{R_{2}\times R_{3}}\;\;;\;\;G^{\left(2\right)}\in K^{R_{3}\times I_{3}\times R_{4}}\;\;;\;A^{\left(2\right)}\in K^{I_{4}\times R_{4}}$
$x_{{\underline{i}}_{4}}=\sum_{r_{1}=1}^{R_{1}}\sum_{r_{2}=1}^{R_{2}}\sum_{r_{3}=1}^{R_{3}}\sum_{r_{4}=1}^{R_{4}}a_{i_{1},r_{1}}^{\left(1\right)}g_{r_{1},i_{2},r_{2}}^{\left(1\right)}u_{r_{2},r_{3}}g_{r_{3},i_{3},r_{4}}^{\left(2\right)}a_{i_{4},r_{4}}^{\left(2\right)}$

**Table 13:** Matrix unfoldings of CPD models.

CPD model of $X^{\left(1\right)}=〚A^{\left(1\right)},B^{\left(1\right)},G^{T};R_{1}〛\in K^{I_{1}\times I_{2}\times R_{2}}$
$X_{I_{1}I_{2}\times R_{2}}^{\left(1\right)}=\left(A^{\left(1\right)}⋄B^{\left(1\right)}\right)G$
$X_{I_{1}R_{2}\times I_{2}}^{\left(1\right)}=\left(A^{\left(1\right)}⋄G^{T}\right){\left(B^{\left(1\right)}\right)}^{T}$
CPD model of $X^{\left(2\right)}=〚G,A^{\left(2\right)},B^{\left(2\right)};R_{2}〛\in K^{R_{1}\times I_{3}\times I_{4}}$
$X_{I_{3}I_{4}\times R_{1}}^{\left(2\right)}=\left(A^{\left(2\right)}⋄B^{\left(2\right)}\right)G^{T}$
$X_{I_{4}R_{1}\times I_{3}}^{\left(2\right)}=\left(B^{\left(2\right)}⋄G\right){\left(A^{\left(2\right)}\right)}^{T}$
CPD model of $X_{c_{1}}=〚A^{\left(1\right)},B^{\left(1\right)},X_{I_{3}I_{4}\times R_{1}}^{\left(2\right)};R_{1}〛\in K^{I_{1}\times I_{2}\times I_{3}I_{4}}$
$X_{I_{1}I_{3}I_{4}\times I_{2}}=(A^{\left(1\right)}⋄X_{I_{3}I_{4}\times R_{1}}^{\left(2\right)}){\left(B^{\left(1\right)}\right)}^{T}$
$X_{I_{2}I_{3}I_{4}\times I_{1}}=\left(B^{\left(1\right)}⋄X_{I_{3}I_{4}\times R_{1}}^{\left(2\right)}\right){\left(A^{\left(1\right)}\right)}^{T}$
$X_{I_{1}I_{2}\times I_{3}I_{4}}=\left(A^{\left(1\right)}⋄B^{\left(1\right)}\right)X_{R_{1}\times I_{3}I_{4}}^{\left(2\right)}$
CPD model of $X_{c_{2}}=〚X_{I_{1}I_{2}\times R_{2}}^{\left(1\right)},A^{\left(2\right)},B^{\left(2\right)};R_{2}〛\in K^{I_{1}I_{2}\times I_{3}\times I_{4}}$
$X_{I_{3}I_{1}I_{2}\times I_{4}}=\left(A^{\left(2\right)}⋄X_{I_{1}I_{2}\times R_{2}}^{\left(1\right)}\right){\left(B^{\left(2\right)}\right)}^{T}$
$X_{I_{4}I_{1}I_{2}\times I_{3}}=(B^{\left(2\right)}⋄X_{I_{1}I_{2}\times R_{2}}^{\left(1\right)}){\left(A^{\left(2\right)}\right)}^{T}$
$X_{I_{1}I_{2}\times I_{3}I_{4}}=X_{I_{1}I_{2}\times R2}^{\left(1\right)}{\left(A^{\left(2\right)}⋄B^{\left(2\right)}\right)}^{T}$

**Table 14:** Matrix unfoldings of the NCPD-4 model.

NCPD-4 model of $X=〚A^{\left(1\right)},B^{\left(1\right)},G,A^{\left(2\right)},B^{\left(2\right)};R_{1},R_{2}〛\in K^{{\underline{I}}_{4}}$
$X_{I_{1}I_{3}I_{4}\times I_{2}}=\left(A^{\left(1\right)}⋄X_{I_{3}I_{4}\times R_{1}}^{\left(2\right)}\right){\left(B^{\left(1\right)}\right)}^{T}=\left[A^{\left(1\right)}⋄\left(A^{\left(2\right)}⋄B^{\left(2\right)}\right)G^{T}\right]{\left(B^{\left(1\right)}\right)}^{T}$

$X_{I_{2}I_{3}I_{4}\times I_{1}}=\left(B^{\left(1\right)}⋄X_{I_{3}I_{4}\times R_{1}}^{\left(2\right)}\right){\left(A^{\left(1\right)}\right)}^{T}=\left[B^{\left(1\right)}⋄\left(A^{\left(2\right)}⋄B^{\left(2\right)}\right)G^{T}\right]{\left(A^{\left(1\right)}\right)}^{T}$

$X_{I_{3}I_{1}I_{2}\times I_{4}}=\left(A^{\left(2\right)}⋄X_{I_{1}I_{2}\times R_{2}}^{\left(1\right)}\right){\left(B^{\left(2\right)}\right)}^{T}=\left[A^{\left(2\right)}⋄\left(A^{\left(1\right)}⋄B^{\left(1\right)}\right)G\right]{\left(B^{\left(2\right)}\right)}^{T}$

$X_{I_{4}I_{1}I_{2}\times I_{3}}=\left(B^{\left(2\right)}⋄X_{I_{1}I_{2}\times R_{2}}^{\left(1\right)}\right){\left(A^{\left(2\right)}\right)}^{T}=\left[B^{\left(2\right)}⋄\left(A^{\left(1\right)}⋄B^{\left(1\right)}\right)G\right]{\left(A^{\left(2\right)}\right)}^{T}$

$X_{I_{1}I_{2}\times I_{3}I_{4}}=\left(A^{\left(1\right)}⋄B^{\left(1\right)}\right)X_{R_{1}\times I_{3}I_{4}}^{\left(2\right)}=X_{I_{1}I_{2}\times R2}^{\left(1\right)}{\left(A^{\left(2\right)}⋄B^{\left(2\right)}\right)}^{T}=\left(A^{\left(1\right)}⋄B^{\left(1\right)}\right)G{\left(A^{\left(2\right)}⋄B^{\left(2\right)}\right)}^{T}$

**Table 15:** Matrix unfoldings of TD models.

TD model of $X^{\left(1\right)}=〚G^{\left(1\right)};A^{\left(1\right)},I_{I_{2}},U^{T};R_{1},R_{2}〛\in K^{I_{1}\times I_{2}\times R_{3}}$
$X_{I_{1}I_{2}\times R_{3}}^{\left(1\right)}=\left(A^{\left(1\right)}⊗I_{I_{2}}\right)G_{R_{1}I_{2}\times R_{2}}^{\left(1\right)}U$
$X_{I_{1}R_{3}\times I_{2}}^{\left(1\right)}=\left(A^{\left(1\right)}⊗U^{T}\right)G_{R_{1}R_{2}\times I_{2}}^{\left(1\right)}$
TD model of $X^{\left(2\right)}=〚G^{\left(2\right)};U,I_{I_{3}},A^{\left(2\right)};R_{3},R_{4}〛\in K^{R_{2}\times I_{3}\times I_{4}}$
$X_{I_{3}I_{4}\times R_{2}}^{\left(2\right)}=\left(I_{I_{3}}⊗A^{\left(2\right)}\right)G_{I_{3}R_{4}\times R_{3}}^{\left(2\right)}U^{T}$
$X_{I_{4}R_{2}\times I_{3}}^{\left(2\right)}=\left(A^{\left(2\right)}⊗U\right)G_{R_{4}R_{3}\times I_{3}}^{\left(2\right)}$
TD model of $X_{c_{1}}=〚G^{\left(1\right)};A^{\left(1\right)},I_{I_{2}},X_{I_{3}I_{4}\times R2}^{\left(2\right)};R_{1},R_{2}〛\in K^{I_{1}\times I_{2}\times I_{3}I_{4}}$
$X_{I_{2}I_{3}I_{4}\times I_{1}}=[I_{I_{2}}⊗X_{I_{3}I_{4}\times R_{2}}^{\left(2\right)}]G_{I_{2}R_{2}\times R_{1}}^{\left(1\right)}{\left(A^{\left(1\right)}\right)}^{T}$
$X_{I_{1}I_{3}I_{4}\times I_{2}}=(A^{\left(1\right)}⊗X_{I_{3}I_{4}\times R_{2}}^{\left(2\right)})G_{R_{1}R_{2}\times I_{2}}^{\left(1\right)}$
$X_{I_{1}I_{2}\times I_{3}I_{4}}=\left(A^{\left(1\right)}⊗I_{I_{2}}\right)G_{R_{1}I_{2}\times R_{2}}^{\left(1\right)}X_{R_{2}\times I_{3}I_{4}}^{\left(2\right)}=\left(A^{\left(1\right)}⊗I_{I_{2}}\right)G_{R_{1}I_{2}\times R_{2}}^{\left(1\right)}UG_{R_{3}\times I_{3}R_{4}}^{\left(2\right)}{\left(I_{I_{3}}⊗A^{\left(2\right)}\right)}^{T}$
TD model of $X_{c_{2}}=〚G^{\left(2\right)};X_{I_{1}I_{2}\times R_{3}}^{\left(1\right)},I_{I_{3}},A^{\left(2\right)};R_{3},R_{4}〛\in K^{I_{1}I_{2}\times I_{3}\times I_{4}}$
$X_{I_{3}I_{1}I_{2}\times I_{4}}=[I_{I_{3}}⊗X_{I_{1}I_{2}\times R_{3}}^{\left(1\right)}]G_{I_{3}R_{3}\times R_{4}}^{\left(2\right)}{\left(A^{\left(2\right)}\right)}^{T}$
$X_{I_{4}I_{1}I_{2}\times I_{3}}=(A^{\left(2\right)}⊗X_{I_{1}I_{2}\times R_{3}}^{\left(1\right)})G_{R_{4}R_{3}\times I_{3}}^{\left(2\right)}$
$X_{I_{1}I_{2}\times I_{3}I_{4}}=X_{I_{1}I_{2}\times R_{3}}^{\left(1\right)}G_{R_{3}\times I_{3}R_{4}}^{\left(2\right)}{\left(I_{I_{3}}⊗A^{\left(2\right)}\right)}^{T}=\left(A^{\left(1\right)}⊗I_{I_{2}}\right)G_{R_{1}I_{2}\times R_{2}}^{\left(1\right)}UG_{R_{3}\times I_{3}R_{4}}^{\left(2\right)}{\left(I_{I_{3}}⊗A^{\left(2\right)}\right)}^{T}$

**Table 16:** Unfoldings of the NTD-4 model.

NTD-4 model of $X=〚A^{\left(1\right)},G^{\left(1\right)},U,G^{\left(2\right)},A^{\left(2\right)};R_{1},R_{2},R_{3},R_{4}〛\in K^{{\underline{I}}_{4}}$
$A^{\left(1\right)}\in K^{I_{1}\times R_{1}}\;\;\;;\;\;\;G^{\left(1\right)}\in K^{R_{1}\times I_{2}\times R_{2}}\;\;\;;\;\;\;U\in K^{R_{2}\times R_{3}}\;\;;\;\;G^{\left(2\right)}\in K^{R_{3}\times I_{3}\times R_{4}}\;\;;\;A^{\left(2\right)}\in K^{I_{4}\times R_{4}}$
$X_{I_{3}I_{1}I_{2}\times I_{4}}=[I_{I_{3}}⊗\left(A^{\left(1\right)}⊗I_{I_{2}}\right)G_{R_{1}I_{2}\times R_{2}}^{\left(1\right)}U]G_{I_{3}R_{3}\times R_{4}}^{\left(2\right)}{\left(A^{\left(2\right)}\right)}^{T}$
$X_{I_{4}I_{1}I_{2}\times I_{3}}=[A^{\left(2\right)}⊗\left(A^{\left(1\right)}⊗I_{I_{2}}\right)G_{R_{1}I_{2}\times R_{2}}^{\left(1\right)}U]G_{R_{4}R_{3}\times I_{3}}^{\left(2\right)}$
$X_{I_{2}I_{3}I_{4}\times I_{1}}=[I_{I_{2}}⊗\left(I_{I_{3}}⊗A^{\left(2\right)}\right)G_{I_{3}R_{4}\times R_{3}}^{\left(2\right)}U^{T}]G_{I_{2}R_{2}\times R_{1}}^{\left(1\right)}{\left(A^{\left(1\right)}\right)}^{T}$
$X_{I_{1}I_{3}I_{4}\times I_{2}}=[A^{\left(1\right)}⊗\left(I_{I_{3}}⊗A^{\left(2\right)}\right)G_{I_{3}R_{4}\times R_{3}}^{\left(2\right)}U^{T}]G_{R_{1}R_{2}\times I_{2}}^{\left(1\right)}$
$x_{I_{3}I_{4}I_{1}I_{2}}=[\left(I_{I_{3}}⊗A^{\left(2\right)}\right)G_{I_{3}R_{4}\times R_{3}}^{\left(2\right)}⊗\left(A^{\left(1\right)}⊗I_{I_{2}}\right)G_{R_{1}I_{2}\times R_{2}}^{\left(1\right)}]\mathrm{vec}\left(U\right)$

**Table 17:** NTD-6 and NGTD-7 models.

$X\in K^{{\underline{I}}_{6}}$
NTD-6 model
$A^{\left(1\right)}\in K^{I_{1}\times R_{1}}\;\;\;;\;\;\;G^{\left(1\right)}\in K^{R_{1}\times I_{2}\times I_{3}\times R_{2}}\;\;\;;\;\;\;U\in K^{R_{2}\times R_{3}}\;\;;\;\;G^{\left(2\right)}\in K^{R_{3}\times I_{4}\times I_{5}\times R_{4}}\;\;;\;A^{\left(2\right)}\in K^{I_{6}\times R_{4}}$
$x_{{\underline{i}}_{6}}=\sum_{r_{1}=1}^{R_{1}}\sum_{r_{2}=1}^{R_{2}}\sum_{r_{3}=1}^{R_{3}}\sum_{r_{4}=1}^{R_{4}}a_{i_{1},r_{1}}^{\left(1\right)}g_{r_{1},i_{2},i_{3},r_{2}}^{\left(1\right)}u_{r_{2},r_{3}}g_{r_{3},i_{4},i_{5},r_{4}}^{\left(2\right)}a_{i_{6},r_{4}}^{\left(2\right)}$
$X\in K^{{\underline{I}}_{7}}$
NGTD-7 model
$A^{\left(1\right)}\in K^{I_{1}\times I_{2}\times R_{1}}\;\;\;;\;\;\;G^{\left(1\right)}\in K^{R_{1}\times I_{2}\times I_{3}\times I_{4}\times R_{2}}\;\;\;;\;\;\;U\in K^{R_{2}\times I_{2}\times R_{3}}\;\;;\;\;G^{\left(2\right)}\in K^{R_{3}\times I_{2}\times I_{5}\times I_{6}\times R_{4}}\;\;;\;A^{\left(2\right)}\in K^{I_{7}\times R_{4}}$
$x_{{\underline{i}}_{7}}=\sum_{r_{1}=1}^{R_{1}}\sum_{r_{2}=1}^{R_{2}}\sum_{r_{3}=1}^{R_{3}}\sum_{r_{4}=1}^{R_{4}}a_{i_{1},i_{2},r_{1}}^{\left(1\right)}g_{r_{1},i_{2},i_{3},i_{4},r_{2}}^{\left(1\right)}u_{r_{2},i_{2},r_{3}}g_{r_{3},i_{2},i_{5},i_{6},r_{4}}^{\left(2\right)}a_{i_{7},r_{4}}^{\left(2\right)}$

**Table 18:** Closed-form algorithms to estimate the parameters of a NCPD-4 model.

Models	Closed-Form Algorithm 1	Closed-Form Algorithm 2
NCPD-4	$B^{\left(2\right)}⋄X_{I_{1}I_{2}\times R_{2}}^{\left(1\right)}=X_{I_{4}I_{1}I_{2}\times I_{3}}{\left[{\left(A^{\left(2\right)}\right)}^{T}\right]}^{†}$	$A^{\left(1\right)}⋄X_{I_{3}I_{4}\times R_{1}}^{\left(2\right)}=X_{I_{1}I_{3}I_{4}\times I_{2}}{\left[{\left(B^{\left(1\right)}\right)}^{T}\right]}^{†}$
	$\mathrm{KRF}\;\;\Rightarrow \;\;({\hat{B}}^{\left(2\right)},{\hat{X}}_{I_{1}I_{2}\times R_{2}}^{\left(1\right)})$	$\mathrm{KRF}\;\;\Rightarrow \;\;({\hat{A}}^{\left(1\right)},{\hat{X}}_{I_{3}I_{4}\times R_{1}}^{\left(2\right)})$
	$\mathrm{Reshaping}\;\;:\;\;{\hat{X}}_{I_{1}I_{2}\times R_{2}}^{\left(1\right)}\to {\hat{X}}_{I_{1}R_{2}\times I_{2}}^{\left(1\right)}$	$\mathrm{Reshaping}\;\;:\;\;{\hat{X}}_{I_{3}I_{4}\times R_{1}}^{\left(2\right)}\to {\hat{X}}_{I_{4}R_{1}\times I_{3}}^{\left(2\right)}$
	$A^{\left(1\right)}⋄G^{T}={\hat{X}}_{I_{1}R_{2}\times I_{2}}^{\left(1\right)}{\left[{\left(B^{\left(1\right)}\right)}^{T}\right]}^{†}$	$B^{\left(2\right)}⋄G={\hat{X}}_{I_{4}R_{1}\times I_{3}}^{\left(2\right)}{\left[{\left(A^{\left(2\right)}\right)}^{T}\right]}^{†}$
	$\mathrm{KRF}\;\;\Rightarrow \;\;({\hat{A}}^{\left(1\right)},\hat{G})$	$\mathrm{KRF}\;\;\Rightarrow \;\;({\hat{B}}^{\left(2\right)},\hat{G})$

**Table 19:** Closed-form algorithms to estimate the parameters of the NTD-4 model.

Models	Closed-Form Algorithm 1	Closed-Form Algorithm 2
NTD-4	$A^{\left(2\right)}⊗X_{I_{1}I_{2}\times R_{3}}^{\left(1\right)}=\;X_{I_{4}I_{1}I_{2}\times I_{3}}{\left[G_{R_{4}R_{3}\times I_{3}}^{\left(2\right)}\right]}^{†}$	$A^{\left(1\right)}⊗X_{I_{3}I_{4}\times R_{2}}^{\left(2\right)}=\;X_{I_{1}I_{3}I_{4}\times I_{2}}{\left[G_{R_{1}R_{2}\times I_{2}}^{\left(1\right)}\right]}^{†}$
	$\mathrm{KronF}\;\;\Rightarrow \;\;({\hat{A}}^{\left(2\right)},{\hat{X}}_{I_{1}I_{2}\times R_{3}}^{\left(1\right)})$	$\mathrm{KronF}\;\;\Rightarrow \;\;({\hat{A}}^{\left(1\right)},{\hat{X}}_{I_{3}I_{4}\times R_{2}}^{\left(2\right)})$
	$\mathrm{Reshaping}\;\;:\;\;{\hat{X}}_{I_{1}I_{2}\times R_{3}}^{\left(1\right)}\to {\hat{X}}_{I_{1}R_{3}\times I_{2}}^{\left(1\right)}$	$\mathrm{Reshaping}\;\;:\;\;{\hat{X}}_{I_{3}I_{4}\times R_{2}}^{\left(2\right)}\to {\hat{X}}_{I_{4}R_{2}\times I_{3}}^{\left(2\right)}$
	$A^{\left(1\right)}⊗U^{T}={\hat{X}}_{I_{1}R_{3}\times I_{2}}^{\left(1\right)}{\left(G_{R_{1}R_{2}\times I_{2}}^{\left(1\right)}\right)}^{†}$	$A^{\left(2\right)}⊗U={\hat{X}}_{I_{4}R_{2}\times I_{3}}^{\left(2\right)}{\left(G_{R_{4}R_{3}\times I_{3}}^{\left(2\right)}\right)}^{†}$
	$\mathrm{KronF}\;\;\Rightarrow \;\;({\hat{A}}^{\left(1\right)},\hat{U})$	$\mathrm{KronF}\;\;\Rightarrow \;\;({\hat{A}}^{\left(2\right)},\hat{U})$

**Table 20:** Closed-form algorithms to estimate the parameters of the NTD-6 model.

Models	Closed-Form Algorithm 1	Closed-Form Algorithm 2
NTD-6	$A^{\left(2\right)}⊗X_{I_{1}I_{2}I_{3}\times R_{3}}^{\left(1\right)}=\;X_{I_{6}I_{1}I_{2}I_{3}\times I_{4}I_{5}}{\left[G_{R_{4}R_{3}\times I_{4}I_{5}}^{\left(2\right)}\right]}^{†}$	$A^{\left(1\right)}⊗X_{I_{4}I_{5}I_{6}\times R_{2}}^{\left(2\right)}=\;X_{I_{1}I_{4}I_{5}I_{6}\times I_{2}I_{3}}{\left[G_{R_{1}R_{2}\times I_{2}I_{3}}^{\left(1\right)}\right]}^{†}$
	$\mathrm{KronF}\;\;\Rightarrow \;\;({\hat{A}}^{\left(2\right)},{\hat{X}}_{I_{1}I_{2}I_{3}\times R_{3}}^{\left(1\right)})$	$\mathrm{KronF}\;\;\Rightarrow \;\;({\hat{A}}^{\left(1\right)},{\hat{X}}_{I_{4}I_{5}I_{6}\times R_{2}}^{\left(2\right)})$
	$\mathrm{Reshaping}\;\;:\;\;{\hat{X}}_{I_{1}I_{2}I_{3}\times R_{3}}^{\left(1\right)}\to {\hat{X}}_{I_{1}R_{3}\times I_{2}I_{3}}^{\left(1\right)}$	$\mathrm{Reshaping}\;\;:\;\;{\hat{X}}_{I_{4}I_{5}I_{6}\times R_{2}}^{\left(2\right)}\to {\hat{X}}_{I_{6}R_{2}\times I_{4}I_{5}}^{\left(2\right)}$
	$A^{\left(1\right)}⊗U^{T}={\hat{X}}_{I_{1}R_{3}\times I_{2}I_{3}}^{\left(1\right)}{\left(G_{R_{1}R_{2}\times I_{2}I_{3}}^{\left(1\right)}\right)}^{†}$	$A^{\left(2\right)}⊗U={\hat{X}}_{I_{6}R_{2}\times I_{4}I_{5}}^{\left(2\right)}{\left(G_{R_{4}R_{3}\times I_{4}I_{5}}^{\left(2\right)}\right)}^{†}$
	$\mathrm{KronF}\;\;\Rightarrow \;\;({\hat{A}}^{\left(1\right)},\hat{U})$	$\mathrm{KronF}\;\;\Rightarrow \;\;({\hat{A}}^{\left(2\right)},\hat{U})$

**Table 21:** Closed-form algorithms to estimate the parameters of the NGTD-7 model.

Closed-Form Algorithms 1	Closed-Form Algorithms 2
$A^{\left(2\right)}⊗{\mathrm{bdiag}}_{i_{2}}\left(X_{I_{1}I_{3}I_{4}\times R_{3}}^{\left(1\right)}\right)={\mathrm{bdiag}}_{i_{2}}\left(X_{I_{7}I_{1}I_{3}I_{4}\times I_{5}I_{6}}\right){\mathrm{bdiag}}_{i_{2}}{\left(G_{R_{4}R_{3}\times I_{5}I_{6}}^{\left(2\right)}\right)}^{†}$	${\mathrm{bdiag}}_{i_{2}}\left[A_{I_{1}\times R_{1}}^{\left(1\right)}⊗X_{I_{5}I_{6}I_{7}\times R_{2}}^{\left(2\right)}\right]={\mathrm{bdiag}}_{i_{2}}\left(X_{I_{1}I_{5}I_{6}I_{7}\times I_{3}I_{4}}\right){\mathrm{bdiag}}_{i_{2}}{\left(G_{R_{1}R_{2}\times I_{3}I_{4}}^{\left(1\right)}\right)}^{†}$
$\mathrm{KronF}\;\;\Rightarrow \;\;\left({\hat{A}}^{\left(2\right)},{\mathrm{bdiag}}_{i_{2}}\left({\hat{X}}_{I_{1}I_{3}I_{4}\times R_{3}}^{\left(1\right)}\right)\right)$	$\mathrm{KronF}\;\;\Rightarrow \;\;\left({\mathrm{bdiag}}_{i_{2}}\left({\hat{A}}_{I_{1}\times R_{1}}^{\left(1\right)}\right),{\mathrm{bdiag}}_{i_{2}}\left({\hat{X}}_{I_{5}I_{6}I_{7}\times R_{2}}^{\left(2\right)}\right)\right)$
$\mathrm{Reshaping}\;\;:\;\;{\mathrm{bdiag}}_{i_{2}}\left({\hat{X}}_{I_{1}I_{3}I_{4}\times R_{3}}^{\left(1\right)}\right)\to {\mathrm{bdiag}}_{i_{2}}\left({\hat{X}}_{I_{1}R_{3}\times I_{3}I_{4}}^{\left(1\right)}\right)$	$\mathrm{Reshaping}\;\;:\;\;{\mathrm{bdiag}}_{i_{2}}\left({\hat{X}}_{I_{5}I_{6}I_{7}\times R_{2}}^{\left(2\right)}\right)\to {\mathrm{bdiag}}_{i_{2}}\left(X_{I_{7}R_{2}\times I_{5}I_{6}}^{\left(2\right)}\right)$
${\mathrm{bdiag}}_{i_{2}}\left[A_{I_{1}\times R_{1}}^{\left(1\right)}⊗U_{R_{3}\times R_{2}}\right]={\mathrm{bdiag}}_{i_{2}}\left({\hat{X}}_{I_{1}R_{3}\times I_{3}I_{4}}^{\left(1\right)}\right){\mathrm{bdiag}}_{i_{2}}{\left(G_{R_{1}R_{2}\times I_{3}I_{4}}^{\left(1\right)}\right)}^{†}$	$A^{\left(2\right)}⊗{\mathrm{bdiag}}_{i_{2}}\left(U_{R_{2}\times R_{3}}\right)={\mathrm{bdiag}}_{i_{2}}\left(X_{I_{7}R_{2}\times I_{5}I_{6}}^{\left(2\right)}\right){\mathrm{bdiag}}_{i_{2}}(G_{R_{4}R_{3}\times I_{5}I_{6}}^{\left(2\right)}{)}^{†}$
$\mathrm{KRF}\;\;\Rightarrow \;\;\left({\mathrm{bdiag}}_{i_{2}}\left({\hat{A}}_{I_{1}\times R_{1}}^{\left(1\right)}\right),{\mathrm{bdiag}}_{i_{2}}\left({\hat{U}}_{R_{3}\times R_{2}}\right)\right)$	$\mathrm{KRF}\;\;\Rightarrow \;\;\left({\hat{A}}^{\left(2\right)},{\mathrm{bdiag}}_{i_{2}}\left({\hat{U}}_{R_{2}\times R_{3}}\right)\right)$

**Table 22:** Overview of cooperative systems.

Ref.	OFDM/mmW	Relay/IRS/UAV	Coding/Training	Tensor Models	Receiver Algorithms
[53]		Two-hop relay	Simplified KRST	CPD-PARATUCK	ALS + KRF
[40]		Two-hop relay	Simplified KRST	NCPD	ALS
[54]		Two-hop relay	Simplified KRST	NCPD	KRF
[41]		Two-hop relay	TST	NTD	ALS, KronF
[55]		Two-hop relay	MKRST	NCPD	KRF
			MKronST		
[56]		Two-hop relay	Matrices + Training	Tucker-2	KRF + Structured LS
[57]		Two-hop relay	TST	Block Tucker-2	KronF
[58]		Multi-hop relay	Simplified KRST	Generalized NCPD	KRF
[59]		Three-hop relay	KRST	NCPD	ALS + KRF
[60]		Two-hop relay	Matrices + Training	CPD	ALS, MMSE
[61]		Three-hop relay	TST-CPD	NTD	Coupled SVD, ALS
[42]		Two-hop relay	TST	Coupled NTD	KronF
[62]		Three-hop relay	Matrices + Training	CPD + structured Tucker	ALS
[63]		Two-hop relay	TST	Block Tucker2-CPD	ALS, KronF
[64]	OFDM/mmW	Two-hop relay	Matrices + Training	Structured CPD	SS * + ESPRIT
[65]	OFDM/mmW	IRS	Matrices + Training	CPD	Tensor completion
[66]	mmW	Two-hop relay	Matrices + Training	CPD	ALS, KRF
[67]	mmW	One-hop	Simplified KRST	NCPD	SVP *-ALS
[68,69]		IRS	Training	CPD	ALS
[70]		UAV	Simplified KRST	NCPD	KRF-ALM *
			+ Training		
[71]		UAV-IRS	Simplified KRST	CPD	ALS + KRF
[72]		Two-hop relay	TST + Training	Tucker-2	LM * + LMMSE *
[73]	OFDM	Two-hop relay	TST + simplified TSTF	Coupled NTD	KronF
[74]	OFDM	Two-hop relay	KRSTF	NCPD	ALS

* SS = spatial smoothing; SVP = singular value projection; LM = Levenberg–Marquardt; ALM = Accelerated LM; LMMSE = linear minimum mean-square error.

**Table 23:** Design parameters and system matrices and tensors.

Design Parameters	Definitions		
$M_{S}$, $M_{T}$	Numbers of transmit antennas at the source and relay nodes		
$M_{R}$, $M_{D}$	Numbers of receive antennas at the relay and destination nodes		
*N*	Number of symbols per data stream		
*R*	Number of data streams		
*F*	Number of subcarriers		
$P_{S}$, $P_{R}$	Time-spreading lengths at source and relay		
$J_{S}$, $J_{R}$	Numbers of chips at source and relay		
**Matrices/Tensors**	**Definitions**	**Dimensions**	**Codings**
S	Symbol matrix	N×R	TSTF, TST, STSTF, STST, DKRSTF, STST-MSMKron
S	Symbol matrix	$N\times M_{S}$	SKRST, SKRST-MSMKR
$H^{\left(SR\right)}$	Source–relay channel tensor	$M_{R}\times F\times M_{S}$	TSTF, STSTF
$H^{\left(RD\right)}$	Relay–destination channel tensor	$M_{D}\times F\times M_{T}$	TSTF, STSTF
$H^{\left(SR\right)}$	Source–relay channel matrix	$M_{R}\times M_{S}$	TST, STST, SKRST, DKRSTF,
			STST-MSMKron, SKRST-MSMKR
$H^{\left(RD\right)}$	Relay–destination channel matrix	$M_{D}\times M_{T}$	TST, STST, DKRSTF
$H^{\left(RD\right)}$	Relay–destination channel matrix	$M_{D}\times M_{R}$	SKRST
$H^{\left(RD\right)}$	Relay–destination channel matrix	$M_{D}\times M_{S}$	STST-MSMKron, SKRST-MSMKR
$C^{\left(S\right)}$	Source-coding tensor	$M_{S}\times F\times P_{S}\times J_{S}\times R$	TSTF
$C^{\left(S\right)}$	Source-coding tensor	$M_{S}\times F\times P_{S}\times R$	STSTF
$C^{\left(S\right)}$	Source-coding tensor	$M_{S}\times P_{S}\times J_{S}\times R$	TST
$C^{\left(S\right)}$	Source-coding tensor	$M_{S}\times P_{S}\times R$	STST
$C^{\left(S\right)}$	Source-coding tensor	$M_{S}\times P_{S}\times R_{1}\cdots \times R_{Q}$	STST-MSMKron
$C^{\left(R\right)}$	Relay-coding tensor	$M_{T}\times F\times P_{R}\times J_{R}\times M_{R}$	TSTF
$C^{\left(R\right)}$	Relay-coding tensor	$M_{T}\times F\times P_{R}\times M_{R}$	STSTF
$C^{\left(R\right)}$	Relay-coding tensor	$M_{T}\times P_{R}\times J_{R}\times M_{R}$	TST
$C^{\left(R\right)}$	Relay-coding tensor	$M_{T}\times P_{R}\times M_{R}$	STST
$C^{\left(R\right)}$	Relay-coding tensor	$M_{S}\times P_{R}\times R_{1}\cdots \times R_{Q}$	STST-MSMKron
$C^{\left(S\right)}$	Source space-time coding matrix	$P_{S}\times M_{S}$	SKRST, DKRSTF, SKRST-MSMKR
$C^{\left(R\right)}$	Relay space-time coding matrix	$P_{R}\times M_{R}$	SKRST, DKRSTF
$C^{\left(R\right)}$	Relay space-time coding matrix	$P_{R}\times M_{S}$	SKRST-MSMKR
$W^{\left(S\right)}$	Source space-coding matrix	$M_{S}\times R$	DKRSTF
$W^{\left(R\right)}$	Relay space-coding matrix	$M_{T}\times M_{R}$	DKRSTF
$A^{\left(S\right)}$	Source frequency-coding matrix	F×R	DKRSTF

**Table 24:** Two-hop system with DKRSTF codings.

Coded and Received Signals	Symbols/Codings	Channels	Encoded/Received Signals	Dimensions
	$S\in C^{N\times R}$			
		**First hop**		
**Signals coded at source**	$A^{\left(S\right)}$, $C^{\left(S\right)}$, $W^{\left(S\right)}$		$V_{FN\times M_{S}}^{\left(S\right)}=\left(A^{\left(S\right)}⋄S\right){W^{\left(S\right)}}^{T}$	$M_{S}\times F\times N$
			$U_{P_{S}FN\times M_{S}}^{\left(S\right)}=C^{\left(S\right)}⋄V_{FN\times M_{S}}^{\left(S\right)}$	$M_{S}\times P_{S}\times F\times N$
**Signals received at relay**		$H^{\left(SR\right)}$	$X_{M_{R}\times P_{S}FN}^{\left(R\right)}=H^{\left(SR\right)}U_{M_{S}\times P_{S}FN}^{\left(S\right)}$	$M_{R}\times P_{S}\times F\times N$
		**Second hop**		
**Signals coded at relay**	$C^{\left(R\right)}$, $W^{\left(R\right)}$		$U_{P_{R}P_{S}FN\times M_{T}}^{\left(R\right)}=\left(C^{\left(R\right)}⋄X_{P_{S}FN\times M_{R}}^{\left(R\right)}\right){W^{\left(R\right)}}^{T}$	$M_{T}\times P_{R}\times P_{S}\times F\times N$
**Signals received at destination**		$H^{\left(RD\right)}$	$X_{M_{D}\times P_{R}P_{S}FN}^{\left(D\right)}=H^{\left(RD\right)}U_{M_{T}\times P_{R}P_{S}FN}^{\left(R\right)}$	$M_{D}\times P_{R}\times P_{S}\times F\times N$

**Table 25:** Tensors of encoded and received signals for two-hop relay systems.

Systems	Tensor Writing	Scalar Writing	Models of $X^{\left(D\right)}$
**Tensor-based codings-AF protocol**			
TSTF	$U^{\left(S\right)}=C^{\left(S\right)}\times_{5}S\in C^{M_{S}\times F\times P_{S}\times J_{S}\times N}$	$u_{m_{S},f,p_{S},j_{S},n}^{\left(S\right)}=\sum_{r}c_{m_{S},f,p_{S},j_{S},r}^{\left(S\right)}s_{n,r}$	
	$X^{\left(R\right)}=C^{\left(S\right)}\times_{1}^{3}H^{\left(SR\right)}\times_{5}S\in C^{M_{R}\times F\times P_{S}\times J_{S}\times N}$	$x_{m_{R},f,p_{S},j_{S},n}^{\left(R\right)}=\sum_{m_{S}}\sum_{r}h_{m_{R},f,m_{S}}^{\left(SR\right)}c_{m_{S},f,p_{S},j_{S},r}^{\left(S\right)}s_{n,r}$	NGTD-7
	$X^{\left(D\right)}=C^{\left(R\right)}\times_{1}^{3}H^{\left(RD\right)}\times_{5}^{1}X^{\left(R\right)}\in C^{M_{D}\times F\times P_{R}\times J_{R}\times P_{S}\times J_{S}\times N}$	$x_{m_{D},f,p_{R},j_{R},p_{S},j_{S},n}^{\left(D\right)}=\sum_{m_{T}}\sum_{m_{R}}h_{m_{D},f,m_{T}}^{\left(RD\right)}c_{m_{T},f,p_{R},j_{R},m_{R}}^{\left(R\right)}x_{m_{R},f,p_{S},j_{S},n}^{\left(R\right)}$	
TST	$U^{\left(S\right)}=C^{\left(S\right)}\times_{4}S\in C^{M_{S}\times P_{S}\times J_{S}\times N}$	$u_{m_{S},p_{S},j_{S},n}^{\left(S\right)}=\sum_{r}c_{m_{S},p_{S},j_{S},r}^{\left(S\right)}s_{n,r}$	
	$X^{\left(R\right)}=C^{\left(S\right)}\times_{1}H^{\left(SR\right)}\times_{4}S\in C^{M_{R}\times P_{S}\times J_{S}\times N}$	$x_{m_{R},p_{S},j_{S},n}^{\left(R\right)}=\sum_{m_{S}}\sum_{r}h_{m_{R},m_{S}}^{\left(SR\right)}c_{m_{S},p_{S},j_{S},r}^{\left(S\right)}s_{n,r}$	NTD-6
	$X^{\left(D\right)}=C^{\left(R\right)}\times_{1}H^{\left(RD\right)}\times_{4}^{1}X^{\left(R\right)}\in C^{M_{D}\times P_{R}\times J_{R}\times P_{S}\times J_{S}\times N}$	$x_{m_{D},p_{R},j_{R},p_{S},j_{S},n}^{\left(D\right)}=\sum_{m_{T}}\sum_{m_{R}}h_{m_{D},m_{T}}^{\left(RD\right)}c_{m_{T},p_{R},j_{R},m_{R}}^{\left(R\right)}x_{m_{R},p_{S},j_{S},n}^{\left(R\right)}$	
STSTF	$U^{\left(S\right)}=C^{\left(S\right)}\times_{4}S\in C^{M_{S}\times F\times P_{S}\times N}$	$u_{m_{S},f,p_{S},n}^{\left(S\right)}=\sum_{r}c_{m_{S},f,p_{S},r}^{\left(S\right)}s_{n,r}$	
	$X^{\left(R\right)}=C^{\left(S\right)}\times_{1}^{3}H^{\left(SR\right)}\times_{4}S\in C^{M_{R}\times F\times P_{S}\times N}$	$x_{m_{R},f,p_{S},n}^{\left(R\right)}=\sum_{m_{S}}\sum_{r}h_{m_{R},f,m_{S}}^{\left(SR\right)}c_{m_{S},f,p_{S},r}^{\left(S\right)}s_{n,r}$	NGTD-5
	$X^{\left(D\right)}=C^{\left(R\right)}\times_{1}^{3}H^{\left(RD\right)}\times_{4}^{1}X^{\left(R\right)}\in C^{M_{D}\times F\times P_{R}\times P_{S}\times N}$	$x_{m_{D},f,p_{R},p_{S},n}^{\left(D\right)}=\sum_{m_{T}}\sum_{m_{R}}h_{m_{D},f,m_{T}}^{\left(RD\right)}c_{m_{T},f,p_{R},m_{R}}^{\left(R\right)}x_{m_{R},f,p_{S},n}^{\left(R\right)}$	
STST	$U^{\left(S\right)}=C^{\left(S\right)}\times_{3}S\in C^{M_{S}\times P_{S}\times N}$	$u_{m_{S},p_{S},n}^{\left(S\right)}=\sum_{r}c_{m_{S},p_{S},r}^{\left(S\right)}s_{n,r}$	
	$X^{\left(R\right)}=C^{\left(S\right)}\times_{1}H^{\left(SR\right)}\times_{3}S\in C^{M_{R}\times P_{S}\times N}$	$x_{m_{R},p_{S},n}^{\left(R\right)}=\sum_{m_{S}}\sum_{r}h_{m_{R},m_{S}}^{\left(SR\right)}c_{m_{S},p_{S},r}^{\left(S\right)}s_{n,r}$	NTD-4
	$X^{\left(D\right)}=C^{\left(R\right)}\times_{1}H^{\left(RD\right)}\times_{3}^{1}X^{\left(R\right)}\in C^{M_{D}\times P_{R}\times P_{S}\times N}$	$x_{m_{D},p_{R},p_{S},n}^{\left(D\right)}=\sum_{m_{T}}\sum_{m_{R}}h_{m_{D},m_{T}}^{\left(RD\right)}c_{m_{T},p_{R},m_{R}}^{\left(R\right)}x_{m_{R},p_{S},n}^{\left(R\right)}$	
**Matrix-based codings-AF protocol**			
DKRSTF	$V^{\left(S\right)}=I_{R}\times_{1}W^{\left(S\right)}\times_{2}A^{\left(S\right)}\times_{3}S\in C^{M_{S}\times F\times N}$	$v_{m_{S},f,n}^{\left(S\right)}=\sum_{r}w_{m_{S},r}^{\left(S\right)}a_{f,r}^{\left(S\right)}s_{n,r}$	
	$X_{c}^{\left(R\right)}=\;I_{M_{S}}\times_{1}H^{\left(SR\right)}\times_{2}C^{\left(S\right)}\times_{3}V_{FN\times M_{S}}^{\left(S\right)}\in C^{M_{R}\times P_{S}\times FN}$	$x_{m_{R},p_{S},f,n}^{\left(R\right)}=\sum_{m_{S}}h_{m_{R},m_{S}}^{\left(SR\right)}c_{p_{S},m_{S}}^{\left(S\right)}v_{m_{S},f,n}$	NCPD-5
	$X_{c}^{\left(D\right)}=I_{M_{R}}\times_{1}H^{\left(RD\right)}W^{\left(R\right)}\times_{2}C^{\left(R\right)}\;\;\;\;\;\;\;\;\;\;\;\;\;\;\;\;\;\;\;\;\;\;\;\;\;\;\;\;\;\;$	$x_{m_{D},p_{R},p_{S},f,n}^{\left(D\right)}=\sum_{m_{R}}\left(\sum_{m_{T}}h_{m_{D},m_{T}}^{\left(RD\right)}w_{m_{T},m_{R}}^{\left(R\right)}\right)c_{p_{R},m_{R}}^{\left(R\right)}x_{m_{R},p_{S},f,n}^{\left(R\right)}$	
	$\;\;\;\;\;\;\times_{3}X_{P_{S}FN\times M_{R}}^{\left(R\right)}\in C^{M_{D}\times P_{R}\times P_{S}FN}$		
SKRST	$U^{\left(S\right)}=I_{M_{S}}\times_{2}C^{\left(S\right)}\times_{3}S\in C^{M_{S}\times P_{S}\times N}$	$u_{m_{S},p_{S},n}^{\left(S\right)}=c_{p_{S},m_{S}}^{\left(S\right)}s_{n,m_{S}}$	
	$X^{\left(R\right)}=I_{M_{S}}\times_{1}H^{\left(SR\right)}\times_{2}C^{\left(S\right)}\times_{3}S\in C^{M_{R}\times P_{S}\times N}$	$x_{m_{R},p_{S},n}^{\left(R\right)}=\sum_{m_{S}}h_{m_{R},m_{S}}^{\left(SR\right)}c_{p_{S},m_{S}}^{\left(S\right)}s_{n,m_{S}}$	NCPD-4
	$X^{\left(D\right)}=I_{M_{R}}\times_{1}H^{\left(RD\right)}\times_{2}C^{\left(R\right)}\times_{3}^{1}X^{\left(R\right)}\in C^{M_{D}\times P_{R}\times P_{S}\times N}$	$x_{m_{D},p_{R},p_{S},n}^{\left(D\right)}=\sum_{m_{R}}h_{m_{D},m_{R}}^{\left(RD\right)}c_{p_{R},m_{R}}^{\left(R\right)}x_{m_{R},p_{S},n}^{\left(R\right)}$	
**Combined codings-DF protocol**			
STST-MSMKron	$U_{c}^{\left(S\right)}=C^{\left(S\right)}\times_{3}S\in C^{M_{S}\times P_{S}\times N_{1}\cdots N_{Q}}$	$u_{m_{S},p_{S},n_{1},...,n_{Q}}^{\left(S\right)}=\sum_{r_{1}}\cdots \sum_{r_{Q}}c_{m_{S},p_{S},r_{1},...,r_{Q}}^{\left(S\right)}\;\prod_{q=1}^{Q}s_{n_{q},r_{q}}^{\left(q\right)}$	
$S=\overset{Q}{\underset{q=1}{⊗}}S^{\left(q\right)}$	$X_{c}^{\left(R\right)}=C^{\left(S\right)}\times_{1}H^{\left(SR\right)}\times_{3}S\in C^{M_{R}\times P_{S}\times N_{1}\cdots N_{Q}}$	$x_{m_{R},p_{S},n_{1},...,n_{Q}}^{\left(R\right)}=\sum_{m_{S}}\sum_{r_{1}}\cdots \sum_{r_{Q}}h_{m_{R},m_{S}}^{\left(SR\right)}c_{m_{S},p_{S},r_{1},...,r_{Q}}^{\left(S\right)}\;\prod_{q=1}^{Q}s_{n_{q},r_{q}}^{\left(q\right)}$	TD-(Q+2)
	$X_{c}^{\left(D\right)}=C^{\left(R\right)}\times_{1}H^{\left(RD\right)}\times_{3}\hat{S}\in C^{M_{D}\times P_{R}\times N_{1}\cdots N_{Q}}$	$x_{m_{D},p_{R},n_{1},...,n_{Q}}^{\left(D\right)}=\sum_{m_{S}}\sum_{r_{1}}\cdots \sum_{r_{Q}}h_{m_{D},m_{S}}^{\left(RD\right)}c_{m_{S},p_{R},r_{1},...,r_{Q}}^{\left(R\right)}\;\prod_{q=1}^{Q}{\hat{s}}_{n_{q},r_{q}}^{\left(q\right)}$	
SKRST-MSMKR	$U_{c}^{\left(S\right)}=I_{M_{S}}\times_{2}C^{\left(S\right)}\times_{3}S\in C^{M_{S}\times P_{S}\times N_{1}\cdots N_{Q}}$	$u_{m_{S},p_{S},n_{1},...,n_{Q}}^{\left(S\right)}=c_{p_{S},m_{S}}^{\left(S\right)}\;\prod_{q=1}^{Q}s_{n_{q},m_{S}}^{\left(q\right)}$	
$S=\overset{Q}{\underset{q=1}{⋄}}S^{\left(q\right)}$	$X_{c}^{\left(R\right)}=I_{M_{S}}\times_{1}H^{\left(SR\right)}\times_{2}C^{\left(S\right)}\times_{3}S\in C^{M_{R}\times P_{S}\times N_{1}\cdots N_{Q}}$	$x_{m_{R},p_{S},n_{1},...,n_{Q}}^{\left(R\right)}=\sum_{m_{S}}h_{m_{R},m_{S}}^{\left(SR\right)}c_{p_{S},m_{S}}^{\left(S\right)}\;\prod_{q=1}^{Q}s_{n_{q},m_{S}}^{\left(q\right)}$	CPD-(Q+2)
	$X_{c}^{\left(D\right)}=I_{M_{S}}\times_{1}H^{\left(RD\right)}\times_{2}C^{\left(R\right)}\times_{3}\hat{S}\in C^{M_{D}\times P_{R}\times N_{1}\cdots N_{Q}}$	$x_{m_{D},p_{R},n_{1},...,n_{Q}}^{\left(D\right)}=\sum_{m_{S}}h_{m_{D},m_{S}}^{\left(RD\right)}c_{p_{R},m_{S}}^{\left(R\right)}\;\prod_{q=1}^{Q}{\hat{s}}_{n_{q},m_{S}}^{\left(q\right)}$	

**Table 26:** Correspondences between $X^{\left(D\right)}$ and generic nested tensor models.

Tensor-Based Codings																	
**Models**	$X^{\left(1\right)}$	$A^{\left(1\right)}/A^{\left(1\right)}$	$G^{\left(1\right)}$	U/U	$G^{\left(2\right)}$	$A^{\left(2\right)}$	$I_{1}$	$I_{2}$	$I_{3}$	$I_{4}$	$I_{5}$	$I_{6}$	$I_{7}$	$R_{1}$	$R_{2}$	$R_{3}$	$R_{4}$
TSTF/NGTD-7	$H^{\left(SRD\right)}$	$H^{\left(RD\right)}$	$C^{\left(R\right)}$	$H^{\left(SR\right)}$	$C^{\left(S\right)}$	S	$M_{D}$	*F*	$P_{R}$	$J_{R}$	$P_{S}$	$J_{S}$	*N*	$M_{T}$	$M_{R}$	$M_{S}$	*R*
TST/NTD-6	$H^{\left(SRD\right)}$	$H^{\left(RD\right)}$	$C^{\left(R\right)}$	$H^{\left(SR\right)}$	$C^{\left(S\right)}$	S	$M_{D}$	$P_{R}$	$J_{R}$	$P_{S}$	$J_{S}$	*N*	-	$M_{T}$	$M_{R}$	$M_{S}$	*R*
STSTF/NGTD-5	$H^{\left(SRD\right)}$	$H^{\left(RD\right)}$	$C^{\left(R\right)}$	$H^{\left(SR\right)}$	$C^{\left(S\right)}$	S	$M_{D}$	*F*	$P_{R}$	$P_{S}$	*N*	-	-	$M_{T}$	$M_{R}$	$M_{S}$	*R*
STST/NTD-4	$H^{\left(SRD\right)}$	$H^{\left(RD\right)}$	$C^{\left(R\right)}$	$H^{\left(SR\right)}$	$C^{\left(S\right)}$	S	$M_{D}$	$P_{R}$	$P_{S}$	*N*	-	-	-	$M_{T}$	$M_{R}$	$M_{S}$	*R*
**Matrix-Based Codings**																	
**Models**	$X^{\left(1\right)}$	$A^{\left(1\right)}$	$B^{\left(1\right)}$	G	$A^{\left(2\right)}$	$B^{\left(2\right)}$	$I_{1}$	$I_{2}$	$I_{3}$	$I_{4}$	-	-	-	$R_{1}$	$R_{2}$	-	-
SKRST/NCPD-4	$H^{\left(SRD\right)}$	$H^{\left(RD\right)}$	$C^{\left(R\right)}$	$H^{\left(SR\right)}$	$C^{\left(S\right)}$	S	$M_{D}$	$P_{R}$	$P_{S}$	*N*	-	-	-	$M_{R}$	$M_{S}$	-	-

**Table 27:** Closed-form receiver for SKRST and STST systems.

System/Receiver	Closed-Form Receiver 1	Closed-Form Receiver 2
SKRST/KRF	$S⋄H_{M_{D}P_{R}\times M_{S}}^{\left(SRD\right)}=X_{NM_{D}P_{R}\times P_{S}}^{\left(D\right)}{\left[{\left(C^{\left(S\right)}\right)}^{T}\right]}^{†}$	$H^{\left(RD\right)}⋄X_{P_{S}N\times M_{R}}^{\left(R\right)}=X_{M_{D}P_{S}N\times P_{R}}^{\left(D\right)}{\left[{\left(C^{\left(R\right)}\right)}^{T}\right]}^{†}$
	$\mathrm{KRF}\;\;\Rightarrow \;\;(\hat{S},{\hat{H}}_{M_{D}P_{R}\times M_{S}}^{\left(SRD\right)})$	$\mathrm{KRF}\;\;\Rightarrow \;\;({\hat{H}}^{\left(RD\right)},{\hat{X}}_{P_{S}N\times M_{R}}^{\left(R\right)})$
	$\mathrm{Reshaping}\;\;:\;\;{\hat{H}}_{M_{D}P_{R}\times M_{S}}^{\left(SRD\right)}\to {\hat{H}}_{M_{D}M_{S}\times P_{R}}^{\left(SRD\right)}$	$\mathrm{Reshaping}\;\;:\;\;{\hat{X}}_{P_{S}N\times M_{R}}^{\left(R\right)}\to {\hat{X}}_{NM_{R}\times P_{S}}^{\left(R\right)}$
	$H^{\left(\mathrm{RD}\right)}⋄{H^{\left(SR\right)}}^{T}={\hat{H}}_{M_{D}M_{S}\times P_{R}}^{\left(SRD\right)}{\left[{\left(C^{\left(R\right)}\right)}^{T}\right]}^{†}$	$S⋄H^{\left(SR\right)}={\hat{X}}_{NM_{R}\times P_{S}}^{\left(R\right)}{\left[{\left(C^{\left(S\right)}\right)}^{T}\right]}^{†}$
	$\mathrm{KRF}\;\;\Rightarrow \;\;({\hat{H}}^{\left(RD\right)},{\hat{H}}^{\left(SR\right)})$	$\mathrm{KRF}\;\;\Rightarrow \;\;(\hat{S},{\hat{H}}^{\left(SR\right)})$
STST/KronF	$S⊗H_{M_{D}P_{R}\times M_{S}}^{\left(SRD\right)}=\;X_{NM_{D}P_{R}\times P_{S}}^{\left(D\right)}{\left[C_{RM_{S}\times P_{S}}^{\left(S\right)}\right]}^{†}$	$H^{\left(RD\right)}⊗X_{P_{S}N\times M_{R}}^{\left(R\right)}=\;X_{M_{D}P_{S}N\times P_{R}}^{\left(D\right)}{\left[C_{M_{T}M_{R}\times P_{R}}^{\left(R\right)}\right]}^{†}$
	$\mathrm{KronF}\;\;\Rightarrow \;\;(\hat{S},{\hat{H}}_{M_{D}P_{R}\times M_{S}}^{\left(SRD\right)})$	$\mathrm{KronF}\;\;\Rightarrow \;\;({\hat{H}}^{\left(RD\right)},{\hat{X}}_{P_{S}N\times M_{R}}^{\left(R\right)})$
	$\mathrm{Reshaping}\;\;:\;\;{\hat{H}}_{M_{D}P_{R}\times M_{S}}^{\left(SRD\right)}\to {\hat{H}}_{M_{D}M_{S}\times P_{R}}^{\left(SRD\right)}$	$\mathrm{Reshaping}\;\;:\;\;{\hat{X}}_{P_{S}N\times M_{R}}^{\left(R\right)}\to {\hat{X}}_{NM_{R}\times P_{S}}^{\left(R\right)}$
	$H^{\left(RD\right)}⊗{H^{\left(SR\right)}}^{T}={\hat{H}}_{M_{D}M_{S}\times P_{R}}^{\left(SRD\right)}{\left(C_{M_{T}M_{R}\times P_{R}}^{\left(R\right)}\right)}^{†}$	$S⊗H^{\left(SR\right)}={\hat{X}}_{NM_{R}\times P_{S}}^{\left(R\right)}{\left(C_{RM_{S}\times P_{S}}^{\left(S\right)}\right)}^{†}$
	$\mathrm{KronF}\;\;\Rightarrow \;\;({\hat{H}}^{\left(RD\right)},{\hat{H}}^{\left(SR\right)})$	$\mathrm{KronF}\;\;\Rightarrow \;\;(\hat{S},{\hat{H}}^{\left(SR\right)})$

**Table 28:** Semi-blind receivers.

**Tensor-Based codings—AF protocol**			
**System**	**Unfoldings**	**Estimated parameters**	**Corresp.**
TSTF			*NGTD-7*
	${\mathrm{bdiag}}_{f}\left(X_{NM_{D}P_{R}J_{R}\times P_{S}J_{S}}^{\left(D\right)}\left(f\right)\right)=\left[S⊗{\mathrm{bdiag}}_{f}\left(H_{M_{D}P_{R}J_{R}\times M_{S}}^{\left(SRD\right)}\left(f\right)\right)\right]{\mathrm{bdiag}}_{f}\left(C_{RM_{S}\times P_{S}J_{S}}^{\left(S\right)}\left(f\right)\right)$		Equation (52)
	${\mathrm{bdiag}}_{f}\left(H_{M_{D}M_{S}\times P_{R}J_{R}}^{\left(SRD\right)}\left(f\right)\right)={\mathrm{bdiag}}_{f}\left(H_{M_{D}\times M_{T}}^{\left(RD\right)}\left(f\right)⊗H_{M_{S}\times M_{R}}^{\left(SR\right)}\left(f\right)\right){\mathrm{bdiag}}_{f}\left(C_{M_{T}M_{R}\times P_{R}J_{R}}^{\left(R\right)}\left(f\right)\right)$		Equation (49)
**Receiver**	**Estimation steps**		
KronF	$S⊗{\mathrm{bdiag}}_{f}\left(H_{M_{D}P_{R}J_{R}\times M_{S}}^{\left(SRD\right)}\left(f\right)\right)={\mathrm{bdiag}}_{f}\left(X_{NM_{D}P_{R}J_{R}\times P_{S}J_{S}}^{\left(D\right)}\left(f\right)\right){\mathrm{bdiag}}_{f}{\left(C_{P_{S}J_{S}\times RM_{S}}^{\left(S\right)}\left(f\right)\right)}^{*}$	$\hat{S}\;,\;{\mathrm{bdiag}}_{f}\left({\hat{H}}_{M_{D}P_{R}J_{R}\times M_{S}}^{\left(SRD\right)}\left(f\right)\right)$	Table 21
	$\mathrm{Reshaping}\;\;{\mathrm{bdiag}}_{f}\left({\hat{H}}_{M_{D}P_{R}J_{R}\times M_{S}}^{\left(SRD\right)}\left(f\right)\right)\to {\mathrm{bdiag}}_{f}\left({\hat{H}}_{M_{D}M_{S}\times P_{R}J_{R}}^{\left(SRD\right)}\left(f\right)\right)$		*Algorithm 1*
	${\mathrm{bdiag}}_{f}\left(H_{M_{D}\times M_{T}}^{\left(RD\right)}\left(f\right)⊗H_{M_{S}\times M_{R}}^{\left(SR\right)}\left(f\right)\right)={\mathrm{bdiag}}_{f}\left({\hat{H}}_{M_{D}M_{S}\times P_{R}J_{R}}^{\left(SRD\right)}\left(f\right)\right){\mathrm{bdiag}}_{f}{\left(C_{P_{R}J_{R}\times M_{T}M_{R}}^{\left(R\right)}\left(f\right)\right)}^{*}$	${\hat{H}}^{\left(RD\right)}\;,\;{\hat{H}}^{\left(SR\right)}$	
**System**	**Unfoldings**	**Estimated parameters**	**Corresp.**
TST			*NTD-6*
	$X_{NM_{D}P_{R}J_{R}\times P_{S}J_{S}}^{\left(D\right)}=\left(S⊗H_{M_{D}P_{R}J_{R}\times M_{S}}^{\left(SRD\right)}\right)C_{RM_{S}\times P_{S}J_{S}}^{\left(S\right)}$		Equation (118)
	$H_{M_{D}M_{S}\times P_{R}J_{R}}^{\left(SRD\right)}=\left(H^{\left(RD\right)}⊗{H^{\left(SR\right)}}^{T}\right)C_{M_{T}M_{R}\times P_{R}J_{R}}^{\left(R\right)}$		Equation (114)
**Receiver**	**Estimation steps**		
KronF	$S⊗H_{M_{D}P_{R}J_{R}\times M_{S}}^{\left(SRD\right)}=X_{NM_{D}P_{R}J_{R}\times P_{S}J_{S}}^{\left(D\right)}{\left(C_{P_{S}J_{S}\times RM_{S}}^{\left(S\right)}\right)}^{*}$	$\hat{S}\;,\;{\hat{H}}_{M_{D}P_{R}J_{R}\times M_{S}}^{\left(SRD\right)}$	Table 20
	$\mathrm{Reshaping}\;\;{\hat{H}}_{M_{D}P_{R}J_{R}\times M_{S}}^{\left(SRD\right)}\to {\hat{H}}_{M_{D}M_{S}\times P_{R}J_{R}}^{\left(SRD\right)}$		*Algorithm 1*
	$H^{\left(RD\right)}⊗{H^{\left(SR\right)}}^{T}={\hat{H}}_{M_{D}M_{S}\times P_{R}J_{R}}^{\left(SRD\right)}{\left(C_{P_{R}J_{R}\times M_{T}M_{R}}^{\left(R\right)}\right)}^{*}$	${\hat{H}}^{\left(RD\right)}\;,\;{\hat{H}}^{\left(SR\right)}$	
**System**	**Unfoldings**	**Estimated parameters**	**Corresp.**
STSTF			*NGTD-5*
	${\mathrm{bdiag}}_{f}\left(X_{NM_{D}P_{R}\times P_{S}}^{\left(D\right)}\left(f\right)\right)=\left[S⊗{\mathrm{bdiag}}_{f}\left(H_{M_{D}P_{R}\times M_{S}}^{\left(SRD\right)}\left(f\right)\right)\right]{\mathrm{bdiag}}_{f}\left(C_{RM_{S}\times P_{S}}^{\left(S\right)}\left(f\right)\right)$		Equation (52)
	${\mathrm{bdiag}}_{f}\left(H_{M_{D}M_{S}\times P_{R}}^{\left(SRD\right)}\left(f\right)\right)={\mathrm{bdiag}}_{f}\left(H_{M_{D}\times M_{T}}^{\left(RD\right)}\left(f\right)⊗H_{M_{S}\times M_{R}}^{\left(SR\right)}\left(f\right)\right){\mathrm{bdiag}}_{f}\left(C_{M_{T}M_{R}\times P_{R}}^{\left(R\right)}\left(f\right)\right)$		Equation (49)
**Receiver**	**Estimation steps**		
KronF	$S⊗{\mathrm{bdiag}}_{f}\left(H_{M_{D}P_{R}\times M_{S}}^{\left(SRD\right)}\left(f\right)\right)={\mathrm{bdiag}}_{f}\left(X_{NM_{D}P_{R}\times P_{S}}^{\left(D\right)}\left(f\right)\right){\mathrm{bdiag}}_{f}{\left(C_{P_{S}\times RM_{S}}^{\left(S\right)}\left(f\right)\right)}^{*}$	$\hat{S}\;,\;{\mathrm{bdiag}}_{f}\left({\hat{H}}_{M_{D}P_{R}\times M_{S}}^{\left(SRD\right)}\left(f\right)\right)$	–
	$\mathrm{Reshaping}\;\;{\mathrm{bdiag}}_{f}\left({\hat{H}}_{M_{D}P_{R}\times M_{S}}^{\left(SRD\right)}\left(f\right)\right)\to {\mathrm{bdiag}}_{f}\left({\hat{H}}_{M_{D}M_{S}\times P_{R}}^{\left(SRD\right)}\left(f\right)\right)$		
	${\mathrm{bdiag}}_{f}\left(H_{M_{D}\times M_{T}}^{\left(RD\right)}\left(f\right)⊗H_{M_{S}\times M_{R}}^{\left(SR\right)}\left(f\right)\right)={\mathrm{bdiag}}_{f}\left({\hat{H}}_{M_{D}M_{S}\times P_{R}}^{\left(SRD\right)}\left(f\right)\right){\mathrm{bdiag}}_{f}{\left(C_{P_{R}\times M_{T}M_{R}}^{\left(R\right)}\left(f\right)\right)}^{*}$	${\hat{H}}^{\left(RD\right)}\;,\;{\hat{H}}^{\left(SR\right)}$	
**System**	**Unfoldings**	**Estimated parameters**	**Corresp.**
STST			*NTD-4*
	$X_{NM_{D}P_{R}\times P_{S}}^{\left(D\right)}=\left(S⊗H_{M_{D}P_{R}\times M_{S}}^{\left(SRD\right)}\right)C_{RM_{S}\times P_{S}}^{\left(S\right)}$		Equation (86)
	$H_{M_{D}M_{S}\times P_{R}}^{\left(SRD\right)}=\left(H^{\left(RD\right)}⊗{H^{\left(SR\right)}}^{T}\right)C_{M_{T}M_{R}\times P_{R}}^{\left(R\right)}$		Equation (87)
**Receiver**	**Estimation steps**		
KronF	$S⊗H_{M_{D}P_{R}\times M_{S}}^{\left(SRD\right)}=X_{NM_{D}P_{R}\times P_{S}}^{\left(D\right)}{\left(C_{P_{S}\times RM_{S}}^{\left(S\right)}\right)}^{*}$	$\hat{S}\;,\;{\hat{H}}_{M_{D}P_{R}\times M_{S}}^{\left(SRD\right)}$	Table 20
	$\mathrm{Reshaping}\;\;{\hat{H}}_{M_{D}P_{R}\times M_{S}}^{\left(SRD\right)}\to {\hat{H}}_{M_{D}M_{S}\times P_{R}}^{\left(SRD\right)}$		*Algorithm 1*
	$H^{\left(RD\right)}⊗{H^{\left(SR\right)}}^{T}={\hat{H}}_{M_{D}M_{S}\times P_{R}}^{\left(SRD\right)}{\left(C_{P_{R}\times M_{T}M_{R}}^{\left(R\right)}\right)}^{*}$	${\hat{H}}^{\left(RD\right)}\;,\;{\hat{H}}^{\left(SR\right)}$	
**System**	**Estimation steps**	**Estimated parameters**	**Corresp.**
STST	${\left({\hat{S}}_{t}\right)}^{T}={\left[\left(I_{P_{S}}⊗\left({\hat{H}}_{t-1}^{\left(RD\right)}⊗I_{P_{R}}\right)C_{M_{T}P_{R}\times M_{R}}^{\left(R\right)}{\hat{H}}_{t-1}^{\left(SR\right)}\right)C_{P_{S}M_{S}\times R}^{\left(S\right)}\right]}^{†}X_{P_{S}M_{D}P_{R}\times N}^{\left(D\right)}$	$\hat{S}$	Equation (82)
**Receiver**	${\left({\hat{H}}_{t}^{\left(RD\right)}\right)}^{T}={\left[\left(I_{P_{R}}⊗\left(I_{P_{S}}⊗{\hat{S}}_{t}\right)C_{P_{S}R\times M_{S}}^{\left(S\right)}{\left({\hat{H}}_{t-1}^{\left(SR\right)}\right)}^{T}\right)C_{P_{R}M_{R}\times M_{T}}^{\left(R\right)}\right]}^{†}X_{P_{R}P_{S}N\times M_{D}}^{\left(D\right)}$	${\hat{H}}^{\left(RD\right)}$	Equation (83)
ALS	$\mathrm{vec}\left({\hat{H}}_{t}^{\left(SR\right)}\right)={\left[\left(\left(I_{P_{S}}⊗{\hat{S}}_{t}\right)⊗\left({\hat{H}}_{t}^{\left(RD\right)}⊗I_{J}\right)\right)\left(C_{P_{S}R\times M_{S}}^{\left(S\right)}⊗C_{M_{T}P_{R}\times M_{R}}^{\left(R\right)}\right)\right]}^{†}x_{P_{S}NM_{D}P_{R}}^{\left(D\right)}$	${\hat{H}}^{\left(SR\right)}$	Equation (84)
**Matrix-based codings—AF protocol**			
**System**	**Unfoldings**	**Estimated parameters**	**Corresp.**
DKRSTF			*NCPD-5*
	$X_{FNM_{D}P_{R}\times P_{S}}^{\left(D\right)}=\left(V_{FN\times M_{S}}^{\left(S\right)}⋄H_{M_{D}P_{R}\times M_{S}}^{\left(SRD\right)}\right){C^{\left(S\right)}}^{T}$ with $V_{FN\times M_{S}}^{\left(S\right)}=\left(A^{\left(S\right)}⋄S\right){W^{\left(S\right)}}^{T}$		Equation (178)
	$H_{M_{D}M_{S}\times P_{R}}^{\left(SRD\right)}=\left(B⋄{H^{\left(SR\right)}}^{T}\right){C^{\left(R\right)}}^{T}$ with $B=H^{\left(RD\right)}W^{\left(R\right)}$		Equation (179)
	$V_{FM_{S}\times N}^{\left(S\right)}=\left(A^{\left(S\right)}⋄W^{\left(S\right)}\right)S^{T}$		Equation (182)
**Receiver**	**Estimation steps**		
KRF	$V_{FN\times M_{S}}^{\left(S\right)}⋄H_{M_{D}P_{R}\times M_{S}}^{\left(SRD\right)}=X_{FNM_{D}P_{R}\times P_{S}}^{\left(D\right)}{C^{\left(S\right)}}^{*}$	${\hat{V}}_{FN\times M_{S}}^{\left(S\right)}\;,\;{\hat{H}}_{M_{D}P_{R}\times M_{S}}^{\left(SRD\right)}$	Equation (180)
	$\mathrm{Reshaping}\;\;{\hat{H}}_{M_{D}P_{R}\times M_{S}}^{\left(SRD\right)}\to {\hat{H}}_{M_{D}M_{S}\times P_{R}}^{\left(SRD\right)}\;$ and $\;{\hat{V}}_{FN\times M_{S}}^{\left(S\right)}\to {\hat{V}}_{FM_{S}\times N}^{\left(S\right)}$		
	$B⋄{H^{\left(SR\right)}}^{T}={\hat{H}}_{M_{D}M_{S}\times P_{R}}^{\left(SRD\right)}{C^{\left(R\right)}}^{*}$	$\hat{B}\;,\;{\hat{H}}^{\left(SR\right)}$	Equation (181)
	${\hat{S}}^{T}={\left(A^{\left(S\right)}⋄W^{\left(S\right)}\right)}^{H}{\hat{V}}_{FM_{S}\times N}^{\left(S\right)}\;\;$ and $\;\;{\hat{H}}^{\left(RD\right)}=\hat{B}{\left(W^{\left(R\right)}\right)}^{H}$	$\hat{S}\;,\;{\hat{H}}^{\left(RD\right)}$	Equation (183)
**System**	**Unfoldings**	**Estimated parameters**	**Corresp.**
SKRST			*NCPD-4*
	$X_{NM_{D}P_{R}\times P_{S}}^{\left(D\right)}=\left(S⋄H_{M_{D}P_{R}\times M_{S}}^{\left(SRD\right)}\right){C^{\left(S\right)}}^{T}$		Equation (72)
	$H_{M_{D}M_{S}\times P_{R}}^{\left(SRD\right)}=\left(H^{\left(RD\right)}⋄{H^{\left(SR\right)}}^{T}\right){C^{\left(R\right)}}^{T}$		Equation (73)
**Receiver**	**Estimation steps**		
KRF	$S⋄H_{M_{D}P_{R}\times M_{S}}^{\left(SRD\right)}=X_{NM_{D}P_{R}\times P_{S}}^{\left(D\right)}{C^{\left(S\right)}}^{*}$	$\hat{S}\;,\;{\hat{H}}_{M_{D}P_{R}\times M_{S}}^{\left(SRD\right)}$	Table 18
	$\mathrm{Reshaping}\;\;{\hat{H}}_{M_{D}P_{R}\times M_{S}}^{\left(SRD\right)}\to {\hat{H}}_{M_{D}M_{S}\times P_{R}}^{\left(SRD\right)}$		
	$H^{\left(RD\right)}⋄{H^{\left(SR\right)}}^{T}={\hat{H}}_{M_{D}M_{S}\times P_{R}}^{\left(SRD\right)}{C^{\left(R\right)}}^{*}$	${\hat{H}}^{\left(RD\right)}\;,\;{\hat{H}}^{\left(SR\right)}$	Table 18
**System**	**Estimation steps**	**Estimated parameters**	**Corresp.**
SKRST	${\left({\hat{H}}_{t}^{\left(RD\right)}\right)}^{T}={\left[C^{\left(R\right)}⋄\left(C^{\left(S\right)}⋄{\hat{S}}_{t-1}\right){\left({\hat{H}}_{t-1}^{\left(SR\right)}\right)}^{T}\right]}^{†}X_{P_{R}P_{S}N\times M_{D}}^{\left(D\right)}$	${\hat{H}}^{\left(RD\right)}$	Equation (67)
**Receiver**	${\left({\hat{S}}_{t}\right)}^{T}={\left[C^{\left(S\right)}⋄\left({\hat{H}}_{t}^{\left(RD\right)}⋄C^{\left(R\right)}\right){\hat{H}}_{t-1}^{\left(SR\right)}\right]}^{†}X_{P_{S}M_{D}P_{R}\times N}^{\left(D\right)}$	$\hat{S}$	Equation (68)
ALS	$\mathrm{vec}\left({\hat{H}}_{t}^{\left(SR\right)}\right)={\left[\left(C^{\left(S\right)}⋄{\hat{S}}_{t}\right)⊗\left({\hat{H}}_{t}^{\left(RD\right)}⋄C^{\left(R\right)}\right)\right]}^{†}x_{P_{S}NM_{D}P_{R}}^{\left(D\right)}$	${\hat{H}}^{\left(SR\right)}$	Equation (70)
**Combined codings—DF protocol**			
**System**	**Unfoldings**	**Estimated parameters**	**Corresp.**
STST-MSMKron	$X_{N_{1}\cdots N_{Q}M_{R}\times P_{S}}^{\left(R\right)}=\left(\overset{Q}{\underset{q=1}{⊗}}S^{\left(q\right)}⊗H^{\left(SR\right)}\right)C_{R_{1}\cdots R_{Q}M_{S}\times P_{S}}^{\left(S\right)}$		Equation (185)
	$X_{N_{1}\cdots N_{Q}M_{D}\times P_{R}}^{\left(D\right)}=\left(\overset{Q}{\underset{q=1}{⊗}}{\hat{S}}^{\left(q\right)}⊗H^{\left(RD\right)}\right)C_{R_{1}\cdots R_{Q}M_{S}\times P_{R}}^{\left(R\right)}$		Equation (186)
**Receiver**	**Estimation steps**		
Multiple KronF	**First hop:** $\;\overset{Q}{\underset{q=1}{⊗}}S^{\left(q\right)}⊗H^{\left(SR\right)}=X_{N_{1}\cdots N_{Q}M_{R}\times P_{S}}^{\left(R\right)}{\left(C_{P_{S}\times R_{1}\cdots R_{Q}M_{S}}^{\left(S\right)}\right)}^{*}$	${\hat{S}}^{\left(1\right)}\;,\cdots ,\;{\hat{S}}^{\left(Q\right)}\;,\;{\hat{H}}^{\left(SR\right)}$	Equation (187)
	**Second hop:** $\;\overset{Q}{\underset{q=1}{⊗}}{\hat{S}}^{\left(q\right)}⊗H^{\left(RD\right)}=X_{N_{1}\cdots N_{Q}M_{D}\times P_{R}}^{\left(D\right)}{\left(C_{P_{R}\times R_{1}\cdots R_{Q}M_{S}}^{\left(R\right)}\right)}^{*}$	${\hat{\hat{S}}}^{\left(1\right)}\;,\cdots ,\;{\hat{\hat{S}}}^{\left(Q\right)}\;,\;{\hat{H}}^{\left(RD\right)}$	Equation (188)
**System**	**Unfoldings**	**Estimated parameters**	**Corresp.**
SKRST-MSMKR	$X_{N_{1}\cdots N_{Q}M_{R}\times P_{S}}^{\left(R\right)}=\left(\overset{Q}{\underset{q=1}{⋄}}S^{\left(q\right)}⋄H^{\left(SR\right)}\right){C^{\left(S\right)}}^{T}$		Equation (189)
	$X_{N_{1}\cdots N_{Q}M_{D}\times P_{R}}^{\left(D\right)}=\left(\overset{Q}{\underset{q=1}{⋄}}{\hat{S}}^{\left(q\right)}⋄H^{\left(RD\right)}\right){C^{\left(R\right)}}^{T}$		Equation (190)
**Receiver**	**Estimation steps**		
Multiple KRF	**First hop:** $\;\overset{Q}{\underset{q=1}{⋄}}S^{\left(q\right)}⋄H^{\left(SR\right)}=X_{N_{1}\cdots N_{Q}M_{R}\times P_{S}}^{\left(R\right)}{C^{\left(S\right)}}^{*}$	${\hat{S}}^{\left(1\right)}\;,\cdots ,\;{\hat{S}}^{\left(Q\right)}\;,\;{\hat{H}}^{\left(SR\right)}$	Equation (191)
	**Second hop:** $\;\overset{Q}{\underset{q=1}{⋄}}{\hat{S}}^{\left(q\right)}⋄H^{\left(RD\right)}=X_{N_{1}\cdots N_{Q}M_{D}\times P_{R}}^{\left(D\right)}{C^{\left(R\right)}}^{*}$	${\hat{\hat{S}}}^{\left(1\right)}\;,\cdots ,\;{\hat{\hat{S}}}^{\left(Q\right)}\;,\;{\hat{H}}^{\left(RD\right)}$	Equation (192)

**Table 29:** Identifiability conditions in terms of design parameters.

Systems/Receivers	Necessary Conditions	Corresp.	Transmission Rates
**Tensor-based codings—AF protocol**			
TSTF/KronF	$P_{R}J_{R}\geq M_{T}M_{R}$, $\;P_{S}J_{S}\geq M_{S}R$	(96)	
TST/KronF	$P_{R}J_{R}\geq M_{T}M_{R}$, $\;P_{S}J_{S}\geq M_{S}R$	(95)	
STSTF/KronF	$P_{R}\geq M_{T}M_{R}$, $\;P_{S}\geq M_{S}R$	(97)	$\left(NR-1\right)/NP_{S}\left(P_{R}+1\right)$
STST/KronF	$P_{R}\geq M_{T}M_{R}$, $\;P_{S}\geq M_{S}R$	(173)	
STST/ALS	$P_{R}P_{S}M_{D}\geq R$, $\;P_{R}P_{S}N\geq M_{T}$, $\;P_{R}P_{S}M_{D}N\geq M_{R}M_{S}$	(85)	
STST/ZF	$P_{R}P_{S}M_{D}\geq R$	(85) *	
**Matrix-based codings—AF protocol**			
DKRSTF/KRF	$P_{S}\geq M_{S}$, $\;P_{R}\geq M_{R}$, $FM_{S}\geq R$, $\;M_{R}\geq M_{T}$	(184)	$\left(N-1\right)R/NP_{S}\left(P_{R}+1\right)$
SKRST/KRF	$P_{S}\geq M_{S}$, $\;P_{R}\geq M_{R}$	(172)	$\left(N-1\right)M_{S}/NP_{S}\left(P_{R}+1\right)$
SKRST/ALS	$P_{R}P_{S}N\geq M_{R}$, $\;P_{R}P_{S}M_{D}\geq M_{S}$, $\;P_{R}P_{S}M_{D}N\geq M_{R}M_{S}$	(71)	
SKRST/ZF	$P_{R}P_{S}M_{D}\geq M_{S}$	(71) *	
**Combined codings—DF protocol**			
STST-MSMKron/KronF	$P_{S}\geq R_{1}\cdots R_{Q}M_{S}$, $\;P_{R}\geq R_{1}\cdots R_{Q}M_{S}$	(193)	$\left(\sum_{q}N_{q}R_{q}-Q\right)/\prod_{q}N_{q}P_{S}\left(P_{R}+1\right)$
SKRST-MSMKR/KRF	$P_{S}\geq M_{S}$, $\;P_{R}\geq M_{S}$	(194)	$\left(\sum_{q}N_{q}-Q\right)M_{S}/\prod_{q}N_{q}P_{S}\left(P_{R}+1\right)$

* Only the condition related to the symbol matrix estimation is considered.

**Table 30:** Values of NMSE of estimated channels $H^{\left(SR\right)}$ and $H^{\left(RD\right)}$ and reconstructed signals $X^{\left(D\right)}$ for SNR = 0 dB.

Systems/Receivers	NMSE		
	$H^{\left(\mathrm{SR}\right)}/H^{\left(\mathrm{SR}\right)}$	$H^{\left(\mathrm{RD}\right)}/H^{\left(\mathrm{RD}\right)}$	$X^{\left(D\right)}$
TSTF/KronF	−12.33	−22.08	−12.09
TST/KronF	−11.27	−23.81	−10.41
STSTF/KronF	−8.33	−16.23	−8.33
STST/KronF	−7.52	−18.02	−6.67
DKRSTF/KRF	−7.48	−20.98	−6.61
SKRST/KRF	−6.73	−18.04	−5.76
STST-MSMKron/KronF	−8.05	−8.06	−6.91
SKRST-MSMKR/KRF	−9.96	−9.90	−8.99

**Table 31:** SNR thresholds for desired SER and NMSE (of reconstructed signals).

		TSTF	TST	STSTF	STST	DKRSTF	SKRST	STST-MSMKron	SKRST-MSMKR
		KronF	KronF	KronF	KronF	KRF	KRF	KronF	KRF
SER	$10^{-2}$	−4 dB	−1 dB	0 dB	2 dB	4 dB	6 dB	−5 dB	−1 dB
	$10^{-3}$	−2 dB	–	–	4 dB	9 dB	12 dB	–	5 dB
NMSE	−10 dB	−2 dB	0 dB	1 dB	3 dB	4 dB	3 dB	3 dB	1 dB
	−20 dB	7 dB	9 dB	11 dB	12 dB	13 dB	12 dB	12 dB	11 dB

**Table 32:** Comparison of systems’ characteristics and receivers’ performance.

Systems/Receivers	Diversities *				Channels		Performance					
	M	P	J	F	FF	FSF	NIC	AK	SER	$H^{\left(\mathrm{SR}\right)}$	$H^{\left(\mathrm{RD}\right)}$	CT
TSTF/KronF	+	+	+	+		+	−−	−−	+++	+++	+++	−−−
TST/KronF	+	+	+		+		−−	−−	++	+++	+++	−−−
STSTF/KronF	+	+		+		+	−−−	−−	++	++	++	−−
STST/KronF	+	+			+		−−−	−−	++	+	++	−−
STST/ALS	+	+			+		−	−−	++	+	++	−−−
DKRSTF/KRF	+	+		+	+		−−	−−−	+	+	+++	−
SKRST/KRF	+	+			+		−−	−−−	+	+	++	−
SKRST/ALS	+	+			+		−	−−−	+	+	++	−−−
STST-MSMKron/KronF	+	+			+		−−−	−	+++	+	+	−−
SKRST-MSMKR/KRF	+	+			+		−−	−−	++	++	+	−

* *M* = antennas, *P* = time-spreading, *J* = chip, *F* = frequency.

## Appendix A. List of Acronyms

**Acronyms**	**Definitions**	**References**
**Tensor models**		
TD	Tucker decomposition	[7]
TTD	Tensor train decomposition	[44]
NTD	Nested TD	[41]
CNTD	Coupled NTD	[42]
DCNTD	Doubly coupled NTD	[43]
GTD	Generalized TD	[38]
NGTD	Nested GTD	–
CPD	Canonical polyadic decomposition	[34]
NCPD	Nested CPD	[39,40]
CCPD	Coupled CPD	[101,102]
CNCPD	Coupled NCPD	–
DCNCPD	Doubly coupled NCPD	[43]
PARALIND	PARAFAC with linearly dependent loadings	[103]
CONFAC	Constrained factor decomposition	[35,36]
BTD	Block-term decomposition	[104]
PARAFAC	Parallel factors decomposition	[8,47]
PARATUCK	PARAFAC-Tucker decomposition	[51,52,53]
CNTD-CPD	Coupled nested TD-CPD	[97]
**Codings**		
STST	Simplified tensor space-time	[41]
STSTF	Simplified tensor space-time-frequency	–
TST	Tensor space-time	[37]
TSTF	Tensor space-time frequency	[38]
SKRST	Simplified Khatri–Rao space-time	[40,53]
DKRSTF	Double Khatri–Rao space-time frequency	[39]
MSMKR	Multiple symbol matrices Khatri–Rao (product)	[98]
MSMKron	Multiple symbol matrices Kronecker (product)	[97]
**Algorithms**		
ALS	Alternating least squares	
KRF	Khatri–Rao factorization	
KronF	Kronecker factorization	
ZF	Zero-forcing	
**Other acronyms**		
AF	Amplify-and-forward	
DF	Decode-and-forward	
SISO	Single-input single-output	
MISO	Multiple-input single-output	
MIMO	Multiple-input multiple-output	
CDMA	Code division multiple access	
OFDM	Orthogonal frequency-division multiplexing	
IRS	Intelligent reflecting surface	
UAV	Unmanned aerial vehicular	
SER	Symbol error rate	
NMSE	Normalized mean square error	

## Appendix B. KRF Method

In this Appendix, we present the Khatri–Rao factorization method, denoted as KRF, to estimate the matrix factors of a Khatri–Rao product, abbreviated as KRP, based on the minimization of an LS criterion, first proposed in [49].

Let us consider the case of the noisy KRP: Y=A⋄B+E of two matrices $A=\left[a_{1},\cdots ,a_{R}\right]\in K^{I\times R}$ and $B=\left[b_{1},\cdots ,b_{R}\right]\in K^{J\times R}$, where E is an additive noise matrix due to modeling error and measurement noise, with the following LS criterion to be minimized:

(A1) $$\min_{A,B}{∥Y-A⋄B∥}_{F}^{2}.$$

By vectorizing Y, this criterion can be rewritten as follows:

(A2) $$\min_{A,B}{∥\mathrm{vec}\left(Y\right)-\mathrm{vec}\left(A⋄B\right)∥}_{2}^{2}=\underset{r\in \langle R\rangle }{\min_{a_{r},b_{r}}}\sum_{r=1}^{R}{∥y_{r}-a_{r}⋄b_{r}∥}_{2}^{2}.$$

Since each term in this sum can be minimized separately, the column vectors $a_{r}\in K^{I}$ and $b_{r}\in K^{J}$ are estimated by minimizing $\min_{a_{r},b_{r}}{∥y_{r}-a_{r}⋄b_{r}∥}_{2}^{2}$.

The idea behind the KRF method is to form a rank-one matrix by unvectorizig each KRP $y_{r}$ for r∈〈R〉, as

(A3) $$Y_{r}≜\mathrm{unvec}\left(y_{r}\right)=\mathrm{unvec}\left(a_{r}⋄b_{r}\right)=b_{r}a_{r}^{T}=b_{r}\circ a_{r}\in K^{J\times I}$$

and to calculate the rank-one approximation of this matrix using the reduced SVD algorithm. The vectors $\left(a_{r},b_{r}\right)$ are determined via a simple identification of the formed rank-one matrix with the left and right singular vectors associated with the largest singular value. The LS criterion is then replaced with a rank-one matrix approximation:

(A4) $$\min_{a_{r},b_{r}}∥Y_{r}-b_{r}a_{r}^{T}{∥}_{F}^{2}=\min_{a_{r},b_{r}}{∥Y_{r}-b_{r}\circ a_{r}∥}_{F}^{2}\;\;\;,\;\;\;r\in \langle R\rangle ,$$

where ∘ denotes the outer product. The vectors $a_{r}$ and $b_{r}$ are estimated by computing the rank-one reduced SVD of $Y_{r}$:

(A5) $$Y_{r}=\sigma_{r}^{\left(1\right)}u_{r}^{\left(1\right)}{\left(v_{r}^{\left(1\right)}\right)}^{H},$$

where $\sigma_{r}^{\left(1\right)}$ denotes the largest singular value, and $u_{r}^{\left(1\right)}$ and $v_{r}^{\left(1\right)}$ are the left and right singular vectors associated with $\sigma_{r}^{\left(1\right)}$, respectively. Identifying the reduced SVD (A5) with $b_{r}a_{r}^{T}$ leads to the following estimates [49]:

(A6) $${\hat{a}}_{r}=\sqrt{\sigma_{r}^{\left(1\right)}}{v_{r}^{\left(1\right)}}^{*}\;\;\;,\;\;\;{\hat{b}}_{r}=\sqrt{\sigma_{r}^{\left(1\right)}}u_{r}^{\left(1\right)}.$$

Note:

- The *R* columns of A and B can be estimated in parallel by computing the rank-one approximation of *R* matrices;
- Each vector, $a_{r}$ and $b_{r}$, r∈〈R〉, is only estimated up to a scaling factor, since $\left(\lambda_{r}a_{r}\right)⋄\left(\frac{1}{\lambda_{r}}b_{r}\right)=a_{r}⋄b_{r}$ for every $\lambda_{r}\in K$. To eliminate this scaling ambiguity, we need to know one component of one of these two vectors. For example, if we assume that the first component ${\left(a_{r}\right)}_{1}$ is equal to 1, then $\lambda_{r}=\frac{1}{{\left({\hat{a}}_{r}\right)}_{1}}$, and the ambiguity is removed as follows:
  (A7) $${\hat{\hat{a}}}_{r}=\frac{1}{{\left({\hat{a}}_{r}\right)}_{1}}{\hat{a}}_{r}\;\;\;,\;\;\;{\hat{\hat{b}}}_{r}={\left({\hat{a}}_{r}\right)}_{1}{\hat{b}}_{r},$$
  where ${\hat{a}}_{r}$ and ${\hat{b}}_{r}$ are defined in (A6). We can conclude that the factors A and B of a Khatri–Rao product are estimated without scaling ambiguity if we have a priori knowledge of one row of A or B. This follows from the fact that $A\Lambda ⋄B\Lambda^{-1}=A⋄B$ for any non-singular matrix $\Lambda =\mathrm{diag}\left(\lambda_{1},\cdots ,\lambda_{R}\right)\in K^{R\times R}$.

We now present a generalization of the KRF method to the case of a multiple KRP [33]:

(A8) $$\min_{A^{\left(n\right)},n\in \langle N\rangle }{∥Y-\overset{N}{\underset{n=1}{⋄}}A^{\left(n\right)}∥}_{F}^{2},$$

with $A^{\left(n\right)}=\left[a_{1}^{\left(n\right)},\cdots ,a_{R}^{\left(n\right)}\right]\in K^{I_{n}\times R}$, n∈〈N〉. This generalization is based on the rank-one approximation of *R* tensors of order *N* constructed from the columns of the factors $A^{\left(n\right)}$. Let us consider the column $y_{r}=\overset{N}{\underset{n=1}{⋄}}a_{r}^{\left(n\right)}\in K^{I_{1}\cdots I_{N}}$, for r∈〈R〉, and the associated rank-one tensor $X_{r}=\overset{N}{\underset{n=1}{\circ }}a_{r}^{\left(n\right)}\in K^{I_{1}\times \cdots \times I_{N}}$ defined by the transformation $y_{r}\to X_{r}$ such as:

(A9) $${\left(X_{r}\right)}_{i_{1},\cdots ,i_{N}}={\left(y_{r}\right)}_{\bar{i_{1}\cdots i_{N}}}\;,$$

where

(A10) $$\bar{i_{1}\cdots i_{N}}≜i_{N}+\sum_{n=1}^{N-1}\left(i_{n}-1\right)\prod_{k=n+1}^{N}I_{k}.$$

As for the case of the KRP of two vectors, the LS criterion (A8) is replaced with:

(A11) $$\min_{a_{r}^{\left(n\right)},n\in \langle N\rangle }∥y_{r}-\overset{N}{\underset{n=1}{⋄}}a_{r}^{\left(n\right)}{∥}_{F}^{2}=\min_{a_{r}^{\left(n\right)},n\in \langle N\rangle }{∥X_{r}-\overset{N}{\underset{n=1}{\circ }}a_{r}^{\left(n\right)}∥}_{F}^{2}\;\;\;,\;\;\;r\in \langle R\rangle .$$

The columns $a_{r}^{\left(n\right)}$ are then determined in calculating the rank-one approximation of the tensor $X_{r}$ by means of the truncated higher-order SVD (THOSVD) algorithm [48]. Like for a Khatri–Rao product of two vectors, the *R* columns of the factors $A^{\left(n\right)}$ can be estimated in parallel.

Noting that $\overset{N}{\underset{n=1}{\circ }}\lambda_{n}a_{r}^{\left(n\right)}=\overset{N}{\underset{n=1}{\circ }}a_{r}^{\left(n\right)}$ for any $\lambda_{n}$ such that $\overset{N}{\prod_{n=1}}\lambda_{n}=1$, one concludes that each estimated column vector $a_{r}^{\left(n\right)}$ is unique up to a scalar ambiguity. To eliminate these ambiguities, one component of N−1 columns among the *N* vectors $a_{r}^{\left(n\right)},n\in \langle N\rangle ,$ must be known.

## Appendix C. KronF Method

We now consider the Kronecker factorization method, denoted as KronF, to estimate the matrix factors of a multiple Kronecker product, denoted as KronP, in minimizing the following LS cost function:

(A12) $$\min_{A^{\left(n\right)},n\in \langle N\rangle }{∥Y-\overset{N}{\underset{n=1}{⊗}}A^{\left(n\right)}∥}_{F}^{2},$$

with $A^{\left(n\right)}\in K^{I_{n}\times J_{n}}$, n∈〈N〉.

Inspired by the approach proposed in [105] to solve the problem of approximating a given matrix by a simple Kronecker product using a SVD-based rank-one matrix approximation, a generalization to a multiple Kronecker product is obtained in constructing a rank-one tensor from Y, defined as the outer product of vectorized forms $\mathrm{vec}\left(A^{\left(n\right)}\right)\in K^{J_{n}I_{n}}$ of the matrices $A^{\left(n\right)}$:

(A13) $$X=\overset{N}{\underset{n=1}{\circ }}\mathrm{vec}\left(A^{\left(n\right)}\right)\in K^{J_{1}I_{1}\times J_{2}I_{2}\times \cdots \times J_{N}I_{N}}.$$

The problem of estimating the factors $A^{\left(n\right)}$ can be solved in the sense of minimizing the following LS criterion [33]:

(A14) $$\min_{A^{\left(1\right)},\cdots ,A^{\left(N\right)}}∥Y-\overset{N}{\underset{n=1}{⊗}}A^{\left(n\right)}{∥}_{F}^{2}=\min_{A^{\left(1\right)},\cdots ,A^{\left(N\right)}}{∥X-\overset{N}{\underset{n=1}{\circ }}\mathrm{vec}\left(A^{\left(n\right)}\right)∥}_{F}^{2}.$$

This minimization amounts to finding the best rank-one approximation for the tensor X using the THOSVD method. This approximation gives an estimate of the vectorized forms $\mathrm{vec}(A^{\left(n\right)})$, from which it is easy to deduce an estimate of the factors $A^{\left(n\right)}$ via a simple unvectorization operation. Since, for ${\tilde{A}}^{\left(n\right)}=\lambda_{n}A^{\left(n\right)},n\in \langle N\rangle$, we have $\overset{N}{\underset{n=1}{\circ }}\mathrm{vec}\left({\tilde{A}}^{\left(n\right)}\right)=\overset{N}{\underset{n=1}{\circ }}\mathrm{vec}\left(A^{\left(n\right)}\right)$ if $\prod_{n=1}^{N}\lambda_{n}=1$, we deduce that each estimate $\mathrm{vec}(A^{\left(n\right)})$ is subject to a scalar scaling ambiguity $\lambda_{n}$ requiring knowledge of one component to eliminate this ambiguity.

## Appendix D. Complexity of the Receiver Algorithms

The computational complexity of the proposed semi-blind receivers is evaluated using Big-O notation, considering the most computationally expensive operations. For KronF/KRF-based methods, the dominant cost comes from SVD-based rank-1 approximations, while for ALS methods, it is associated with pseudo-inverse computations. The computational complexities of the proposed receivers are summarized in Table A1, assuming $P_{S}=P_{R}=P$, $J_{S}=J_{R}=J$, $M_{S}=M_{R}=M_{T}=M_{D}=M$, $N_{1}=\cdots =N_{Q}=N$, and $R_{1}=\cdots =R_{Q}=R$.

It is important to note that, for ALS receivers, the complexity is evaluated for a single iteration. The total computational cost is consequently dependent on the number of iterations required for convergence.

In this analysis, we consider that the computational complexity of the KronF and KRF algorithms, when applied to an $I_{1}\times I_{2}$ matrix, is $O(I_{1}I_{2})$, corresponding to the computation of a rank-1 SVD. For the ALS algorithms, the complexity of the pseudo-inverse calculation is $O(I_{1}I_{2}^{2})$ when $I_{1}\geq I_{2}$, or $O(I_{1}^{2}I_{2})$ when $I_{1}\leq I_{2}$.

**Table A1:** Complexity of the receivers.

System/Receiver	Matrix Dimension	Complexity
**Tensor-based codings-AF protocol**		
TSTF/KronF	$S⊗{\mathrm{bdiag}}_{f}\left(H_{M_{D}P_{R}J_{R}\times M_{S}}^{\left(SRD\right)}\left(f\right)\right)\Rightarrow F\left(NM_{D}P_{R}J_{R}\times RM_{S}\right)$	$O(FNM^{2}PJR)$
	${\mathrm{bdiag}}_{f}\left(H_{M_{D}\times M_{T}}^{\left(RD\right)}\left(f\right)⊗H_{M_{S}\times M_{R}}^{\left(SR\right)}\left(f\right)\right)\Rightarrow F\left(M_{D}M_{S}\times M_{T}M_{R}\right)$	$O(FM^{4})$
TST/KronF	$S⊗H_{M_{D}P_{R}J_{R}\times M_{S}}^{\left(SRD\right)}\Rightarrow NM_{D}P_{R}J_{R}\times RM_{S}$	$O(NM^{2}PJR)$
	$H^{\left(RD\right)}⊗{H^{\left(SR\right)}}^{T}\Rightarrow M_{D}M_{S}\times M_{T}M_{R}$	$O(M^{4})$
STSTF/KronF	$S⊗{\mathrm{bdiag}}_{f}\left(H_{M_{D}P_{R}\times M_{S}}^{\left(SRD\right)}\left(f\right)\right)\Rightarrow F\left(NM_{D}P_{R}\times RM_{S}\right)$	$O(FNM^{2}PR)$
	${\mathrm{bdiag}}_{f}\left(H_{M_{D}\times M_{T}}^{\left(RD\right)}\left(f\right)⊗H_{M_{S}\times M_{R}}^{\left(SR\right)}\left(f\right)\right)\Rightarrow F\left(M_{D}M_{S}\times M_{T}M_{R}\right)$	$O(FM^{4})$
STST/KronF	$S⊗H_{M_{D}P_{R}\times M_{S}}^{\left(SRD\right)}\Rightarrow NM_{D}P_{R}\times RM_{S}$	$O(NM^{2}PR)$
	$H^{\left(RD\right)}⊗{H^{\left(SR\right)}}^{T}\Rightarrow M_{D}M_{S}\times M_{T}M_{R}$	$O(M^{4})$
STST/ALS	${\left[\left(I_{P_{S}}⊗\left({\hat{H}}_{t-1}^{\left(RD\right)}⊗I_{P_{R}}\right)C_{M_{T}P_{R}\times M_{R}}^{\left(R\right)}{\hat{H}}_{t-1}^{\left(SR\right)}\right)C_{P_{S}M_{S}\times R}^{\left(S\right)}\right]}^{†}\Rightarrow P_{S}M_{D}P_{R}\times R$	$O(MP^{2}R^{2})$
	${\left[\left(I_{P_{R}}⊗\left(I_{P_{S}}⊗{\hat{S}}_{t}\right)C_{P_{S}R\times M_{S}}^{\left(S\right)}{\left({\hat{H}}_{t-1}^{\left(SR\right)}\right)}^{T}\right)C_{P_{R}M_{R}\times M_{T}}^{\left(R\right)}\right]}^{†}\Rightarrow P_{R}P_{S}N\times M_{T}$	$O(NM^{2}P^{2})$
	${\left[\left(\left(I_{P_{S}}⊗{\hat{S}}_{t}\right)⊗\left({\hat{H}}_{t}^{\left(RD\right)}⊗I_{J}\right)\right)\left(C_{P_{S}R\times M_{S}}^{\left(S\right)}⊗C_{M_{T}P_{R}\times M_{R}}^{\left(R\right)}\right)\right]}^{†}\Rightarrow P_{S}NM_{D}P_{R}\times M_{R}M_{S}$	$O(NM^{5}P^{2})$
**Matrix-based codings-AF protocol**		
DKRSTF/KRF	$V_{FN\times M_{S}}^{\left(S\right)}⋄H_{M_{D}P_{R}\times M_{S}}^{\left(SRD\right)}\Rightarrow FNM_{D}P_{R}\times M_{S}$	$O(FNM^{2}P)$
	$B⋄{H^{\left(SR\right)}}^{T}\Rightarrow M_{D}M_{S}\times M_{R}$	$O(M^{3})$
SKRST/KRF	$S⋄H_{M_{D}P_{R}\times M_{S}}^{\left(SRD\right)}\Rightarrow NM_{D}P_{R}\times M_{S}$	$O(NM^{2}P)$
	$H^{\left(RD\right)}⋄{H^{\left(SR\right)}}^{T}\Rightarrow M_{D}M_{S}\times M_{R}$	$O(M^{3})$
SKRST/ALS	${\left[C^{\left(R\right)}⋄\left(C^{\left(S\right)}⋄{\hat{S}}_{t-1}\right){\left({\hat{H}}_{t-1}^{\left(SR\right)}\right)}^{T}\right]}^{†}\Rightarrow P_{R}P_{S}N\times M_{R}$	$O(NM^{2}P^{2})$
	${\left[C^{\left(S\right)}⋄\left({\hat{H}}_{t}^{\left(RD\right)}⋄C^{\left(R\right)}\right){\hat{H}}_{t-1}^{\left(SR\right)}\right]}^{†}\Rightarrow P_{S}M_{D}P_{R}\times M_{S}$	$O(M^{3}P^{2})$
	${\left[\left(C^{\left(S\right)}⋄{\hat{S}}_{t}\right)⊗\left({\hat{H}}_{t}^{\left(RD\right)}⋄C^{\left(R\right)}\right)\right]}^{†}\Rightarrow P_{S}NM_{D}P_{R}\times M_{S}M_{R}$	$O(NM^{5}P^{2})$
**Combined codings-DF protocol**		
STST-MSMKron/KronF	$\overset{Q}{\underset{q=1}{⊗}}S^{\left(q\right)}⊗H^{\left(SR\right)}\Rightarrow N_{1}\cdots N_{Q}M_{R}\times R_{1}\cdots R_{Q}M_{S}$	$O(N^{Q}M^{2}R^{Q})$
	$\overset{Q}{\underset{q=1}{⊗}}{\hat{S}}^{\left(q\right)}⊗H^{\left(RD\right)}\Rightarrow N_{1}\cdots N_{Q}M_{D}\times R_{1}\cdots R_{Q}M_{S}$	$O(N^{Q}M^{2}R^{Q})$
SKRST-MSMKR/KRF	$\overset{Q}{\underset{q=1}{⋄}}S^{\left(q\right)}⋄H^{\left(SR\right)}\Rightarrow N_{1}\cdots N_{Q}M_{R}\times M_{S}$	$O(N^{Q}M^{2})$
	$\overset{Q}{\underset{q=1}{⋄}}{\hat{S}}^{\left(q\right)}⋄H^{\left(RD\right)}\Rightarrow N_{1}\cdots N_{Q}M_{D}\times M_{S}$	$O(N^{Q}M^{2})$
